# Metal-organic framework membranes with single-atomic centers for photocatalytic CO_2_ and O_2_ reduction

**DOI:** 10.1038/s41467-021-22991-7

**Published:** 2021-05-11

**Authors:** Yu-Chen Hao, Li-Wei Chen, Jiani Li, Yu Guo, Xin Su, Miao Shu, Qinghua Zhang, Wen-Yan Gao, Siwu Li, Zi-Long Yu, Lin Gu, Xiao Feng, An-Xiang Yin, Rui Si, Ya-Wen Zhang, Bo Wang, Chun-Hua Yan

**Affiliations:** 1 Ministry of Education Key Laboratory of Cluster Science, Beijing Key Laboratory of Photoelectronic/Electrophotonic Conversion Materials, School of Chemistry and Chemical Engineering, Beijing Institute of Technology, Beijing, P. R. China; 2 Beijing National Laboratory for Molecular Sciences, State Key Laboratory of Rare Earth Materials Chemistry and Applications, PKU-HKU Joint Laboratory in Rare Earth Materials and Bioinorganic Chemistry, College of Chemistry and Molecular Engineering, Peking University, Beijing, P. R. China; 3 Shanghai Synchrotron Radiation Facility, Shanghai Institute of Applied Physics, Chinese Academy of Sciences, Shanghai, P. R. China; 4 Institute of Physics, Chinese Academy of Sciences, Beijing, P. R. China; 5 Advanced Technology Research Institute (Jinan), Beijing Institute of Technology, Jinan, P. R. China

**Keywords:** Metal-organic frameworks, Photocatalysis, Nanoscale materials

## Abstract

The demand for sustainable energy has motivated the development of artificial photosynthesis. Yet the catalyst and reaction interface designs for directly fixing permanent gases (e.g. CO_2_, O_2_, N_2_) into liquid fuels are still challenged by slow mass transfer and sluggish catalytic kinetics at the gas-liquid-solid boundary. Here, we report that gas-permeable metal-organic framework (MOF) membranes can modify the electronic structures and catalytic properties of metal single-atoms (SAs) to promote the diffusion, activation, and reduction of gas molecules (e.g. CO_2,_ O_2_) and produce liquid fuels under visible light and mild conditions. With Ir SAs as active centers, the defect-engineered MOF (e.g. activated NH_2_-UiO-66) particles can reduce CO_2_ to HCOOH with an apparent quantum efficiency (AQE) of 2.51% at 420 nm on the gas-liquid-solid reaction interface. With promoted gas diffusion at the porous gas-solid interfaces, the gas-permeable SA/MOF membranes can directly convert humid CO_2_ gas into HCOOH with a near-unity selectivity and a significantly increased AQE of 15.76% at 420 nm. A similar strategy can be applied to the photocatalytic O_2_-to-H_2_O_2_ conversions, suggesting the wide applicability of our catalyst and reaction interface designs.

## Abstract (web summary)

Photoreduction of permanent gas faces challenges in reactant diffusion and activation at the three-phase interface. Here the authors showed porous metal-organic framework membranes decorated by metal single atoms can boost the photoreduction of CO_2_ and O_2_ at the high-throughput gas-solid interface.

## Introduction

In nature, solar energy is harnessed by photosynthesis and stored in fossil fuels through slow geochemical fossilization processes. Resembling the functionalities of green leaves, interdisciplinary researchers have developed various photosynthetic systems across the natural–artificial spectrum^1–8^. Although a solar-to-electric energy conversion rate of over 20% can be achieved by inorganic photovoltaic devices^9^, abiotic photosynthetic systems that can fix naturally abundant permanent gases (e.g., CO_2_, O_2_) into value-added liquid fuels still face challenges in productivity and selectivity^6,10^.

First, challenged by the large kinetic barriers, both homogeneous and heterogeneous photocatalysts feature inherent advantages and trade-offs for such applications^11^. On one hand, homogeneous catalysts possess highly dispersed and accessible active sites with accurately tailored structures for specific reactions but poor product isolation and/or long-term stability^12,13^. On the other hand, conventional heterogenous approaches exhibit enhanced product separation, but suffer from smaller active surface areas and limited accessibility to the active sites^11^. One promising strategy bridging homogeneous and heterogeneous photocatalysis is to develop catalysts with atomically dispersed active centers^14–17^, which can significantly improve the accessibility and utilization of catalytic sites while facilitate the separation of products. For instance, photocatalysts with atomically dispersed active centers can effectively reduce CO_2_ to CO^15–17^, suggesting the advantages of such single-atom photocatalysts (SAPCs); while systematic studies are still in great need to reveal the mechanism for reducing CO_2_ on single-atomic sites to produce a much wider range of value-added products (e.g., hydrocarbons, alcohols, and carboxylates, etc.) with higher selectivity and efficiency.

Second, the efficiency for reducing such permanent gas is further limited by the gas–liquid–solid reaction interface. The traditional photocatalytic interface design with dissolving or submerging catalysts in aqueous solutions is optimal for reactions involving only liquid reactants (e.g., water splitting)^4^. The limited solubility and sluggish diffusion kinetics of permanent gas in aqueous solutions strongly retard the following catalytic conversion on the heterogeneous reaction interface^18,19^. In addition, the overwhelming competitions from water adsorption on the flooded catalyst surface further suppress the reduction of gas molecules with the undesired hydrogen evolution reaction (HER)^6,19,20^. Consequently, photoreduction of insoluble and stable gas molecules on the three-phase catalysis interface inevitably confronts three formidable challenges: (i) the kinetically hindered diffusion, (ii) the sluggish surface adsorption, and (iii) the inefficient activation and catalytic conversion of the gas reactants. Fabricating films of stacked nanoparticles, including solid semiconductors^21–23^ and porous metal-organic frameworks (MOFs)^24,25^, and catalyzing photocatalytic gas reduction on the gas-solid interfaces with water vapor as proton sources could partially solve the mass transfer limitation by avoiding the water flooding of the catalyst surface. However, such systems still suffer from slow mass transfer with the only driving force from spontaneous thermal motions of gaseous molecules. Therefore, to further promote the photoreduction of permanent gas to storable and portable chemical fuels, advanced photocatalytic systems should consist of both highly dispersed active sites lowering the reaction energy barriers and high-throughput reaction interfaces, with large surface areas boosting the diffusion of gaseous reactants to the active sites.

**Fig. 1:**
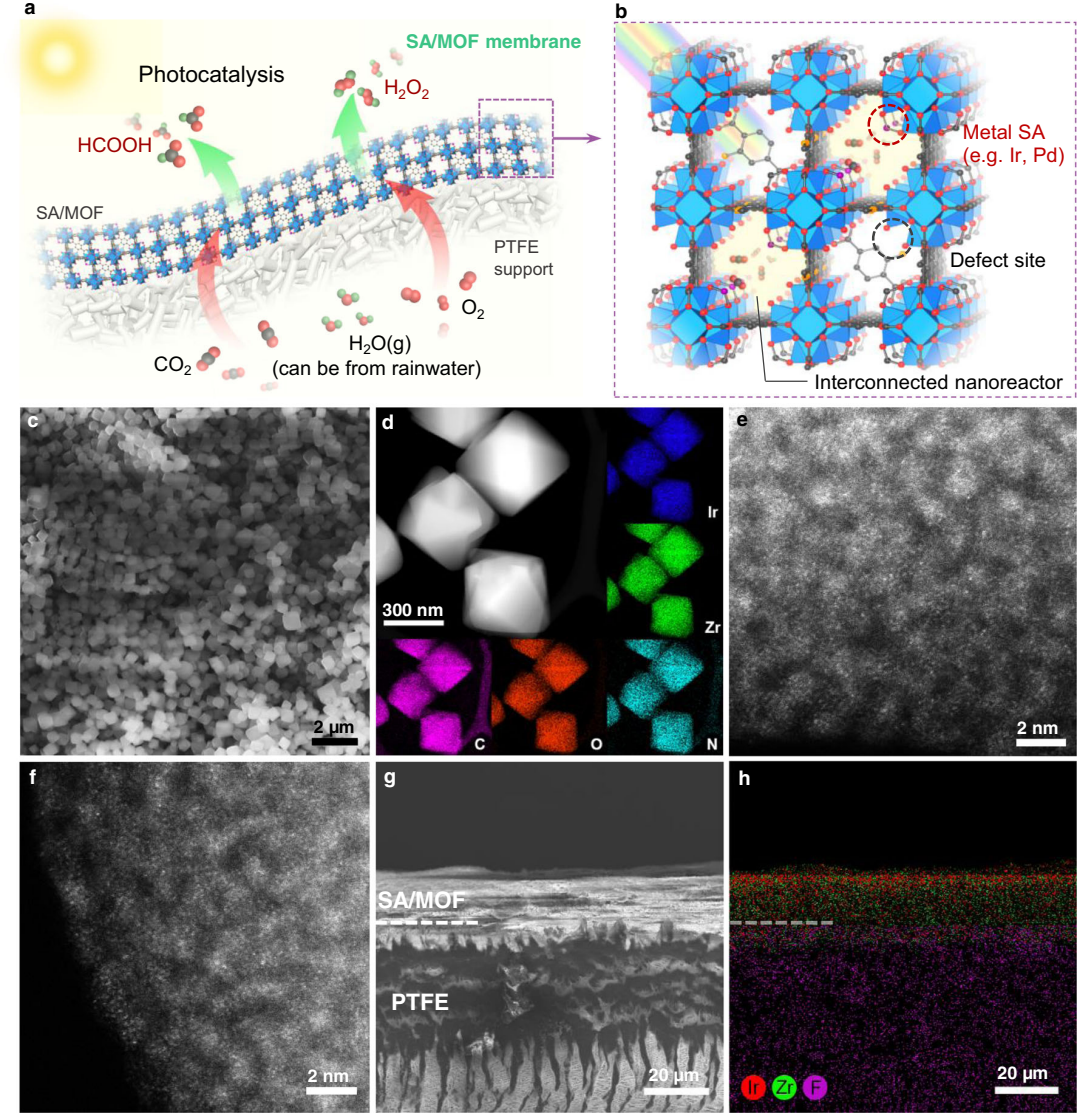
Schematic illustration and structural characterization of the SA/MOF membranes. **a** Humidified gases (e.g., CO_2_, O_2_) can be fed through the gas-permeable MOF/PTFE membranes and photocatalytically reduced to value-added chemicals (e.g., HCOOH and H_2_O_2_) under visible light irradiation and ambient conditions. **b** With controllable defect-engineering, specific metal SAs (e.g., Ir and Pd) can be precisely anchored on the edges of the Zr_6_O_4_(OH)_4_(–CO_2_)_12−x_ octahedral to act as programable catalytic centers for catalyzing different reactions, such as photocatalytic CO_2_-to-HCOOH and O_2_-to-H_2_O_2_ conversion. The open and interconnected MOF pores serve as the nanoreactors facilitating the diffusion and conversion of gas reactants. **c** SEM image for Ir_1_/A-aUiO particles. **d** HAADF-STEM image and corresponding EDS maps for Ir_1_/A-aUiO particles. **e**, **f** AC-HAADF-STEM images for **e** Ir_1_/A-aUiO and **f** Pd_1_/A-aUiO, indicating the atomic dispersion of metal species in A-aUiO matrixes. **g**, **h** SEM image (**g**) and corresponding EDS mapping (**h**) for the cross section of Ir_1_/A-aUiO/PTFE membranes.

MOFs, an emerging class of porous crystalline materials with large surface areas, high porosity, and tunable functionalities, prove to be a promising candidate for heterogeneous photocatalytic reductions^26–29^, showing great potential for solving the aforementioned challenges in both reaction kinetics and mass transfer process. Here, combining the advantages of porous MOF substrates and single-atom (SA) catalytic centers, we introduce gas-permeable membranes consisting of structurally tailored SA/MOFs particles for high-efficiency and high-throughput photocatalytic gas reduction reactions (Fig. 1a, b). Single-atom iridium (Ir_1_) and palladium (Pd_1_) are anchored on the MOF nodes to serve as the catalytic hydrogenation centers for CO_2_ and O_2_, respectively (Fig. 1b). The defect-engineering of MOF matrix can not only enhance their light harvesting capability but also tailor the chemical structures of their oxide nodes (e.g., the Zr–O notes of NH_2_-UiO-66) to finely control their interactions to the introduced metal single atoms (SAs). The activated NH_2_-UiO-66 (denoted as A-aUiO hereafter) substrate can tailor the electronic structures of the supported metal species to enhance their catalytic reactivity. For instance, with stronger meal-supporter interactions (SMSI), Ir_1_/A-aUiO exhibited a near-unity HCOOH evolution selectivity in photocatalytic CO_2_ reduction, while Ir nanoparticles supported by A-aUiO (IrNPs/A-aUiO) showed a much lower HCOOH selectivity of 16.5%. Moreover, the high porosity of SA/MOF membranes allows the creation of gas-membrane-gas (GMG) configuration, which boosts the high-throughput diffusion of humid CO_2_ to the metal SAs located at the vast gas-solid reaction interfaces within the interconnected MOF pores. Compared with the traditional gas–liquid–solid reaction interface, such high-throughput gas–solid interface design can significantly increase the availability of gaseous reactants in the vicinity of each catalytic centers (e.g., SAs). Consequently, bridging advanced catalytic center and reaction interface designs, our Ir_1_/A-aUiO membranes can catalyze the CO_2_-to-HCOOH photoreduction with near-unity selectivity and a HCOOH activity of 3.38 mmol g_cat_^–1^ h^–1^ (the term “cat” refers to the hybrid metal species and supporters, such as metal/MOFs and metal/oxides, hereafter), exceeding 6.5 and 338 times that of the Ir_1_/A-aUiO particles and the IrNPs/A-aUiO particles, respectively. Moreover, following similar optimization strategy, the Pd_1_/A-aUiO membranes can convert humid O_2_ to H_2_O_2_ with an activity of 10.4 mmol g_cat_^–1^ h^–1^ under visible light, which is more than 73-fold higher than that showed by the PdNPs/A-aUiO (0.14 mmol g_cat_^–1^ h^–1^) powders, verifying the wide applicability of our catalyst and reaction interface designs.

## Results and discussion

### Catalyst synthesis

We screened a series of photosensitizers, including MOFs, inorganic semiconductors, and coordination complex, as the hosts of metal SA active centers and the building blocks of our photocatalysis membranes. Among these candidates, NH_2_-UiO-66 (aUiO), a kind of representative MOF materials^30^, represented a promising candidate with unique advantages such as high photoactivity, porosity, specific surface area, and water stability^30,31^. Especially, the versatile chemical tunability of such MOF materials allowed us to modify their chemical structures, and light harvesting capabilities at the molecular level for anchoring and sensitizing external metal species with rationally designed catalytic properties^32,33^. Typically, monodispersed aUiO particles were prepared by solution method with the ligands (2-aminoterephthalic acid) partially replaced by acetates (Supplementary Fig. 1). These acetates were removed afterwards by activating the as-obtained aUiO particles under elevated temperature in a vacuum oven to obtain A-aUiO with missing linkers, and abundant defects on the edges of the Zr_6_O_4_(OH)_4_(–CO_2_)_12−x_ octahedral secondary building units (SBUs, where *x* represents number of missing linkers per SBU)^34^ (Supplementary Fig. 2). Compared with the pristine aUiO particles, such defects provided abundant anchoring sites to bond the subsequently introduced metal (e.g., Ir and Pd) species to obtain our SA/MOF particles and membranes (Supplementary Figs. 3–11).

Selected metal single-atoms (SAs) (e.g., Ir and Pd) were then incorporated into the A-aUiO particles by impregnation and annealing treatments (see “Methods” section). For instance, Ir SAs (1.4 wt%) could be highly dispersed in A-aUiO matrix to form Ir_1_/A-aUiO particles, as revealed together by the scanning electron microscopy (SEM) image, high-angle annular dark-field scanning TEM (HAADF-STEM) image, and corresponding energy-dispersive X-ray spectroscopy (EDS) maps, as well as aberration-corrected HAADF-STEM (AC-HAADF-STEM) images (Fig. 1c–e and Supplementary Fig. 4). Extended X-ray absorption fine structure (EXAFS) analysis exhibited the existence of Ir–O bonds with the average coordination number of 3.8 and the absence of Ir–Ir bonding (Supplementary Fig. 5 and Table 1), further verifying the single-atomic dispersion of Ir species in the Ir_1_/A-aUiO particles. Lowering the Ir loading to 0.7 wt% resulted in sparser dispersion of Ir SAs in Ir_1_/A-aUiO (Supplementary Figs. 6 and 7), while increasing the loading of Ir to 2.7 wt.% and annealing the hybrids under reducing atmosphere (H_2_/Ar, 1/4 v/v) led to the formation of A-aUiO containing both Ir SAs and small clusters (Ir_x_/A-aUiO, Supplementary Figs. 8 and 9). Similarly, Pd (0.8 wt%) could also be atomically dispersed in A-aUiO to form Pd_1_/A-aUiO, as confirmed by AC-HAADF-STEM and EXAFS results (Fig. 1f and Supplementary Figs. 10 and 11).

These SA/A-aUiO particles were then deposited onto commercially available porous polytetrafluoroethylene (PTFE) films in a layer-by-layer manner to fabricate flexible and gas-permeable membranes through facile filtration protocols (Fig. 1g, h and Supplementary Figs. 12–16). The hierarchical channels of the PTFE films (Fig. 1g) and the interconnected pores of the SA/A-aUiO particles naturally resembled the stomata of green leaves, facilitating the direct diffusion of gas molecules and the collision of them onto the open metal SA catalytic centers within the pores and channels of MOFs.

### Photocatalytic CO_2_ reduction reaction (CO_2_RR)

**Fig. 2:**
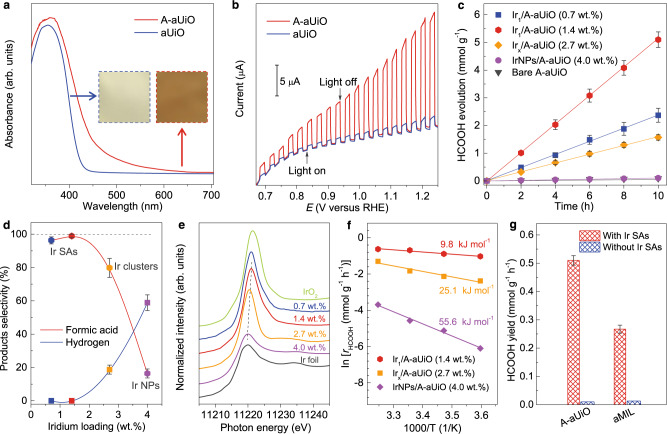
Photocatalytic CO_2_RR on SA/MOF powder catalysts. **a**, **b** The enhancing of light harvesting and conversion efficiency for A-aUiO through defects engineering. UV–Vis diffusive reflectance spectra (**a**) and photocurrent-potential curves (**b**) for A-aUiO and a-UiO samples, respectively. Inset in **a**: Digital photograph showing the color of aUiO and A-aUiO samples. **c** Time course of HCOOH evolution on A-aUiO, Ir_1_/A-aUiO, Ir_x_/A-aUiO and IrNPs/A-aUiO catalysts. **d** HCOOH and H_2_ selectivity as a function of Ir loadings in Ir/A-aUiO catalysts. **e** Ir L-edge XANES spectra of Ir/A-aUiO catalysts with different Ir loadings. Ir foil and IrO_2_ were used as standards. The first maximum of the XANES curves after the absorption edge (dashed line) shows gradual positive movement with decreasing Ir loading, suggesting the metallicity of the Ir species was gradual decreased. **f** Apparent activation energy (*E*_app_) of various catalysts for HCOOH generation. **g** HCOOH yields on A-aUiO and aMIL catalysts with and without Ir SAs modification. All the photocatalytic reactions were performed under visible light (>420 nm) irradiation and using isopropanol (20 v/v% in water) as sacrificial agents. Error bars were calculated by carrying out three parallel catalysis reactions.

We then performed photocatalytic CO_2_RR at the traditional three-phase (with Ir_1_/A-aUiO particles) and our re-designed reaction interfaces (with Ir_1_/A-aUiO membranes), to demonstrate the advantages of single-atomic catalytic centers and high-throughput gas–solid reaction interfaces. Water and isopropanol were used as the proton sources and sacrificial agents, respectively. A 300 W Xe lamp equipped with a 420 nm long-pass filter was used as the visible light source. As revealed by ultraviolet-visible absorption spectra (Fig. 2a), creating missing linkers and coordination defects on the Zr–O nodes dramatically enhanced the visible light absorption efficiency of aUiO particles (Supplementary Fig. 17)^35^, shifting their absorption band edge up to 600 nm in the visible region. Compared with the pristine defect-deficient aUiO and other typical semiconductor photocatalytic hosts, such as titanium dioxide (TiO_2_) and carbon nitride (C_3_N_4_), A-aUiO exhibited much higher photon-to-electron conversion efficiency under visible light (>420 nm) irradiation (Fig. 2b and Supplementary Figs. 18 and 19). The photogenerated electrons could be then effectively captured by active centers such as metal SAs and nanoparticles (NPs) (Supplementary Fig. 20).

To investigate the intrinsic activity and catalytic mechanism of our catalysts, we first monitored the CO_2_RR on powder photocatalysts in the conventional particle-in-solution (PiS) mode with gas–liquid–solid reaction interfaces (Supplementary Fig. 21). As shown in Fig. 2c, bare A-aUiO showed no observable CO_2_RR activity. The IrNPs/A-aUiO catalysts (with an optimized metal loading content of 4.0 wt%, Supplementary Figs. 22 and 23) exhibited a HCOOH generation activity of 10 µmol g_cat_^–1^ h^–1^ (i.e., 10 µmol of HCOOH per gram of metal/A-aUiO catalysts per hour) with a poor selectivity of only 16.5% (Fig. 2c and Supplementary Fig. 24). The Ir_x_/A-aUiO samples, containing both Ir clusters and SAs, showed higher HCOOH evolution activity (0.16 mmol g_cat_^–1^ h^–1^) and selectivity (79.8%) (Fig. 2c, d). In sharp contrast, the 0.7 wt% Ir_1_/A-aUiO catalysts exhibited a HCOOH generation rate of 0.24 mmol g_cat_^–1^ h^–1^ with a high selectivity of 96.3%. Increasing the Ir SAs loading to 1.4 wt% elevate the HCOOH activity to as high as 0.51 mmol g_cat_^–1^ h^–1^ with a near-unity selectivity (Supplementary Figs. 24–26). The significant differences in CO_2_RR reactivity among iridium SAs, clusters, and NPs (all supported by A-aUiO) suggested that the chemical structures of Ir species and their interactions with the A-aUiO hosts played a critical role in the selective photoreduction of CO_2_ to HCOOH. Compared with Ir clusters and nanoparticles, the Ir SAs were more positively charged (+3 to +4) as revealed by X-ray absorption near-edge structure (XANES) and X-ray photoelectron spectroscopy (XPS) analysis (Fig. 2e and Supplementary Fig. 27). Such differences in electronic structures may thereby result in their different catalytic reactivity. According to the Arrhenius plots (Fig. 2f), the Ir_1_/A-aUiO catalyst showed much lower apparent activation energy (*E*_app_) for HCOOH evolution (9.8 kJ mol^–1^) than Ir_x_/A-aUiO (25.1 kJ mol^–1^) and IrNPs/A-aUiO (55.6 kJ mol^–1^), suggesting the much faster CO_2_RR kinetics on Ir_1_/A-aUiO under similar conditions. Thus, the SMSI could substantially modulate the electronic structures of Ir species, and the positively charged Ir SAs presented the best CO_2_RR activity and selectivity targeting HCOOH production.

The reaction route for CO_2_RR on the Ir_1_/A-aUiO catalyst was further studied by isotope-labeled mass spectroscopy (MS) and in situ Fourier-transform infrared spectroscopy (FTIR). As revealed by the electrospray ionization-mass spectrometry (ESI-MS) (Supplementary Fig. 28), H^13^COO^−^ was the only reduction product when ^13^CO_2_ was used as feed gas, confirming that the formates were generated from photocatalytic CO_2_RR exclusively. Additionally, as shown in Supplementary Fig. 29, FTIR peaks (at 1497 and 1433 cm^–1^) of the surface carbonic acid species^36,37^, the intermediate species for formic acid evolution^36^, were immediately observed when humidified CO_2_ was fed into the reactors containing Ir_1_/A-aUiO or A-aUiO catalysts in dark, indicating the adsorption of CO_2_ by the hydroxyls on the Zr–O nodes of A-aUiO^38,39^. The reactors were then purged with Ar to remove the excess CO_2_. Under visible light irradiation, new infrared peaks (at 2920, 2850, and 1576 cm^–1^) were observed on the Ir_1_/A-aUiO catalyst (Supplementary Fig. 30a), suggesting the formation of HCOO* species upon irradiation^40–42^. To the contrary, no significant HCOO* peaks could be found in the FTIR spectra for the bare A-aUiO particles (Supplementary Fig. 30b). In addition, as shown in Supplementary Fig. 17, the conduction band of A-aUiO (–0.60 V vs. NHE) is located more negative than the standard electrode potential of CO_2_/HCOOH (–0.37 V vs. NHE)^43^. Therefore, a plausible reaction route for photocatalytic CO_2_RR on Ir_1_/A-aUiO would be: the CO_2_ molecules were first adsorbed by the A-aUiO to form surface carbonic acid species, and then hydrogenated on the Ir_1_ catalytic centers with photogenerated electrons provided by the A-aUiO matrix and protons (from water) to form HCOOH^44,45^. Meanwhile, the photo-generated holes could be reduced by the sacrifice agent (i.e., isopropanol, Supplementary Fig. 31) to facilitate the transfer of electrons to reduce CO_2_ species. Interestingly, similar photocatalytic CO_2_RR route for HCOOH production can be also enabled on other Ir_1_/MOF catalysts. For instance, NH_2_-MIL-125(Ti) ref. ^46^ (aMIL) decorated by Ir SAs (Ir_1_/aMIL, Supplementary Fig. 32) showed significantly higher CO_2_RR activity than the pristine aMIL components. Under visible light irradiation, the HCOOH evolution rates were 0.27 and 0.013 mmol g_cat_^–1^ h^–1^, with the *E*_app_ of 20.1 and 58.7 kJ mol^–1^on Ir_1_/aMIL and bare aMIL, respectively (Fig. 2g and Supplementary Fig. 33). Therefore, the synergy between the MOF matrix and the Ir SA active centers contributes to CO_2_RR for HCOOH evolution with high activity and selectivity.

### Reaction interface design

**Fig. 3:**
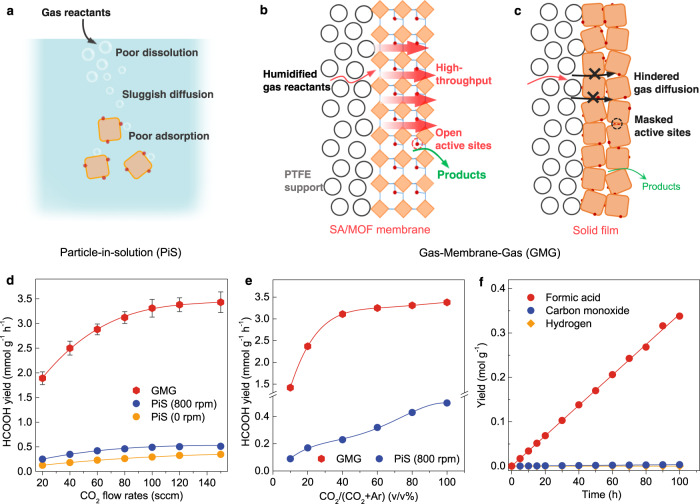
Photocatalytic CO_2_RR on SA/MOF membranes. **a** In the particle-in-solution (PiS) mode, gas-phase CO_2_ was fed into the aqueous photocatalyst dispersion and then reduced on the water flooded surfaces of catalysts. **b** In the gas-membrane-gas (GMG) mode, humidified CO_2_ gas was fed through the SA/MOF membrane, where water vapor was carried by the CO_2_ stream. **c** Schematic illustration for the solid films fabricated by non-porous semiconductor particles. **d** HCOOH yields on Ir_1_/A-aUiO particles (PiS) and membrane (GMG) under different CO_2_ flow rates, respectively. **e** HCOOH yields on Ir_1_/A-aUiO particles (PiS) and membrane (GMG) under different CO_2_ concentration (in Ar mixtures), respectively. **f** Time course of HCOOH, CO and H_2_ evolution on Ir_1_/A-aUiO membrane (GMG). All the photocatalytic reactions were performed under visible light (>420 nm). The CO_2_ flow rates in **e** and **f** were fixed at 120 sccm. Error bars represent the standard deviation for three independent catalysis tests.

Along with the catalytic center optimization, fabricating porous SA/MOF membranes allows us to build novel gas–solid reaction interfaces that can break the mass transfer limitation for gas reactants to further promote CO_2_RR. Compared with the PiS mode in which the CO_2_ availability in the vicinity of catalyst surfaces is restricted by their low solubility and sluggish diffusion in aqueous solutions (Fig. 3a), the updated gas-membrane-gas (GMG) (Fig. 3b, Supplementary Figs. 34, and 35) configuration can significantly decrease the diffusional lengths and resistance for gas reactants^19^. As a result, the molecular ratio of CO_2_/H_2_O in the vicinity of each catalytic centers can be increased by 4 orders of magnitude from 1/1600 (saturated CO_2_ solutions, PiS) to ~30/1 (CO_2_ gas with saturated H_2_O steam, GMG)^18^. The increase of CO_2_ availability and the reverse of CO_2_/H_2_O ratio in the SA/MOF membranes would thus dramatically enhance the CO_2_RR activity in a wide range of CO_2_ flow rates (Fig. 3d). For instance, with the CO_2_ flow rate fixed at 120 standard cubic centimeters per minute (sccm), the Ir_1_/A-aUiO membrane exhibited a HCOOH yield of 3.38 mmol g_cat_^–1^ h^–1^ (i.e., 3.38 mmol of HCOOH per gram of Ir_1_/A-aUiO catalysts per hour) in the GMG mode, exceeding more than six times that showed by Ir_1_/A-aUiO particles (0.51 mmol g_cat_^–1^ h^–1^) in the PiS mode (Fig. 3d and Supplementary Table 2). The advantages for such catalyst and interface design were further demonstrated by the apparent quantum efficiency (AQE) measurements at various wavelengths. As shown in Supplementary Fig. 36, the Ir_1_/A-aUiO membranes exhibited AQE values of 15.76 and 6.40% at 420 and 475 nm, respectively, which are much higher than that showed by the Ir_1_/A-UiO particles in the conventional PiS mode (2.51% and 1.10%, respectively). In addition, ESI-MS studies further indicated that the collected formats in the GMG mode were also exclusively generated by photocatalytic CO_2_RR (Supplementary Fig. 37).

The advantages of the SA/MOF membranes and the high-throughput GMG reaction interfaces were further demonstrated by photocatalysis performances with different CO_2_ availability. As shown in Fig. 3e, in the PiS mode, the HCOOH evolution rate increased gradually with the volume fractions of CO_2_ concentrations in the mixture of CO_2_ and Ar [i.e., CO_2_/(CO_2_ + Ar), v/v%]. To the contrary, the HCOOH yield increased rapidly and reached to the plateau at low CO_2_ concentrations (<40 v/v%) in the GMG mode. Notably, in the relatively low CO_2_ concentration region (<60 v/v%), the GMG configuration exhibited an order of magnitude higher HCOOH generation rate compared with the conventional PiS mode. These results further confirm that the Ir_1_/A-aUiO membranes can enable efficient gas mass transfer, thereby enrich CO_2_ with relatively low initial concentrations, and turn them into HCOOH with much higher efficiency than the powder catalysts dispersed in aqueous solutions. In addition, compared with the GMG mode, the identical 1.4 wt% Ir_1_/A-UiO membranes exhibited a much smaller formate yield of 0.76 mmol g_cat_^–1^ h^–1^ in the spontaneous diffusion mode (Supplementary Fig. 38), where the mass transfer was driven by only spontaneous thermal motion of gaseous molecules^24,25^. Therefore, addressing the mass transfer limitation encountered by the PiS mode (Fig. 3a) and the spontaneous diffusion mode (Supplementary Fig. 38), the Ir_1_/A-aUiO membranes (GMG) exhibited near constant photocatalytic CO_2_RR activity and selectivity (3.38 mmol g_cat_^–1^ h^–1^ and ~98% at the CO_2_ flow rate of 120 sccm) through the long-term (100 h) operation (Fig. 3f), showing high stability with no significant changes observed in the recycled Ir_1_/A-aUiO photocatalysts (Supplementary Fig. 39).

Further control experiments suggested the vital role of the highly porous MOF matrix in constructing such high-throughput gas–solid catalytic interfaces. The porous Ir_1_/A-aUiO and Ir_1_/aMIL membranes exhibited a 5.6-fold and 4.8-fold enhancement in CO_2_RR activity as compared with their powder counterparts, respectively; however, the solid films composed of non-porous inorganic particles (e.g., Ir/TiO_2_, Ir/ZnO) showed a much lower activity enhancement (~40%) (Supplementary Figs. 40–44). These significant differences in enhancement factors for CO_2_RR on MOF membranes, and solid films may arise from their markedly different mass transfer and photoelectron deliver efficiencies (Fig. 3b, c). Thanks to the high porosity and open pore structure of MOF matrixes, the diffusion of CO_2_/H_2_O molecules from gas phase to the highly dispersed active centers would be efficient in high throughput (Supplementary Fig. 45). The delivery of photoelectrons from the local ligands to the active sites also not be hindered by stacking SA/MOF particles. However, the aggregation of solid semiconductor particles would form a solid film that would retard the efficient diffusion of gas molecules to the surface active sites (Fig. 3c and Supplementary Fig. 45), further suppressing the photocatalytic reduction of CO_2_ on such solid films.

The use of water vapor instead of aqueous solutions as proton sources brought additional advantages for CO_2_RR. As shown in Supplementary Fig. 46, in the PiS mode, the HCOOH evolution rate significantly declined when tap water or rainwater (both collected on the BIT campus in Beijing) was used as proton sources instead of the ultrapure DI water. The decrease in catalyst activity would be possibly caused by the impurities in tap water or rainwater (e.g., Cl^–^, ClO^–^ and other ions), which may poison the active centers. However, using the naturally purified water vapor as proton sources could effectively avoid the poisoning of catalyst from such impurities, and ensure the HCOOH yield despite the types of water sources. Therefore, our SA/MOF membranes and GMG interface designs exhibited great potential in fixing relatively low-concentration CO_2_ to HCOOH with high selectivity and activity, using naturally available water as proton sources and visible light as energy supplies.

### Photocatalytic oxygen reduction reaction

**Fig. 4:**
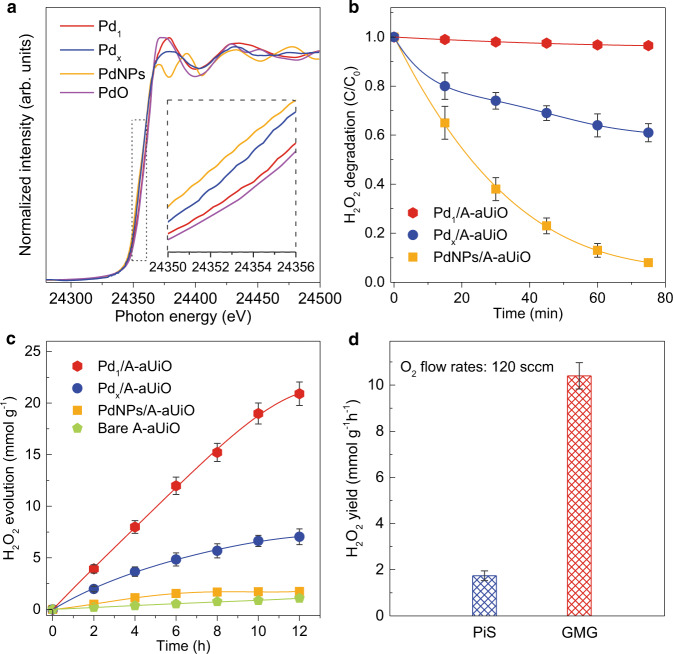
Photocatalytic O_2_ reduction (ORR) on Pd modified MOF powders and membrane. **a** Pd K-edge XANES spectra of PdNPs, Pd_x_, and Pd_1_ decorated A-aUiO catalysts. PdO was used as standard. **b** Time course of H_2_O_2_ decomposition on PdNPs, Pd_x_, and Pd_1_ decorated A-aUiO catalysts. **c** Time course of H_2_O_2_ generation on pristine A-aUiO and PdNPs, Pd_x_, and Pd_1_ decorated A-aUiO catalysts. **d** H_2_O_2_ yields on Pd_1_/A-aUiO particles (PiS) and membranes (GMG), respectively. All the photocatalytic reactions were performed under visible light (>420 nm) irradiation and using isopropanol (20 v/v% in water) as sacrificial agents. The O_2_ flow rates were fixed at 120 sccm. Error bars represent the standard deviation for three independent catalysis tests.

Notably, the wide applicability of our catalyst and reaction interface design strategy can be further applied to the photoreduction of O_2_ to H_2_O_2_ (Fig. 4). Like Ir/A-aUiO, the strong metal-substrate interactions could also modulate the electronic structures of Pd species (e.g., Pd SAs, clusters, and nanoparticles) supported by A-aUiO matrix (Fig. 4a and Supplementary Figs. 47 and 48). As compared with the Pd clusters and nanoparticles, the positively charged Pd_1_ sites could not only significantly promote H_2_O_2_ generation but also effective suppress H_2_O_2_ decomposition^47^ (Fig. 4b and Supplementary Fig. 49). As a result, in the PiS mode, Pd_1_/A-aUiO particles exhibited an H_2_O_2_ yield (1.74 mmol g_cat_^–1^ h^–1^) that was more than 3 and 12 times that of Pd_x_/A-aUiO (0.58 mmol g_cat_^–1^ h^–1^) and PdNPs/A-aUiO (0.14 mmol g_cat_^–1^ h^–1^), respectively (Fig. 4c). Additional control experiments confirmed that the detected H_2_O_2_ was produced by reducing O_2_ exclusively. As shown in Supplementary Fig. 50, no H_2_O_2_ production could be detected in experiments without light irradiation, O_2_ feed, or catalysts (i.e., Pd_1_/A-aUiO). Like the CO_2_RR case, further enhancement in H_2_O_2_ generation activity could be realized by reacting humidified O_2_ gas flow on Pd_1_/A-aUiO membrane in the GMG mode, showing a high H_2_O_2_ evolution rate (10.4 mmol g_cat_^–1^ h^–1^) that was more than 4.9-fold higher than that observed in the PiS mode (Fig. 4d and Supplementary Table 3). That is, the gas-permeable MOF (e.g., A-aUiO, aMIL) membranes can serve as a versatile array of photosensitizer and nanoreactor to host different metal SAs (e.g., Ir, Pd), for the selective photocatalytic reduction of specific gases into chemical fuels under visible light and mild conditions.

The highly porous SA/MOF membranes decorated with single-atomic reaction centers, assembling numerous regularly arranged and interconnected photocatalytic nanoreactors, bridge the best of homogeneous and heterogeneous catalysis. On one hand, like molecular catalysts, the explicit structures for the highly dispersed photocatalyst modules (ligand–node–SA) allow us to modify their light harvesting, electron delivery, and catalytic conversion properties with atomic precision. On the other hand, the porous but rigid MOF crystals hold all the nanoreactors together via stable coordination bonds with interconnected pores, ensuring the high stability, dispersity, and accessibility of the atomically dispersed catalytic centers. Especially, starting with humidified gas instead of gas solutions, the novel GMG reaction interfaces not only facilitate the diffusion of gas molecules but also eliminate the competitive adsorption of liquid water, which is inevitable in conventional three-phase photocatalytic gas reduction reactions. The substantial increase in local concentration and molar ratio of gas reactants in the vicinity of each catalytic center, thereby promote the activity and selectivity of photocatalytic reduction of gas molecules significantly.

In practice, our SA/MOF membranes can directly reduce CO_2_ and O_2_ into value-added and ready-to-use chemical fuels (i.e., HCOOH and H_2_O_2_). With specifically designed catalytic centers, the major components of air (CO_2_, O_2_, and even N_2_) could be fixed via sustainable photocatalytic reactions, with water vapor as proton donors under mild conditions to convert and store the intermittent solar energy. The facile preparation of SA/MOF membranes facilitates the scale-up of such photocatalysis devices, and the minimized usage of water vapor saves the cost of freshwater in arid regions with rich solar powers. Our work provides a general and programmable strategy bridging photocatalysis center, and reaction interface designs to solve the complexity for mass transfer and catalytic conversion in three-phase photocatalytic reactions. The powerful toolbox in single-atom and MOF chemistry endows us with almost infinite possibilities to engineer the chemical structures of SA/MOF membranes. Such design may find wider applicability in more substantial heterogeneous reactions.

## Methods

### Preparation of NH_2_-UiO-66 (aUiO) particles

In a typical synthesis, 10 mg of anhydrous zirconium chloride (ZrCl_4_) was dissolved in 5 mL of N,N-Dimethylformamide (DMF) in a 20 mL glass vial. In another vial, 15 mg of 2-aminoterephthalic acid (NH_2_-BDC) was also dissolved in 5 mL of DMF. Then, the two solutions were mixed before 1.3 mL of acetic acid was added. The resulting solution was shaken and then heated in a 120 °C isothermal for 12 h. Light yellow powders were collected by centrifugation, washed five times with DMF over a period of 48 h, and five times with methanol over a period of 36 h. Finally, light yellow aUiO powder was obtained by drying the products under the dynamic vacuum overnight at room temperature.

### Preparation of defect-rich NH_2_-UiO-66 (A-aUiO) particles

The as-prepared aUiO powders with partial acetate ligands were activated under high dynamical vacuum and high temperature to remove the acetate residuals and obtain A-aUiO. Typically, 120 mg of the as-prepared aUiO powders were put into a quartz crucible and kept in a vacuum oven at 100 °C for 8 h. Then, the oven temperature was raised to 180 °C at a rate of 2 °C min^–1^ and maintained at 180 °C for 28 h. Finally, brownish yellow powder was obtained after cooling down to room temperature.

### Preparation of Ir_1_/A-aUiO (1.4 wt%)

In a typical synthesis, 80 mg of A-aUiO powders were fully dispersed by 60 mL of ultrapure water. Then, under vigorous stirring, 1.3 mL of K_2_IrCl_6_ solution (5 mM) was slowly injected (1 mL min^–1^) into the above dispersion with an injection pump. The mixture was stirred at 500 rpm under 40 °C for 12 h. After that, the products were collected via centrifugation and washed by ultrapure water. After drying in the vacuum oven under 80 °C, the products were put into a quartz boat and heated to 200 °C at a rate of 5 °C min^–1^ and maintained at 200 °C for 1 h in a tubular oven under high-purity (99.9999%) Ar protection (60 sccm). The resultant powders were cooled down to room temperature and collected for further characterizations and tests. The preparation of 0.7 wt% Ir_1_/A-aUiO particles was similar but with K_2_IrCl_6_ solution of lower concentration (2.5 mM).

### Preparation of Ir_x_/A-aUiO

In a typical synthesis, 60 mg of A-aUiO powders were fully dispersed by 45 mL of ultrapure water. Then, 1.3 mL of K_2_IrCl_6_ solution (7.5 mM) was injected (1 mL min^–1^) into the above dispersion with an injection pump. The mixture was stirred at 500 rpm under 40 °C for 12 h. After that, the products were collected via centrifugation and washed by ultrapure water (two times). After drying in the vacuum oven under 80 °C, the products were put into a quartz boat and heated to 250 °C at a rate of 5 °C min^–1^ and maintained at 250 °C for 1 h in a tubular oven under H_2_/Ar (1/4 v/v, 60 sccm).

### Preparation of IrNPs/A-aUiO

In a typical synthesis, 60 mg of A-aUiO powders were fully dispersed in 45 mL of ultrapure water. Then, 1.3 mL of K_2_IrCl_6_ solution (15 mM) was injected into the above dispersion. The mixture was stirred at 500 rpm under room temperature for 1 h. After that, the products were collected via centrifugation and washed by ultrapure water. After drying in the vacuum oven under 80 °C, the products were put into a quartz boat and heated to 300 °C at a rate of 5 °C min^–1^ and maintained at 300 °C for 2 h in a tubular oven under H_2_/Ar (1/4 v/v, 60 sccm).

### Preparation of Ir_1_/aMIL

Typically, 655 mg of NH_2_-BDC, 6.5 mL of anhydrous DMF, 1.2 mL of anhydrous methanol and 0.312 mL of Ti(OiPr)_4_ were added into a 15 mL Teflon-lined autoclave. After 0.5 h of sonication, the autoclave was sealed and heated at 130 °C for 15 h. Light yellow powders were collected by centrifugation, washed five times with DMF over a period of 48 h, and five times with methanol over a period of 24 h. Finally, MIL-125(NH_2_) (aMIL) powders were obtained by drying and activating under 160 °C and dynamic vacuum for 24 h. After that, 80 mg of the activated aMIL powders were dispersed by 60 mL of ultrapure water. Then, under vigorous stirring, 1.3 mL of K_2_IrCl_6_ solution (5 mM) was injected (1 mL min^–1^) into the above dispersion with an injection pump. The mixture was stirred at 500 rpm under 40 °C for 12 h. The products were collected via centrifugation and washed by ultrapure water (two times). After drying in the vacuum oven under 80 °C, the product was put into a quartz boat and heated to 200 °C at a rate of 5 °C min^–1^, and maintained at 200 °C for 1 h in a tubular oven under high-purity (99.9999%) Ar protection (60 sccm).

### Preparation of Pd_1_/A-aUiO

Eighty milligram of A-aUiO powders were fully dispersed in 60 mL of ultrapure water. Then, 1.3 mL of Na_2_PdCl_4_ aqueous solution (5 mM) was slowly injected (0.5 mL/min) into the above dispersion with an injection pump. The mixture was then stirred under room temperature for 14 h. After that, the products were collected via centrifugation and washed by ultrapure water (two times). After drying in the vacuum oven under 80 °C, the products were put into a quartz boat and heated to 200 °C at a rate of 5 °C min^–1^ and maintained at 200 °C for 1 h in a tubular oven under high-purity (99.9999%) Ar protection (60 sccm). The resultant powders were cooled down to room temperature and collected for further characterizations and tests.

### Preparation of Pd_x_/A-aUiO

Sixty milligram of A-aUiO powders were fully dispersed in 60 mL of ultrapure water. Then, 1.3 mL of Na_2_PdCl_4_ aqueous solution (7.5 mM) was slowly injected (1 mL/min) into the above dispersion with an injection pump. The mixture was then stirred under room temperature for 14 h. After that, the products were collected via centrifugation and washed by ultrapure water (two times). After drying in the vacuum oven under 80 °C, the products were put into a quartz boat and heated to 200 °C at a rate of 5 °C min^–1^ and maintained at 200 °C for 1 h in a tubular oven under high-purity (99.9999%) Ar protection (60 sccm). The resultant powders were cooled down to room temperature and collected for further characterizations and tests.

### Preparation of PdNPs/A-aUiO (1.3 wt%)

In a typical synthesis, 60 mg of A-aUiO powders were fully dispersed in 45 mL of ultrapure water. Then, 1.3 mL of Na_2_PdCl_4_ solution (15 mM) was injected into the above dispersion. The mixture was stirred at 500 rpm under room temperature for 1 h. After that, the products were collected via centrifugation and washed by ultrapure water. After drying in the vacuum oven under 80 °C, the products were put into a quartz boat and heated to 300 °C at a rate of 5 °C min^–1^ and maintained at 300 °C for 2 h in a tubular oven under H_2_/Ar (1/4 v/v, 80 sccm).

### Preparation of Ir/TiO_2_ (0.42 wt%) and Ir/ZnO (0.40 wt%)

Sixty milligram of commercially available TiO_2_ (Acros) or ZnO (Acros) powders were fully dispersed in 45 mL ultrapure water. Then, 1.3 mL of K_2_IrCl_6_ aqueous solution (5 mM) was slowly injected into the above dispersion. The mixture was then stirred at 500 rpm under room temperature for 12 h. After drying in the vacuum oven under 80 °C, the products were put into a quartz boat and heated to 300 °C at a rate of 5 °C min^–1^ and maintained at 300 °C for 1 h in a tubular oven under high-purity (99.9999%) Ar protection (60 sccm). The resultant powders were cooled down to room temperature and collected for further characterizations and tests.

### Fabrication of leaf-like SA/MOF membranes

Typically, 12.5 mg of SA/MOF powders (Ir_1_/A-aUiO, Pd_1_/A-aUiO or Ir_1_/aMIL) were first dispersed in a mixture of isopropanol, ultrapure water and Nafion solution (150 μL of Nafion solution in 8 mL of 7:1 isopropanol:water mixture). The mixture was then sonicated for 30 min to produce SA/MOF inks. Then the inks were deposited onto one side of commercially available polytetrafluoroethylene (PTFE) films to fabricate the 15-μm-thick membranes through vacuum filtration. After that, the as-prepared membranes were dried in Ar and activated in high dynamical vacuum at 80 °C.

### Fabrication of TiO_2_ and ZnO-based solid films

About 20 mg of Ir/TiO_2_ or Ir/ZnO powders were dispersed in a mixture of isopropanol, water, and Nafion solution (150 μL of Nafion solution in 8 mL of 7:1 isopropanol:water mixture). The mixture was then sonicated for 1.5 h to produce catalysts inks. Then the inks were deposited onto one side of the polytetrafluoroethylene (PTFE) films to fabricate the 15-μm-thick membranes through vacuum filtration. After that, the as-prepared membranes were dried in vacuum under 80 °C.

### Characterization

XRD measurements of the obtained catalyst powders were performed on a Rigaku MiniFlex 600 diffractometer with a Cu-Kα X-ray radiation source (*λ* = 0.154056 nm). Typically, 2 mg of powders were placed on an amorphous silica substrate and the XRD patterns were record at a scan rate of 2° min^–1^. XPS measurements were performed by a Thermo VG ESCALAB-250 system with Al-Kα and Mg-Kα source operated at 15 kV. The binding energies were referred to the C 1*s* peak (284.8 eV) from adventitious carbon. In situ FTIR spectra were recorded a Bruker ALPHA spectrometer. The one-dimensional ^1^H spectra were recorded on Bruker ARX-400 and Bruker ARX-700 spectrometers. ESI-MS results were recorded on a PE SCIEX API 150 mass spectrometer. In the tests, the solution was continuously infused with a syringe pump at a constant flow rate into the pneumatically assisted electrospray probe with N_2_ as the nebulizing gas. N_2_ sorption isotherms were measured at 77 K on a Quantachrome ASiQMVH002-5 absorption apparatus. Before tests, the samples were pre-activated at 120 °C for 12 h. The pore size distributions were estimated by the DFT method from a N_2_ sorption experiment at 77 K. The metal loading in our catalysts was determined by an ICP-AES spectrometer (Model Optima 2000, PerkinElmer). A series of solutions for the measurements were prepared by dissolving 20 mg of samples in 4 mL of aqua regia (75 vol.% HCl and 25 vol.% HNO_3_). The solution was left overnight to allow complete dissolution. The resultant solution was diluted to 50 mL with deionized water in a volumetric flask and then analysed using ICP-AES. UV–Vis DRS spectra were recorded by a UV-2600 (Shimadzu) spectrophotometer in the wavelength range of 300–800 nm, using BaSO_4_ as reference. The steady-state PL spectra were measured by an F-4500 spectrophotometer (Hitachi). The transient-state PL spectra were carried out on an FLSP920 spectrophotometer (Edinburgh Instruments). The ESR measurements were performed out on a JEOL FA-200 micro spectrometer at 90 K. Samples were placed into NMR tubes and cooled to 90 K using liquid nitrogen stream for measurements.

The TEM images were obtained on a JEM-2100 transmission electron microscope operating at 200 kV. The samples were prepared by dropping water/ethanol dispersion of samples onto ultrathin carbon film and immediately evaporating the solvent. The high-angle annular dark-field scanning TEM (HAADF-STEM) images were obtained on a TECNAI F30 transmission electron microscope operating at 300 kV. The aberration-corrected high-angle annular dark-field scanning TEM (AC-HAADF-STEM) images and STEM-EDX elemental mapping were collected on a JEM-ARM200F transmission electron microscopy working at 200 kV, equipped with a probe spherical aberration corrector. The SEM images and EDX elemental mapping were acquired from JEOL S-4800 and Zeiss Supra 55 scanning electron microscopes.

The Ir L-edge XAFS spectra were obtained at beamline BL14W1 of the Shanghai Synchrotron Radiation Facility (SSRF). The samples were measured in fluorescence mode by using a 32-element Ge solid state detector to collect the data. Iridium foil (Ir), iridium chloride (IrCl_3_), and iridium oxide (IrO_2_) were used as standard reference materials for these measurements. The Pd K-edge XAFS spectra were obtained at beamline BL14W1 of the Shanghai Synchrotron Radiation Facility (SSRF), and beamline BL01C1 of the National Synchrotron Radiation Research Center (NSRRC). The samples were measured in fluorescence mode by using a solid-state detector to collect the data. Palladium foil (Pd), and palladium oxide (PdO) were used as standard reference materials for these measurements. Athena and Artemis codes were used to extract the data and fit the profiles. For the X-ray absorption near edge structure (XANES) part, the experimental absorption coefficients as function of energies *μ*(*E*) were processed by background subtraction and normalization procedures and reported as “normalized absorption” for all the measured samples and standard references. For the extended X-ray absorption fine structure (EXAFS) part, the Fourier transformed (FT) data in *R* space were analyzed by applying different models for M–O, M–Cl and M–M (M = Ir, Pd) contributions. The passive electron factors, *S*_0_^2^, were determined by fitting the experimental data on metal foils and fixing the coordination number (CN) of M–M, and then fixed for further analysis of the measured samples. The parameters describing the electronic properties (e.g., correction to the photoelectron energy origin, *E*_0_) and local structure environment including CN, bond distance (*R*), and Debye–Waller factor around the absorbing atoms could vary during the fit process.

### Photocatalytic CO_2_RR in the conventional particle-in-solution (PiS) mode

The photocatalytic CO_2_ reduction reaction (CO_2_RR) experiments were carried out in an outer irradiation-type photo-reactor (Pyrex glass) connected to a closed gas-circulation system (Supplementary Fig. 21). High-purity CO_2_ (99.9999%) was cycled by a gas-recycle-pump with tunable flow rates. Typically, 15 mg of catalyst powders were dispersed in a mixture of water and isopropanol (40 mL of water and 10 mL of isopropanol). The dispersion was transferred into the catalysis system, thoroughly degassed to remove the air by high-purity Ar (99.9999%) displacement. After 30 min of high-purity (99.9999%) CO_2_ bubbling, the dispersion was illuminated by a 300 W Xe-lamp (Beijing China Education Au-light Co., Ltd.) with a 420 nm long-pass filter. During the catalysis tests, the CO_2_ was continuously bubbled at the flow rate of 120 mL min^–1^ into the dispersion, which was magnetically stirred at 800 rpm. The liquid products (e.g., HCOOH) were quantified with NMR. Typically, after photocatalytic CO_2_RR, 500 µL of the resultant solution was extracted from the reactor and mixed with 100 µL of D_2_O (99.9 at.% D, Sigma-Aldrich) containing 0.05 μL of dimethyl sulfoxide (99.9%, Sigma-Aldrich,) as an internal standard. The ^1^H NMR spectra were then measured with water suppression using a pre-saturation method. The gaseous products were quantified by an online gas chromatography (Shimadzu GC-2014C) with a packed column (MS-13X). A thermal conductivity detector (TCD) was used to quantify H_2_ concentration, and a flame ionization detector (FID) with a methanizer was used to quantitatively analyze the content of CO.

### Photocatalytic CO_2_RR in the GMG mode

The photocatalytic CO_2_RR tests of the leaf-like membranes in the GMG mode were carried out in a home-made outer irradiation-type gas-flow cell connected to a closed gas-circulation system (Supplementary Fig. 34). The as-prepared membranes were sandwiched by two gas chambers (Supplementary Fig. 35). During the photocatalysis process, humidified high-pure CO_2_ (99.9999%) was cycled continuously in the whole system by a gas-recycle-pump with tunable flow rates, so that the CO_2_ and water molecules could continuously pass across the membranes. Before tests, the whole catalytic system was thoroughly degassed to remove the air by high-purity Ar (99.9999%) displacement. After 30 min of high-purity (99.9999%) CO_2_ circulation, the membranes were illuminated by a 300 W Xe-lamp (Beijing China Education Au-light Co., Ltd.) with a 420 nm long-pass filter. The water vapor carried liquid products (e.g., HCOOH) were collected by a cold trap and then quantified with NMR. The gas-phase products (e.g., H_2_) were quantified by an online gas chromatography (Shimadzu GC-2014C).

### Photocatalytic CO_2_RR in the spontaneous diffusion mode

The photocatalytic CO_2_RR tests of the 1.4 wt% Ir_1_/A-aUiO membranes in the spontaneous diffusion mode were carried out following previously reported procedures^25^. Before reaction, the Ir_1_/A-aUiO membrane with the effective geometric area of ~12.5 cm^2^ was placed horizontally in the center of an air-tight reaction cell (400 mL). Then, the air inside the reaction cell was evacuated before the cell was filled with ultra-pure CO_2_. This vacuum and refill procedure was repeated three times to remove the air residuals in the reactor. After another 30 min for the establishing of an adsorption–desorption balance, 3 mL of ultrapure H_2_O was added to the reactor (without direct contact with the membrane) to provide humidity. A 300 W Xe lamp with a 420 nm long-pass filter was employed as a light source to illuminate the catalyst membrane from the top of the reactor. After 2 h of irradiation, the reaction cell was further heated to 60 °C to ensure the generated HCOOH was fully gasified, the water vapor carried HCOOH were then collected by a cold trap and quantified with NMR.

### Photocatalytic O_2_ reduction in the conventional particle-in-solution (PiS) mode

The photocatalysis O_2_ reduction reaction (ORR) tests were also carried out in an outer irradiation-type photoreactor (Pyrex glass) connected to a closed gas-circulation system. High-purity O_2_ (99.9999%) was cycled by a gas-recycle-pump with tunable flow rates. Typically, 20 mg of the photocatalyst powders were dispersed in a mixture of water (40 mL) and isopropanol (10 mL). The dispersion was transferred into the catalysis system, thoroughly degassed to remove the air by high-purity Ar (99.9999%) displacement. After 30 min of high-purity (99.9999%) O_2_ bubbling, the dispersion was illuminated by a 300 W Xe-lamp (Beijing China Education Au-light Co., Ltd.) with a 420 nm long-pass filter. During the photocatalytic tests, the O_2_ was continuously bubbled at the flow rate of 120 mL min^–1^ into the dispersion, which was magnetically stirred at 800 rpm. The H_2_O_2_ decomposition reactions were carried out in a Pyrex glass vial. Twenty milligram of the catalyst powders were dispersed in a mixture of hydrogen peroxide (50 µmol), water (40 mL) and isopropanol (10 mL). The dispersion was transferred into the catalysis system, thoroughly degassed to remove the air by high-purity Ar (99.9999%) displacement. After that, the catalysis reactions were carried out under visible light irradiation. During the tests, the Ar was continuously bubbled at the flow rate of 30 mL min^–1^ into the dispersion, which was magnetically stirred at 800 rpm. The H_2_O_2_ concentration was measured by a cerium sulfate Ce(SO_4_)_2_ titration method based on the mechanism, that the yellow solution of Ce^4+^ could be reduced to colorless Ce^3+^ by H_2_O_2_ according to the following equation:

1 $$2{{\rm{Ce}}}^{4+}+{{\rm{H}}}_{2}{{\rm{O}}}_{2}\to 2{{\rm{Ce}}}^{3+}+{{\rm{O}}}_{2}+2{{\rm{H}}}^{+}$$

Thus, the concentration of H_2_O_2_ could be obtained by measuring the concentration of Ce^4+^ (UV–Visible absorption at the wavelength of 316 nm) ref. ^48^.

### Photocatalytic O_2_ reduction in the GMG mode

The photocatalytic O_2_ reduction tests of the leaf-like membranes in the GMG mode were also carried out in a home-made outer irradiation-type gas-flow cell connected to a closed gas-circulation system. The as-prepared membranes were sandwiched by two gas chambers. During the catalysis process, humidified high-purity O_2_ (99.9999%) was cycled continuously in the whole system by a gas-recycle-pump with tunable flow rates, so that the O_2_ and water molecules could continuously pass across the membranes. In addition, the surfaces of the leaf-like membranes needed to be washed by ultrapure water (was cycled by a peristaltic pump) intermittently (with a fixed interval of 20 min) to ensure the efficient collection of the liquid H_2_O_2_ generated during the catalytic process. Before tests, the whole catalytic system was thoroughly degassed to remove the air by high-purity Ar (99.9999%) displacement. After 30 min of high-purity (99.9999%) O_2_ circulation, the membranes were illuminated by a 300 W Xe-lamp (Beijing China Education Au-light Co., Ltd.) with a 420 nm long-pass filter.

### AQE for formate production at different wavelength

The calculation of the AQE followed the standard procedure of photocatalytic reactions. In detail, the AQE was obtained by performing CO_2_RR under monochromatic light using appropriate bandpass filters at different wavelengths (e.g., 380, 400, 420, 450, 475, 500, 520, 550, 575, and 600 nm). The power intensity of the monochromatic light was measured using an optical power meter (CEL-NP2000, Beijing China Education Au-light Company Limited, China). The calculation of AQE was based on:

2 $${\text{AQE}}=\frac{{N}_{{\rm{reacted}}}}{{N}_{{\rm{incident}}}}\times 100 \%$$

In the above equation, *N*_reacted_ and *N*_incident_ are the number of the reacted and incident photons, respectively, which can be obtained from:

3 $${N}_{{\rm{reacted}}}\,=\,2\times {N}_{{\rm{A}}}\times {n}_{{\rm{HCOOH}}},$$

4 $${N}_{{\rm{incident}}}\,=\,\frac{{Pt}}{h\upsilon }\,=\,\frac{{ISt}\lambda }{{hc}},$$

where *N*_A_ is the Avogadro constant (6.022 × 10^23^ mol^–1^), *n*_HCOOH_ is the amount of the produced HCOOH molecules (in mol), *P* is the light power (W), *t* is the illumination time (s), *h* is the Planck constant (6.626 × 10^–34^ J s), *ν* is light frequency (Hz), *I* is the light intensity (W cm^–2^), *S* is the irradiated area (cm^2^), *λ* is wavelength of monochromatic light (m), and *c* is the speed of light in free space (3.0 × 10^8^ m s^–1^).

### Photo-electrochemical measurements

Photo-electrochemical tests were performed using a CHI 660E potentiostat with a three-electrode system. A platinum plate (1 × 1 cm^2^), a saturated calomel electrode (SCE), and a modified fluorine-doped tin oxide (FTO) glass plate (1 × 2 cm^2^) were used as the counter, reference, and working electrode, respectively. All potentials in this study were measured against the SCE and converted to the reversible hydrogen electrode (RHE) reference scale by

5 $$E({\rm{V}}\,{\rm{vs.}}\,{\rm{RHE}})\,=\,E({\rm{V}}\,{\rm{vs.}}\,{\rm{SCE}})\,+\,0.0591\times {\rm{pH}}\,+\,0.244$$

To prepare working electrodes, 15 mg of catalyst powders were dispersed in the mixed solution of 2 mL of water, 0.7 mL of ethanol and 0.3 mL of 5 wt% Nafion by sonication for at least 30 min to form a homogeneous ink. Then, 150 μL of the as-prepared catalyst inks were loaded onto the FTO electrode and dried under room temperature.

## Supplementary information

Supplementary Information

Peer Review

## Data Availability

All data that support the plots and other findings within this paper are available from the corresponding authors on reasonable request. Source data are provided with this paper.

## Source data

Source Data
