# Supplementary material for: Metal-organic framework membranes with single-atomic centers for photocatalytic CO2 and O2 reduction
Source: Nat Commun. 2021 May 11;12:2682. doi: 10.1038/s41467-021-22991-7 (PMC8113524; doi:10.1038/s41467-021-22991-7)
Supplement: Supplementary file 1 — Supplementary Information [file 41467_2021_22991_MOESM1_ESM.pdf]

Supplementary Materials for

Metal-organic framework membranes with single-atomic centers for photocatalytic CO_2 and O_2 reduction

Yu-Chen Hao[1], Li-Wei Chen[1], Jiani Li[1], Yu Guo[2], Xin Su[1], Miao Shu[3], Qinghua Zhang[4], Wen-Yan Gao[1], Siwu Li[1], Zi-Long Yu[1], Lin Gu[4], Xiao Feng[1], An-Xiang Yin[1]*, Rui Si[3]*, Ya-Wen Zhang[2], Bo Wang[1,5]* and Chun-Hua Yan[2]

[1] *Ministry of Education Key Laboratory of Cluster Science, Beijing Key Laboratory of Photoelectronic/Electrophotonic Conversion Materials, School of Chemistry and Chemical Engineering, Beijing Institute of Technology, Beijing 100081, P. R. China.*

[2] *Beijing National Laboratory for Molecular Sciences, State Key Laboratory of Rare Earth Materials Chemistry and Applications, PKU-HKU Joint Laboratory in Rare Earth Materials and Bioinorganic Chemistry, College of Chemistry and Molecular Engineering, Peking University, Beijing 100871, P. R. China*

[3] *Shanghai Synchrotron Radiation Facility, Shanghai Institute of Applied Physics, Chinese Academy of Sciences, Shanghai 201204, P. R. China*

[4] *Institute of Physics, Chinese Academy of Sciences, Beijing 100190, P. R. China*

[5] *Advanced Technology Research Institute (Jinan), Beijing Institute of Technology, Jinan 250300, P. R. China*

* To whom correspondence should be addressed: yin@bit.edu.cn (A.X.Y.); sirui@sinap.ac.cn (R.S.); bowang@bit.edu.cn (B.W.)

Supplementary Data

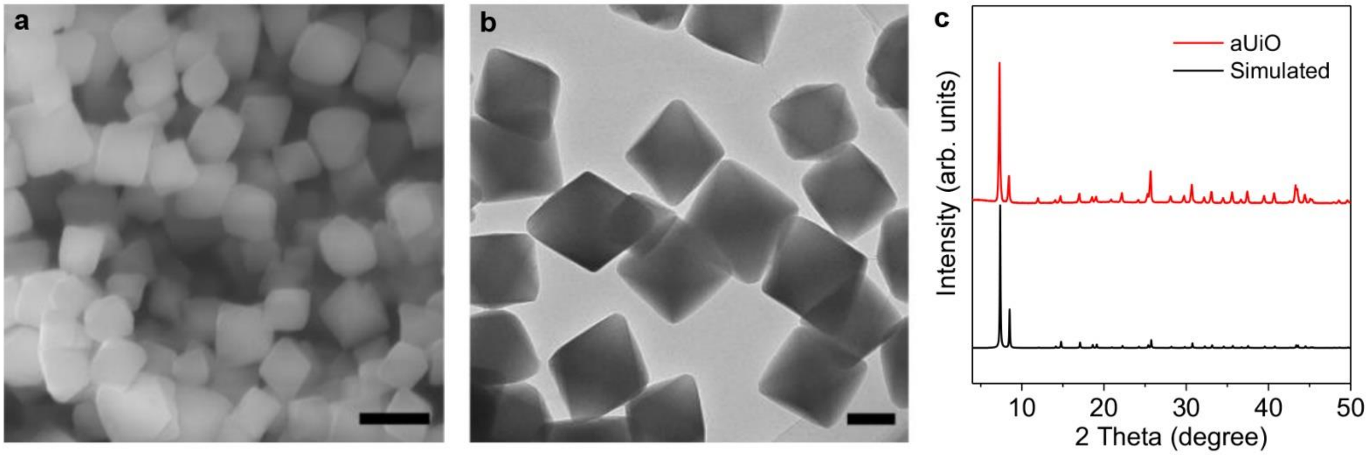

Supplementary Figure 1. Characterization of aUiO particles. (**a**) SEM image, (**b**) TEM image and (**c**) XRD pattern for aUiO. Scale bar: (**a**) 500 nm and (**b**) 200 nm.

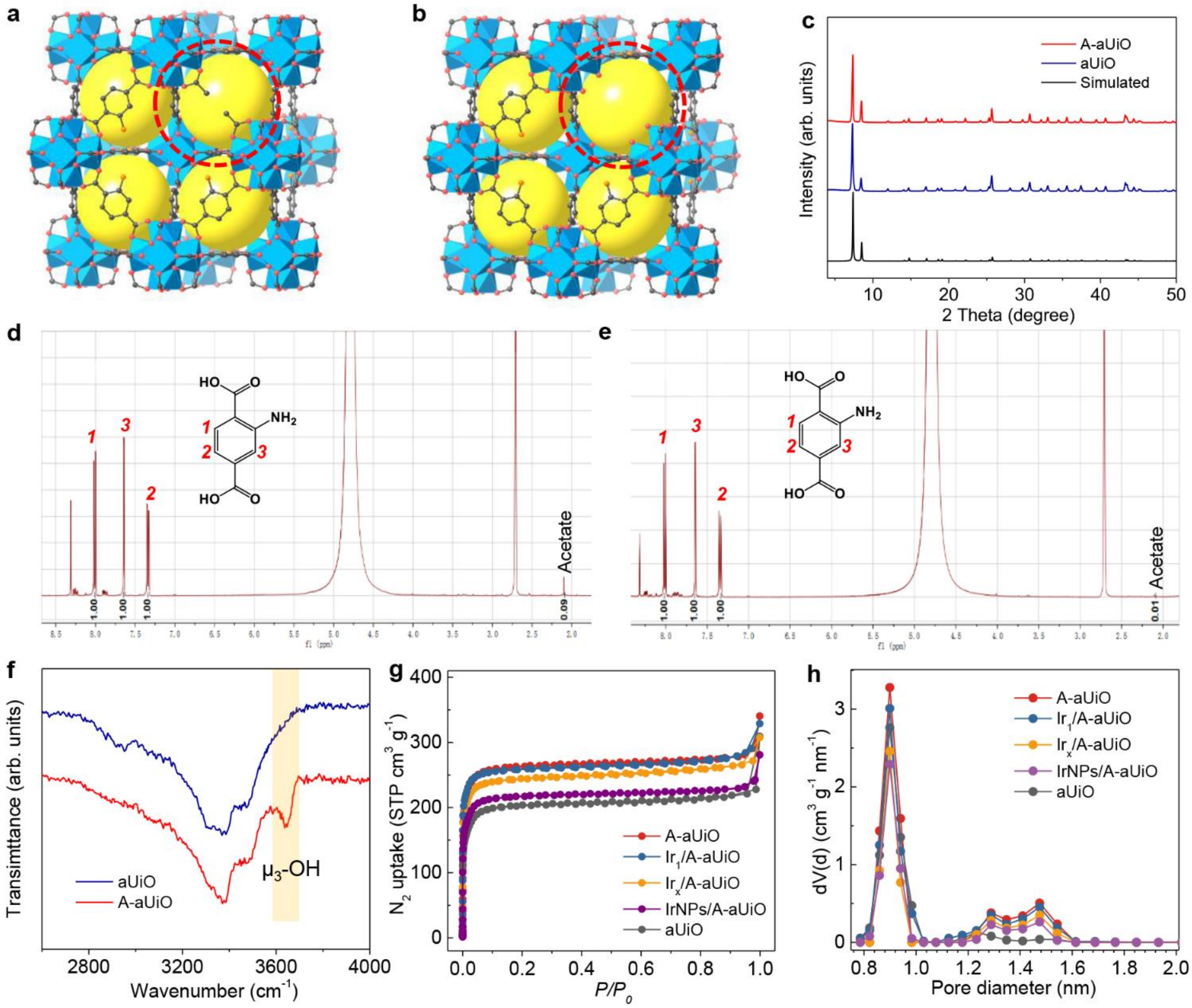

Supplementary Figure 2. Preparation and characterization of aUiO and A-aUiO particles. (**a**, **b**) Crystal structures of (a) aUiO and (b) A-aUiO. The ligands (2-aminoterephthalic acid) of aUiO were partially replaced by acetates (highlighted by the red circle), which were removed by activating the aUiO powders under high vacuum and temperature to create defects. (**c**) XRD patterns for A-aUiO (red) and aUiO (blue), suggesting that they shared the same crystal structures before/after the removal of acetates. (**d**, **e**) ^{1}H NMR profiles of digested aUiO (d) and A-aUiO (e) particles, showing the composition of organic residuals of them. Before ^{1}H NMR test, the samples (~5 mg) were digested and dissolved in a mixture solution of DMSO-d_6 (560 μL), hydrofluoric acid (48%, 20 μL) and D_2O (20 μL). After high-temperature activation, the molar ratio of acetate/2-aminoterephthalic acid was decreased from 0.03 (aUiO) to 0.0033 (A-aUiO), suggesting the effective removal of acetate residuals from the aUiO matrix. (**f**) FT-IR spectra of A-aUiO (red) and aUiO (blue). The presence of μ_3–OH vibration in the post-activation A-aUiO samples suggests the effective removal of coordinated acetates from the Zr_6–O clusters, resulting in A-aUiO particles with missing linkers and Zr_6–O clusters with abundant defects terminated by −OH/−OH_2 groups[1]. (**g, h**) N_2 adsorption-desorption isotherms (g) and pore size distributions (h) of aUiO, A-aUiO, Ir_1/A-aUiO (1.4 wt.%), Ir_x/A-aUiO (2.7 wt.%) and IrNPs/A-aUiO (4.0 wt.%) samples, showing the creating of external micropores upon the removal of acetates.

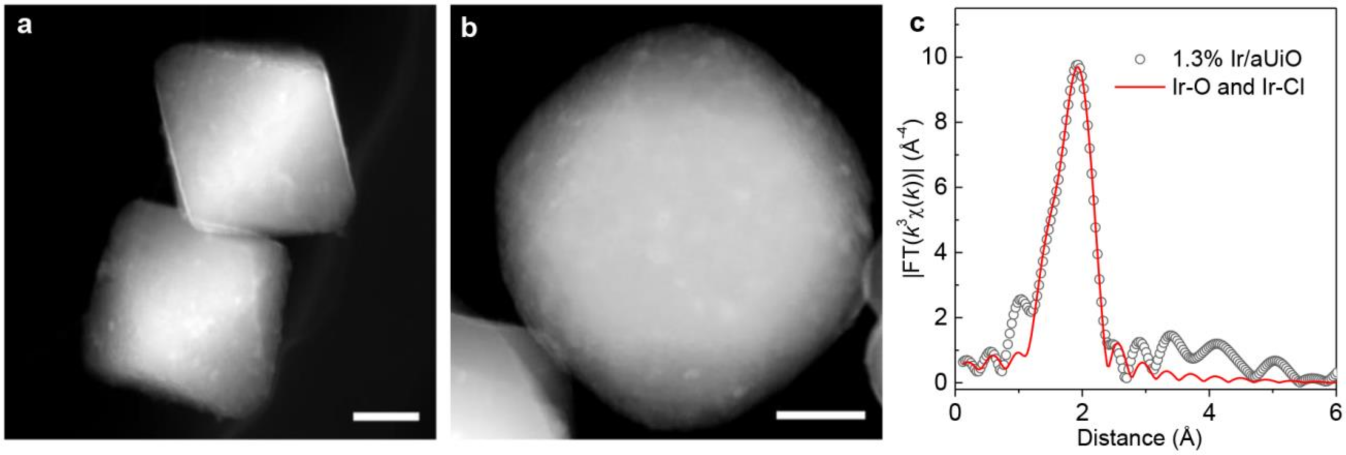

Supplementary Figure 3. STEM and EXAFS characterization of the as-prepared defect-deficient Ir/aUiO samples. (**a, b**) HAADF-STEM images of Ir/aUiO particles showing the existence of small nanoparticles in the aUiO matrix. Scale bar: 100 nm. (**c**) Ir L_3-edge EXAFS fitting results for Ir/aUiO (1.3 wt.%), revealing the presence of both Ir–O (coordination number: 2.4) and Ir–Cl (coordination number: 3.8) coordination in the as-prepared Ir/aUiO.

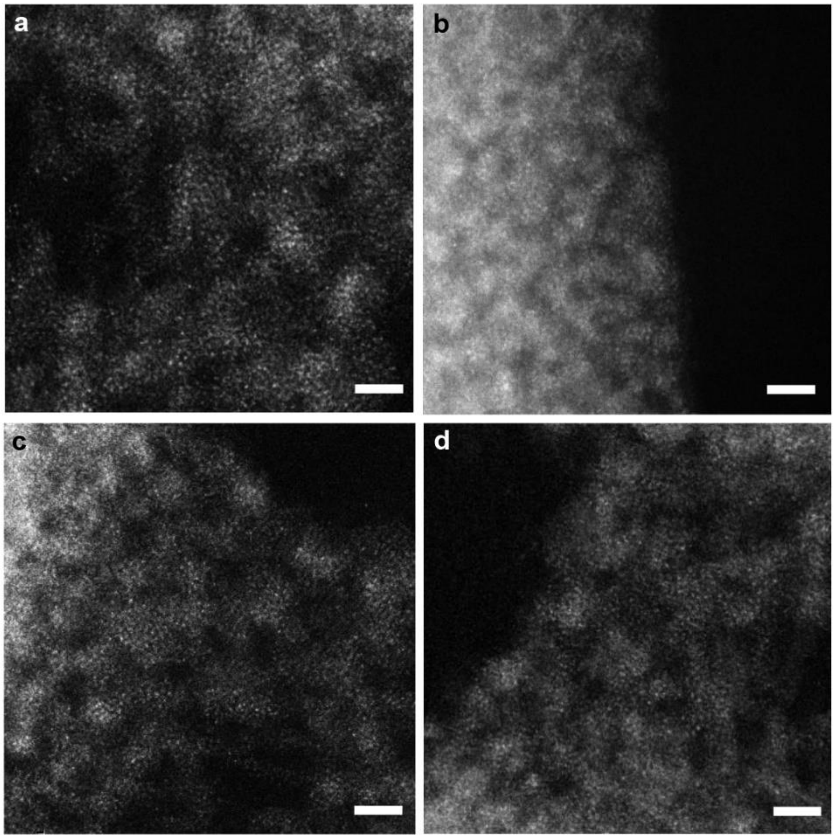

Supplementary Figure 4. High-resolution aberration-corrected HAADF-STEM images of Ir_1/A-aUiO particles (1.4 wt.%). Scale bar: 2 nm.

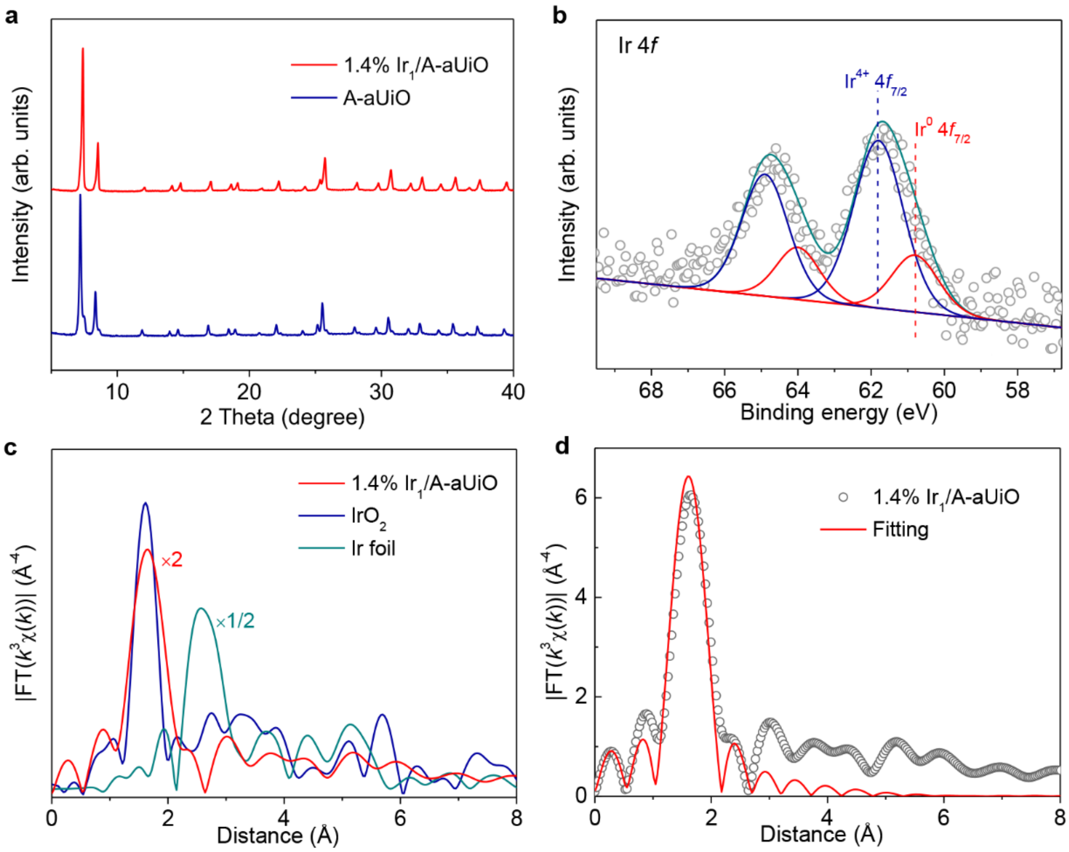

Supplementary Figure 5. XRD, XPS and EXAFS characterization of the as-prepared Ir_1/A-aUiO (1.4 wt.%) samples. (**a**) XRD patterns for A-aUiO (blue) and Ir_1/A-aUiO (red) particles. (**b**) Ir 4*f* core level XPS spectrum for Ir_1/A-aUiO showing that most Ir species were in highly oxidized state. (**c**) Ir L_3-edge EXAFS spectra of Ir_1/A-aUiO (red), IrO_2 (blue) and bulk Ir foil (cyan). (**d**) Ir L-edge EXAFS fitting results (CN of Ir–O: 3.8) for Ir_1/A-aUiO. The presence of Ir–O coordination but no Ir–Ir signals confirmed the atomic dispersion of Ir species in the 1.4 wt.% Ir_1/A-aUiO samples.

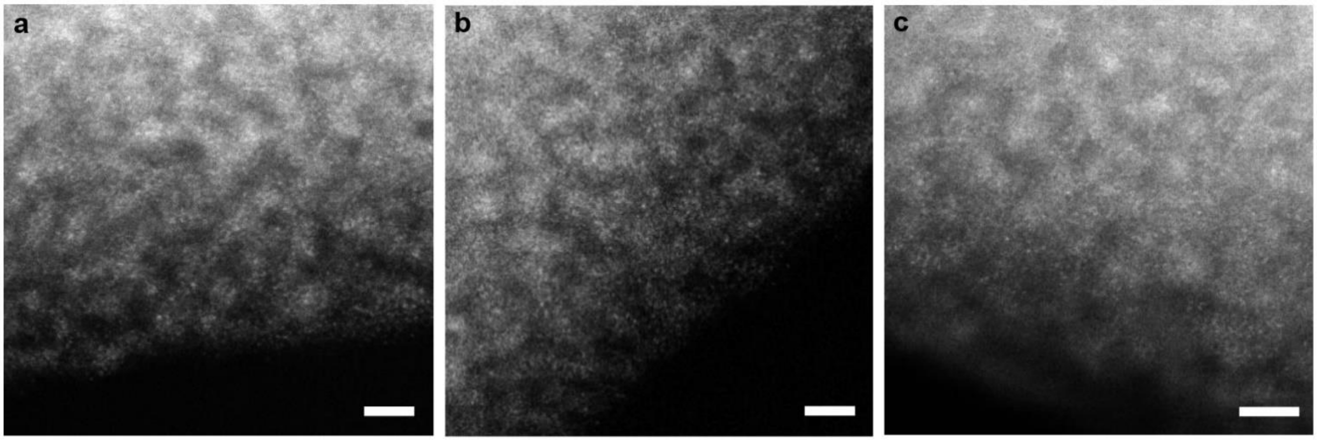

Supplementary Figure 6. High-resolution aberration-corrected HAADF-STEM images of 0.7 wt.% Ir_1/A-aUiO particles. Scale bar: 2 nm.

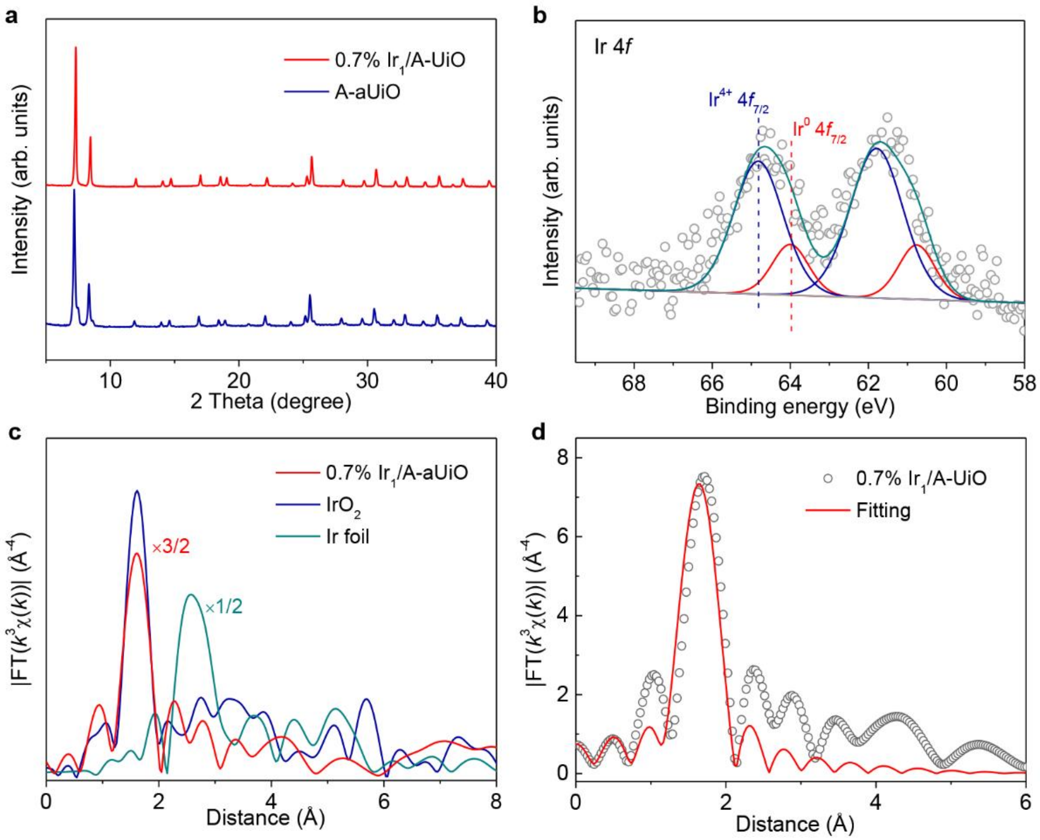

Supplementary Figure 7. XRD, XPS and EXAFS characterization of the as-prepared 0.7 wt.% Ir_1/A-aUiO samples. (**a**) XRD patterns for A-aUiO (blue) and 0.7 wt.% Ir_1/A-aUiO (red). (**b**) Ir 4*f* core level XPS spectrum for 0.7 wt.% Ir_1/A-aUiO. (**c**) Ir L_3-edge EXAFS spectra of 0.7 wt.% Ir_1/A-aUiO (red), IrO_2 (blue) and bulk Ir foil (cyan). (**d**) Ir L_3-edge EXAFS fitting results (CN of Ir–O: 4.0) for 0.7 wt.% Ir_1/A-aUiO. The presence of Ir–O coordination but no Ir–Ir signals confirmed the atomic dispersion of Ir species in 0.7% Ir_1/A-aUiO.

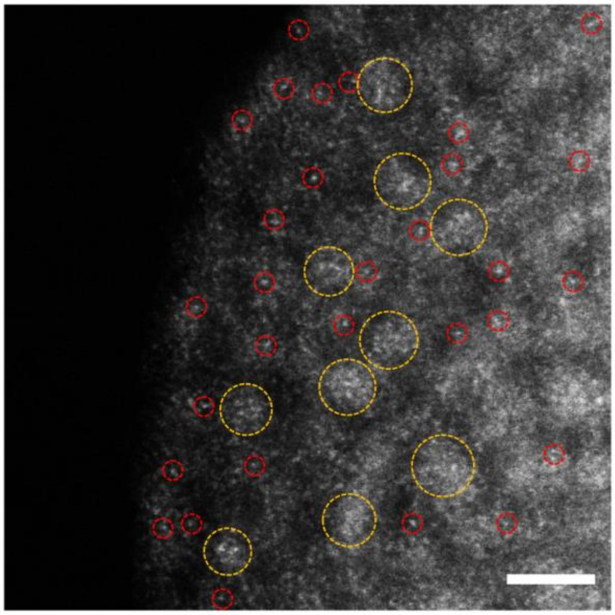

Supplementary Figure 8. High-resolution aberration-corrected HAADF-STEM image of the as-prepared Ir_x/A-aUiO (2.7 wt.%) samples. Both atomically dispersed Ir (highlighted by red circles) and Ir clusters (highlighted by yellow circles) can be found in the Ir_x/A-aUiO samples. Scale bar: 2 nm.

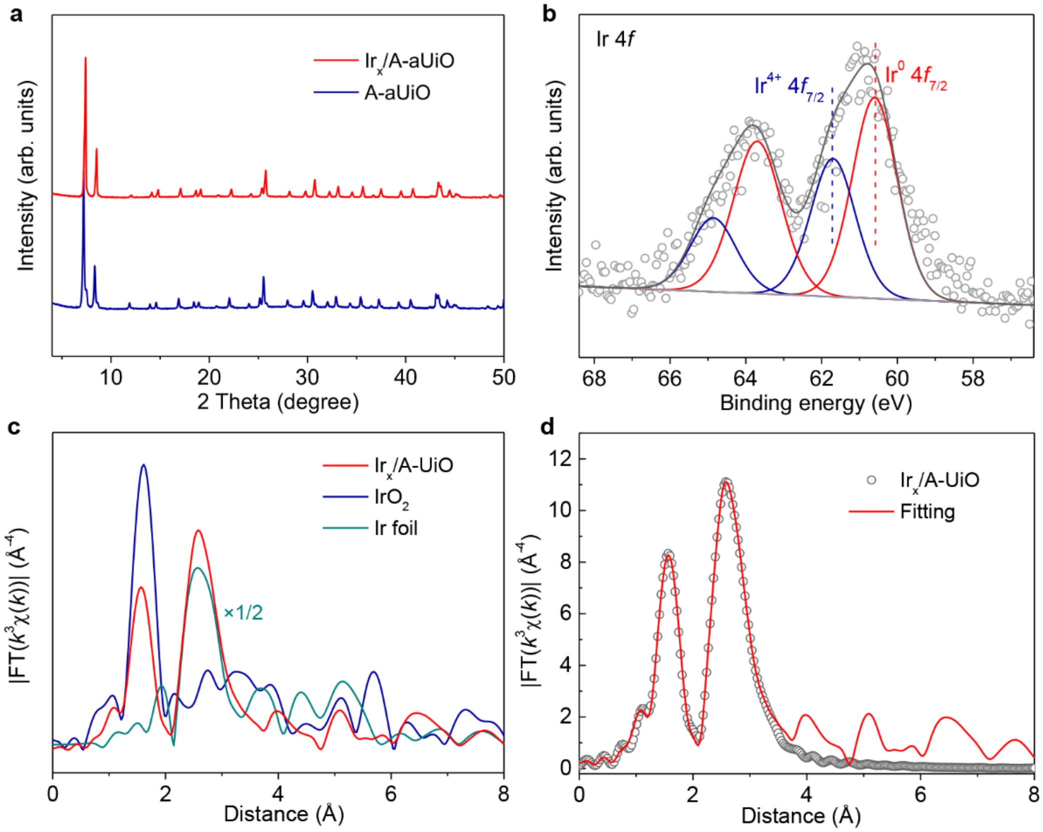

Supplementary Figure 9. XRD, XPS and EXAFS characterization of as-prepared Ir_x/A-aUiO (2.7 wt.%) samples. (**a**) XRD patterns for A-aUiO (blue) and Ir_x/A-aUiO (red), respectively. (**b**) Ir 4*f* core level XPS spectrum for Ir_x/A-aUiO showing more Ir species were in lower oxidation states. (**c**) Ir L_3-edge EXAFS spectra of Ir_x/A-aUiO (red), IrO_2 (blue) and bulk Ir foil (cyan). (**d**) Ir L_3-edge EXAFS fitting results for Ir_x/A-aUiO. The two notable peaks in the region of 1 to 3 Å represent the Ir–O and Ir–Ir contribution, respectively.

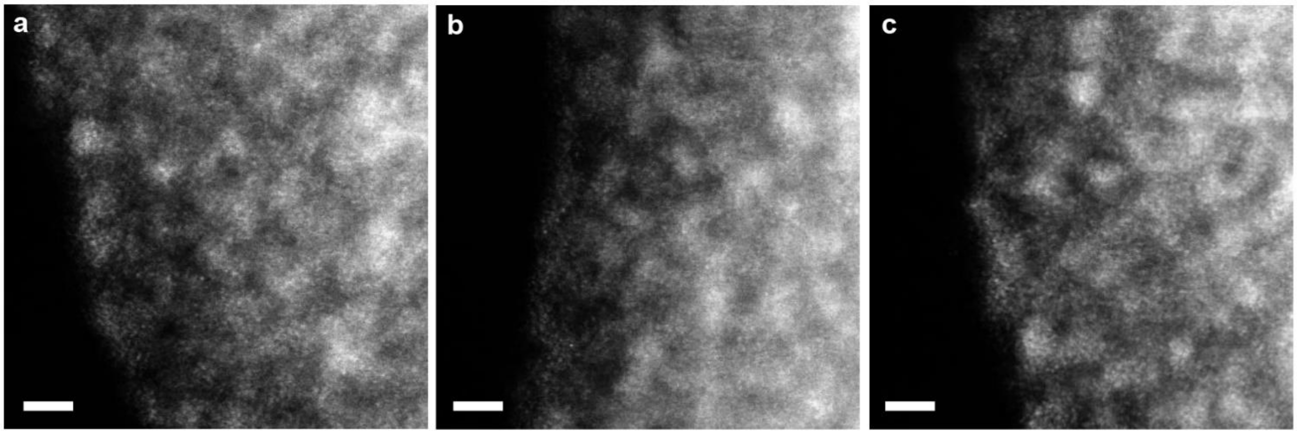

Supplementary Figure 10. High-resolution aberration-corrected HAADF-STEM images of the as-prepared Pd_1/A-aUiO **(0.8 wt.%) samples.** Scale bar: 2 nm.

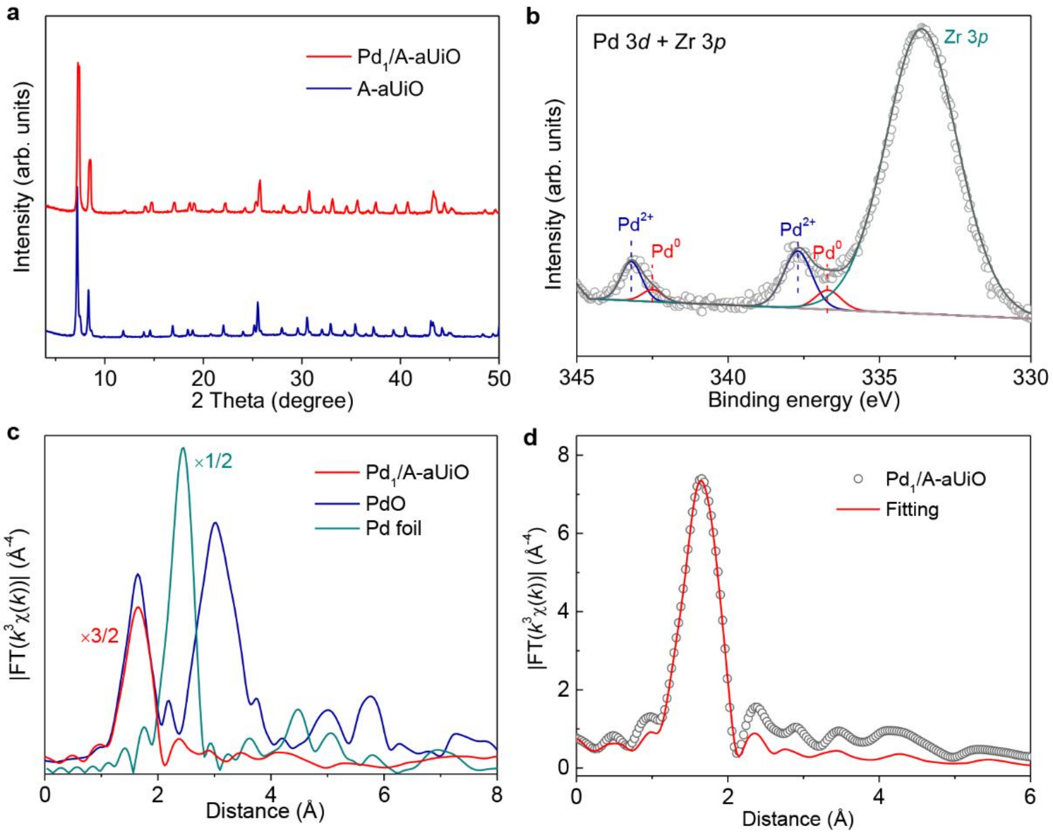

Supplementary Figure 11. XRD, XPS and EXAFS characterization of the as-prepared Pd_1/A-aUiO (0.8 wt.%) samples. (**a**) XRD patterns for A-aUiO (blue) and Pd_1/A-aUiO (red). (**b**) Pd 3*d* and Zr 3*p* core level XPS spectra for Pd_1/A-aUiO showing most Pd species were in oxidized states. (**c**) Pd K-edge EXAFS spectra of Pd_1/A-aUiO (red), PdO (blue) and bulk Pd foil (cyan). (**d**) Pd K-edge EXAFS fitting results for Pd_1/A-aUiO. The presence of only one notable peak in the region of 1 to 2 Å indicates the existence of only Pd–O coordination with no Pd–Pd contribution (in the region of 2 to 3 Å), confirming the atomic dispersion of Pd species in the Pd_1/A-aUiO samples.

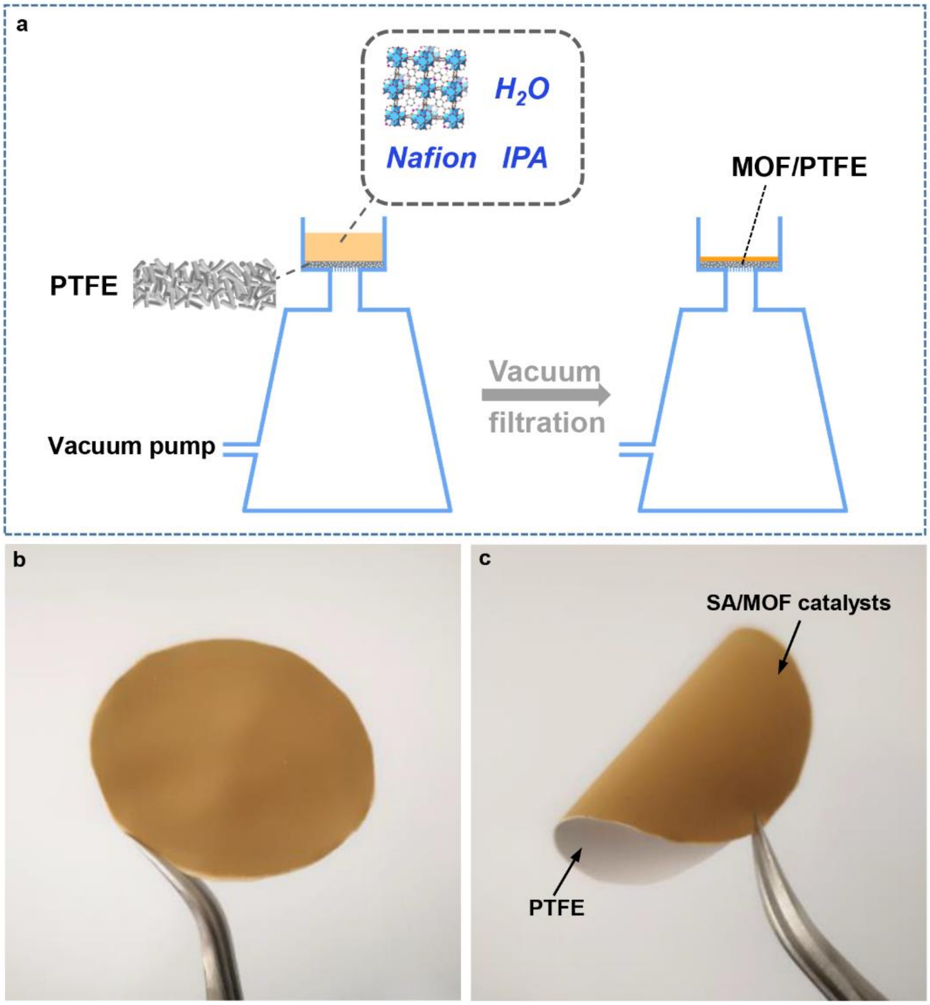

Supplementary Figure 12. Fabrication strategy for the leaf-like SA/MOF membrane. (**a**) Illustration for the preparation of PTFE-supported SA/MOF membranes via the vacuum filtration method. (**b, c**) Digital photographs of the as-prepared PTFE-supported SA/MOF (e.g. Ir_1/A-aUiO) membrane, revealing the high flexibility of the leaf-like membrane.

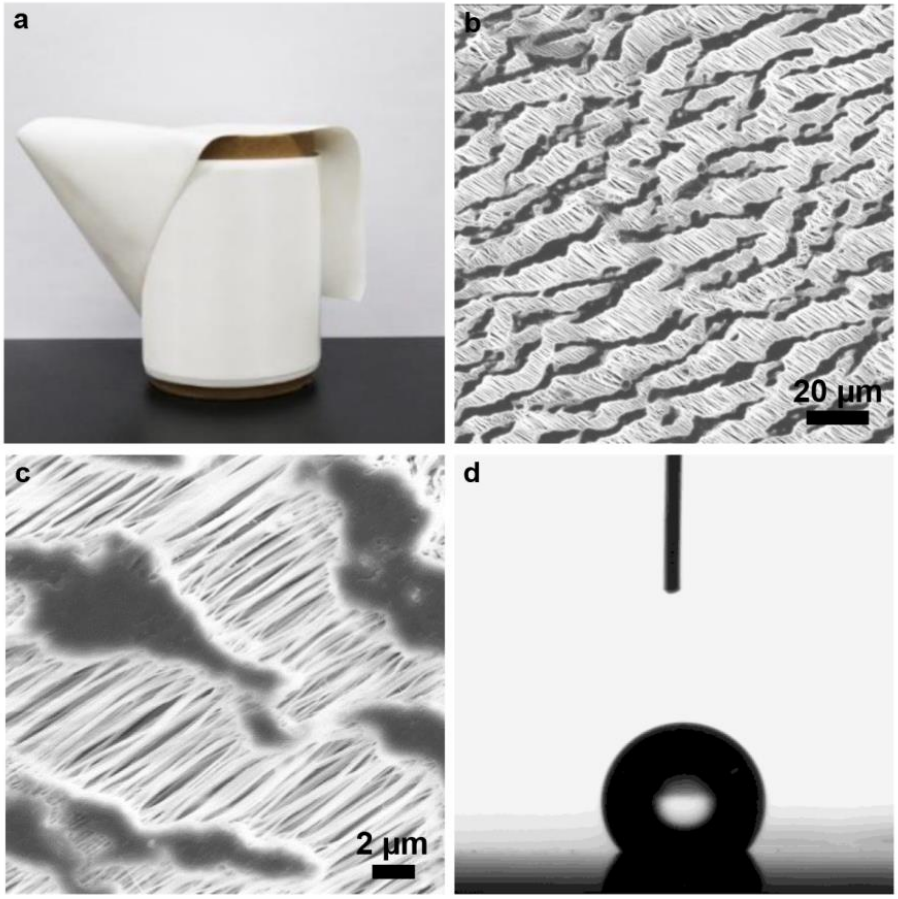

Supplementary Figure 13. Characterization of the porous PTFE films. (**a**) Digital photograph and (**b, c**) SEM images of the porous PTFE films. (**d**) Digital photograph of the contact angle showing the hydrophobic nature of the PTFE film.

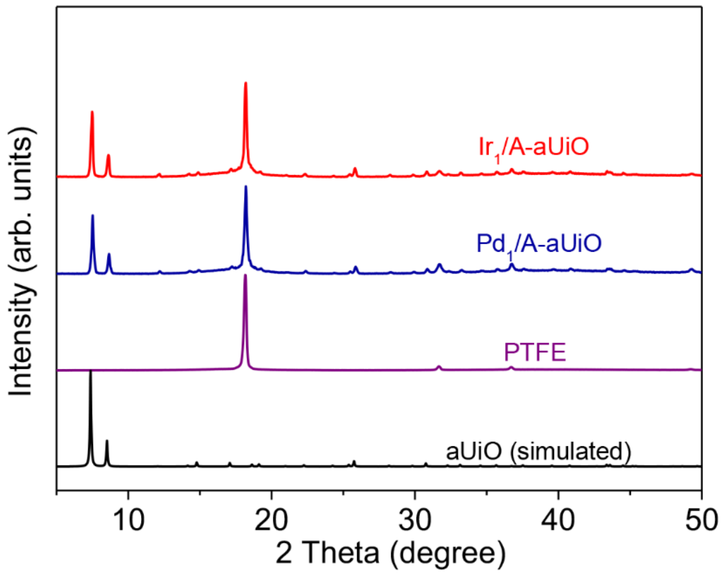

Supplementary Figure 14. XRD patterns for the PTFE film and the PTFE-supported SA/MOF membranes. Ir_1/A-aUiO (red), Pd_1/A-aUiO (blue), PTFE (violet) and simulated aUiO (black).

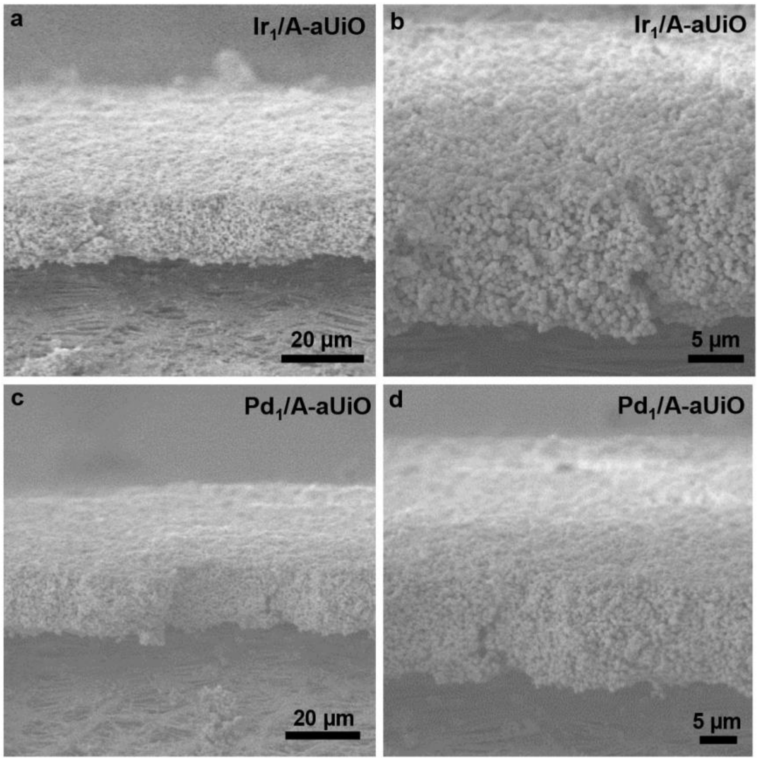

Supplementary Figure 15. Cross-sectional SEM images of the as-prepared PTFE-supported membranes. (**a**, **b**) Ir_1/A-aUiO, (**c**, **d**) Pd_1/A-aUiO.

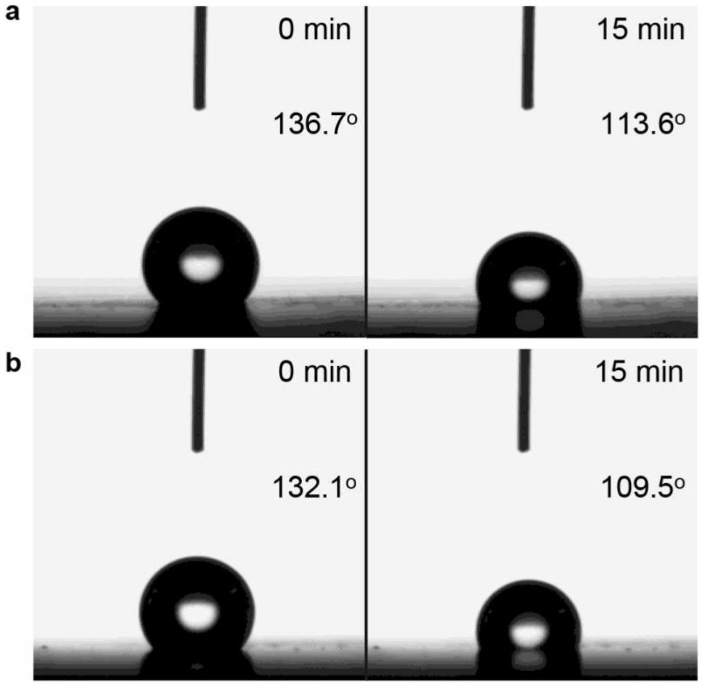

Supplementary Figure 16. Digital photographs of the contact angles on the as-fabricated membranes at 0 and 15 min. (**a**) Ir_1/A-aUiO, (**b**) Pd_1/A-aUiO. The decrease of contact angles on the SA/MOF membranes after 15 min standing suggested that the pores of the breathable SA/MOF membranes could be slightly soaked by water.

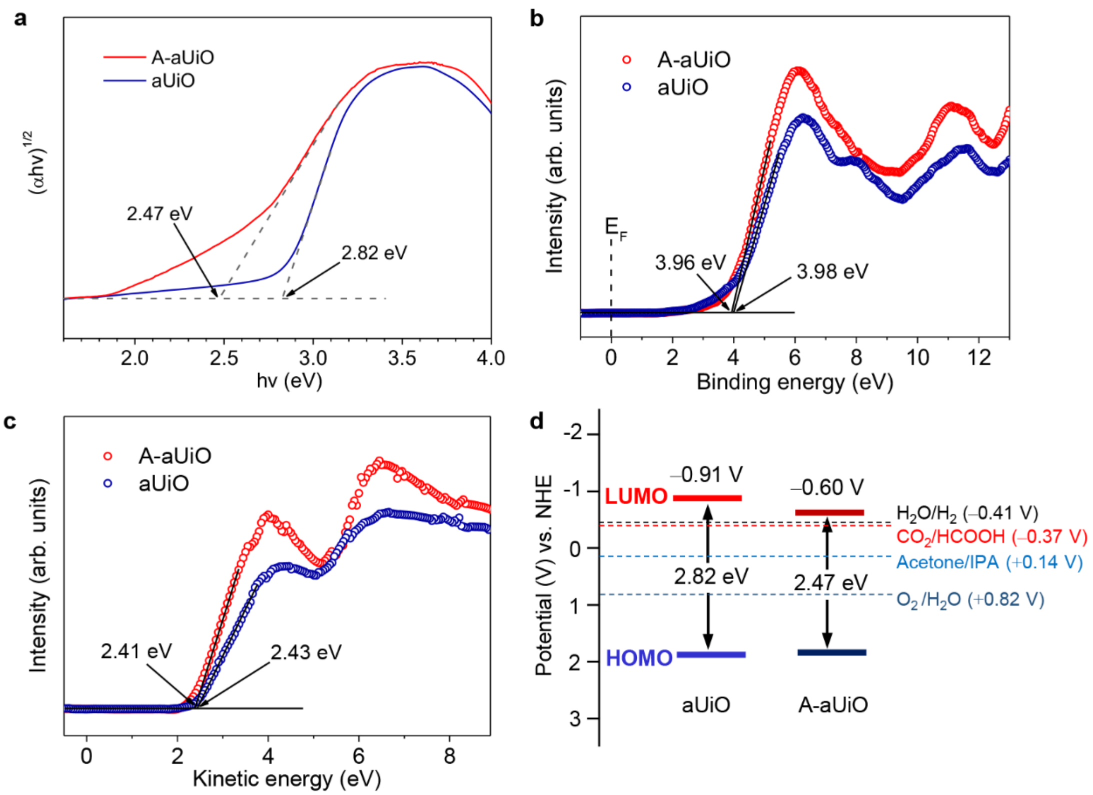

Supplementary Figure 17. Electronic band structures of aUiO samples before and after activation. (**a**) Optical band gaps determined by UV-vis diffuse reflectance, where α and ν represent the absorbance and wavenumber, respectively [2]. (**b**) Valence-band spectra for A-aUiO and aUiO measured by ultraviolet photoelectron spectroscopy (UPS)[ref.3]. Au serves as a reference for E_F set to 0 eV. (**c**) Secondary electron cutoff (E_{cutoff}) in the UPS spectra, from which the work function (Φ) can be calculated. (**d**) Schematic diagrams for conduction bands and band gap energies of aUiO and A-aUiO, and the standard electrode potentials for CO_2/HCOOH, H_2O/H_2, acetone/IPA and O_2/H_2O (vs the NHE at pH = 7)[ref.4].

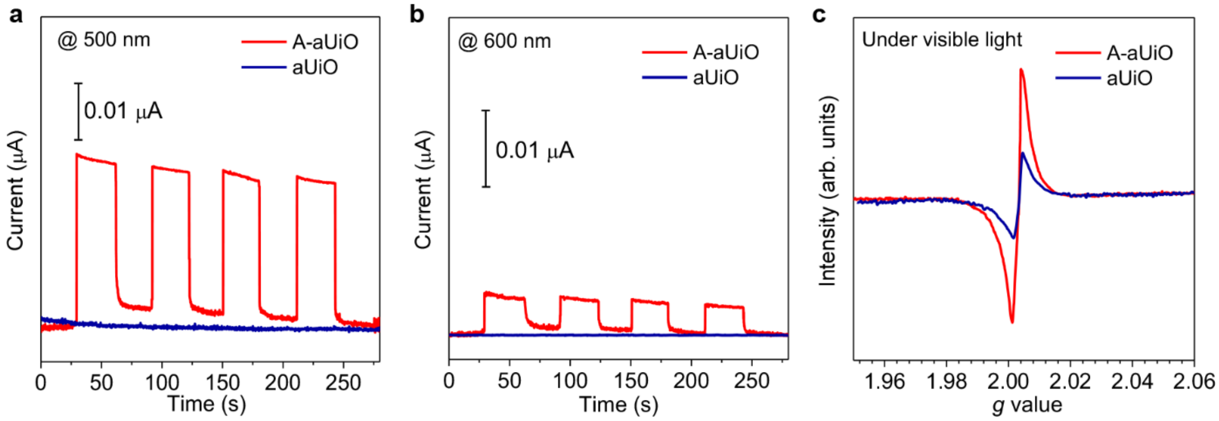

Supplementary Figure 18. Comparison of the photon-to-electron conversion efficiency of aUiO and A-aUiO samples. (**a**, **b**) Photocurrent-time curves of aUiO (blue) and A-aUiO (red) samples under monochromatic light with the wavelength of 500 nm (a) and 600 nm (b), showing the effective photo-to-electron conversion of A-aUiO as compared to aUiO. The photoelectrodes were irradiated by a 300-W Xe lamp with the bandpass filters at (500 ± 20) and (600 ± 20) nm. (**c**) Electron spin resonance (ESR) profiles of aUiO (blue) and A-aUiO (red) samples under Ar atmosphere. A much stronger ESR signal was observed on the A-aUiO samples, revealing their much higher photon-to-electron conversion efficiency as compared to aUiO.

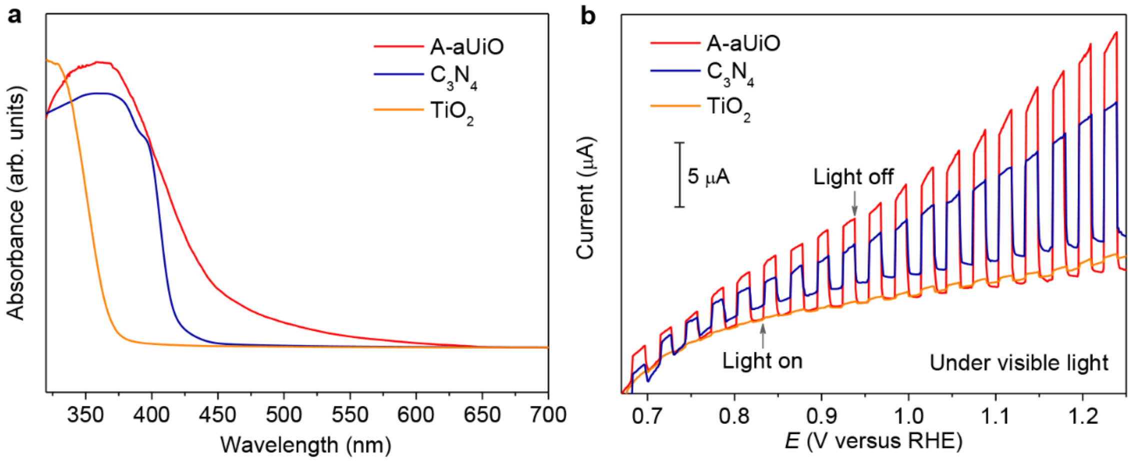

Supplementary Figure 19. Comparison of the photon-to-electron conversion efficiency of A-aUiO and other commercially available photosensitizers. (**a**) UV-vis absorption spectra and (**b**) photocurrent-potential curves for A-aUiO, C_3N_4 and TiO_2, respectively. The TiO_2 and C_3N_4 powders were purchased from Alfa Aesar (USA) and Energy Chemical (China), respectively.

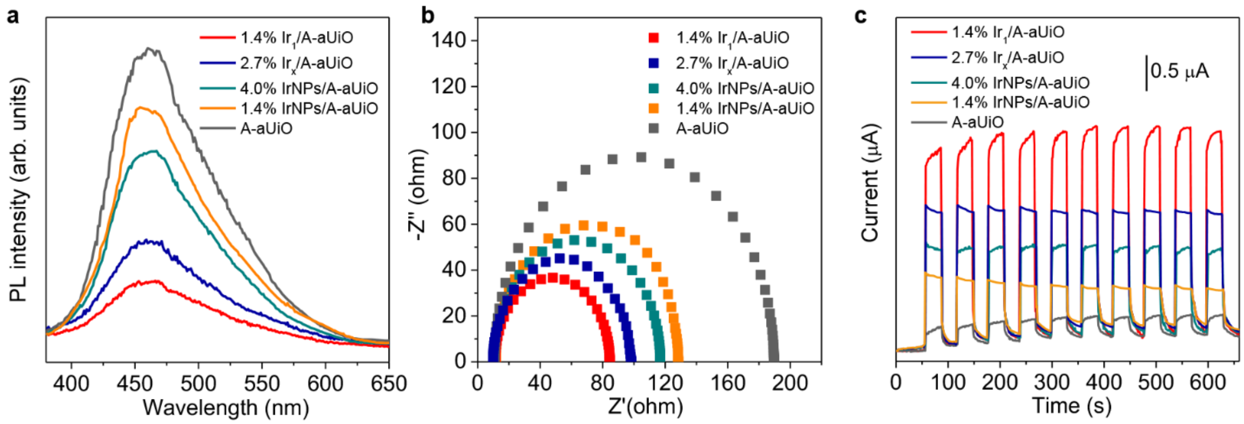

Supplementary Figure 20. Characterizations of the Ir/A-aUiO samples showing the suppression of photogenerated carrier recombination by Ir species. (**a**–**c**) Steady-state photoluminescence (PL) spectra (a), electrochemical impedance spectroscopy (EIS) (b) and photocurrent-time curves under visible light irradiation (c) for A-aUiO, 4.0 wt.% IrNPs/A-aUiO, 1.4 wt.% IrNPs/A-aUiO, 2.7 wt.% Ir_x/A-aUiO and 1.4 wt.% Ir_1/A-aUiO samples. The decoration of Ir species (*esp.* Ir SAs) could effectively boost the separation of photoexcited electron-hole pairs in A-aUiO.

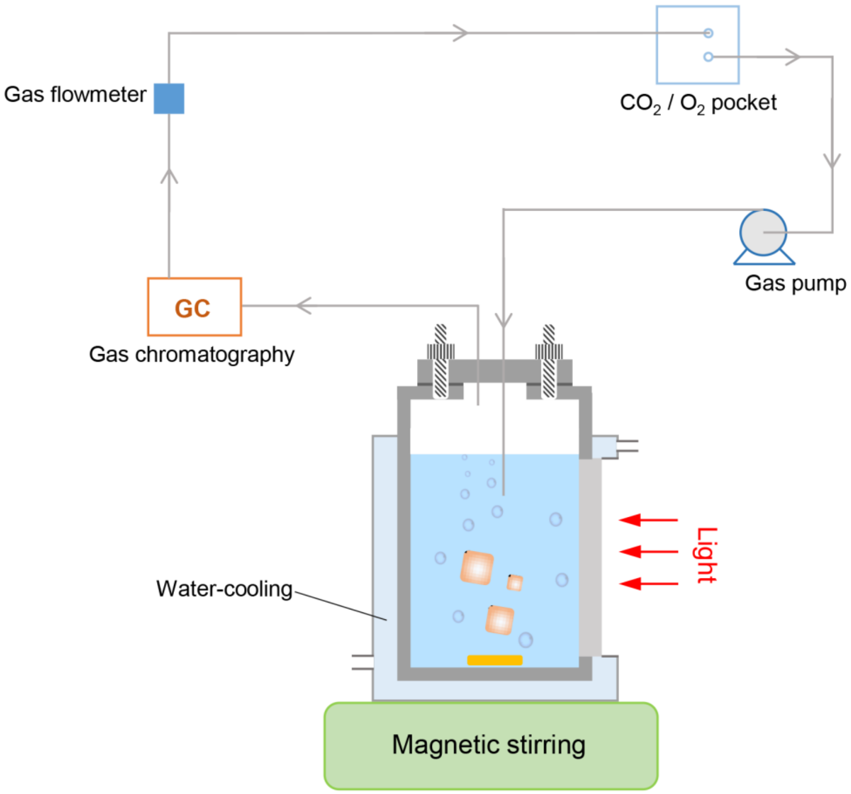

Supplementary Figure 21. Schematic for the setup of photocatalytic CO_2RR and ORR in the conventional particle-in-solution (PiS) mode. A gas-tight circulation system with on-line gas chromatography was used to quantify the gas-product (H_2). The liquid product was quantified by NMR (HCOOH) or $Ce(SO_4)_2$ titration (H_2O_2).

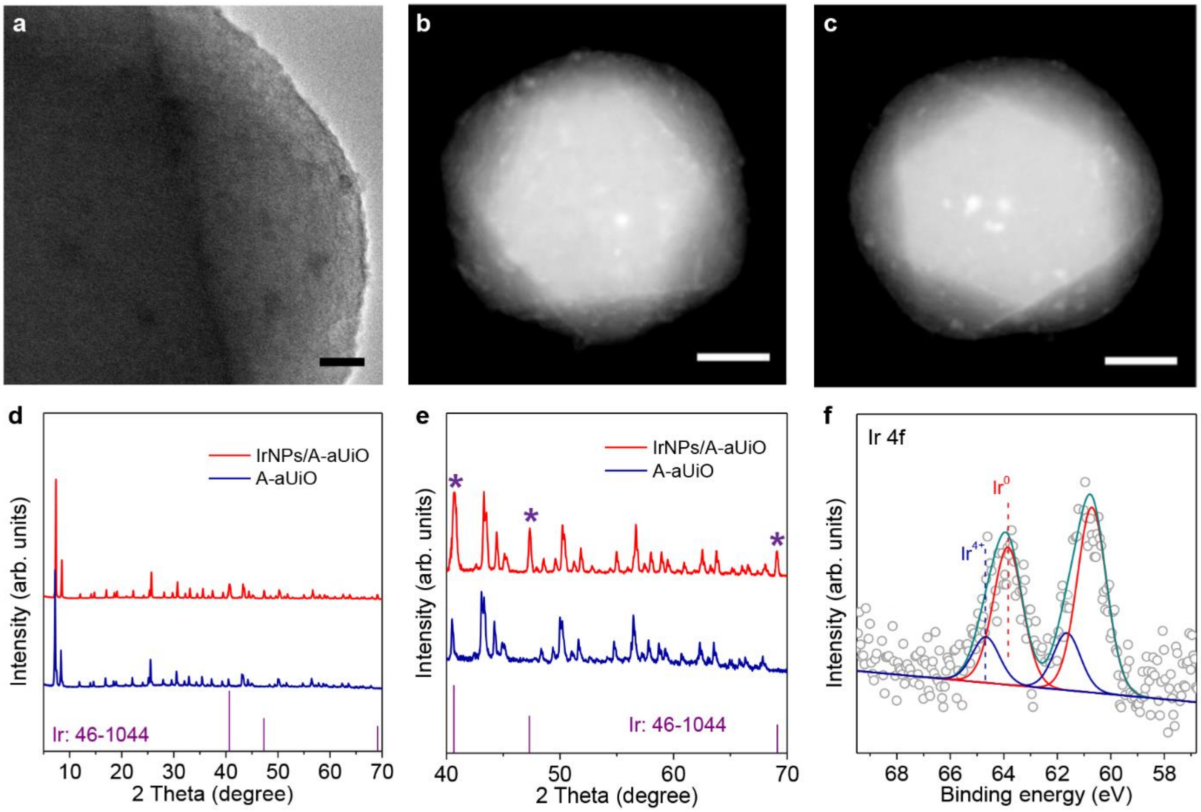

Supplementary Figure 22. Characterization of the as-prepared IrNPs/A-aUiO (4.0 wt.%) samples. (**a**) TEM and (**b**, **c**) HAADF-STEM images of IrNPs/A-aUiO. Ir nanoparticles could be found within the A-aUiO particles through both TEM and STEM images. Scale bar: (**a**) 20 nm and (**b**, **c**) 100 nm. **(d**, **e)** XRD patterns of bare A-aUiO (blue) and IrNPs/A-aUiO (red). XRD peaks for Ir crystals are highlighted by asterisks. (**f**) Ir 4*f* core level XPS spectrum for IrNPs/A-aUiO showing more Ir species in IrNPs/A-aUiO were in metallic state.

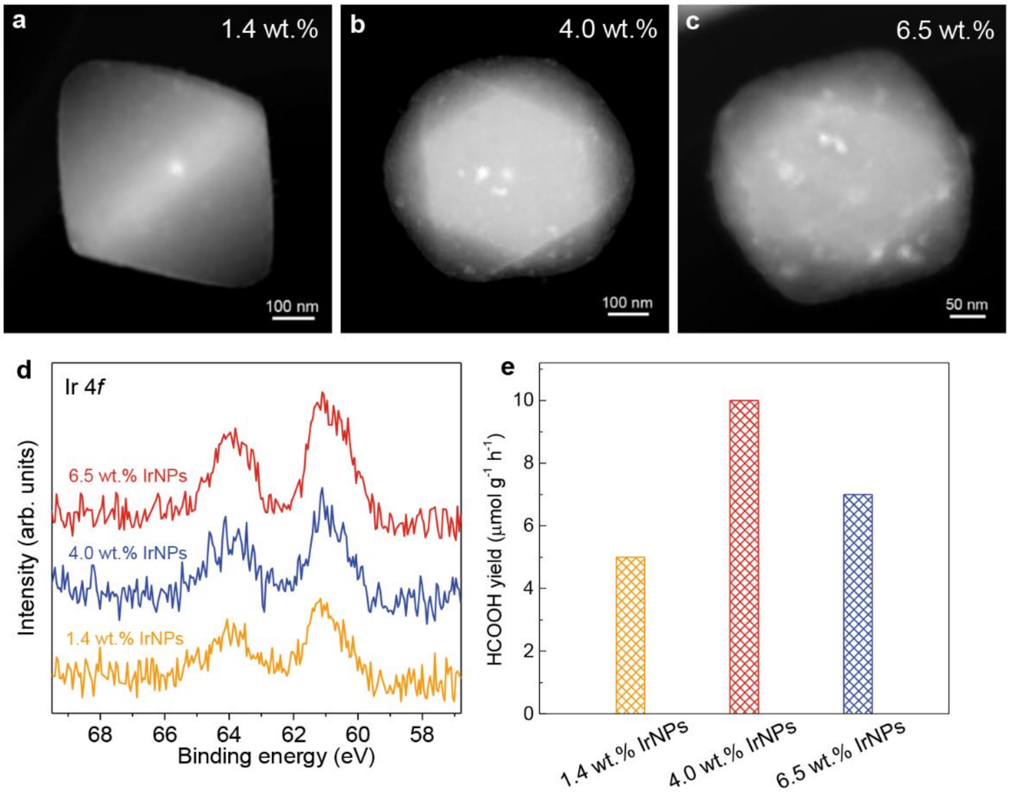

Supplementary Figure 23. Characterizations and photocatalytic CO_2RR performances on IrNPs/A-aUiO with different Ir loadings. (**a–c**) HAADF-STEM images of IrNPs/A-aUiO with the Ir mass loading of (**a**) 1.4 %, (**b**) 4.0 %, and (**c**) 6.5 %. (**d**) Ir 4*f* core level XPS spectra for IrNPs/A-aUiO with different metal loading, showing that the Ir NPs were all mainly in metallic state. (**e**) HCOOH yields on IrNPs/A-aUiO powders (PiG mode) with different Ir loadings under similar reaction conditions.

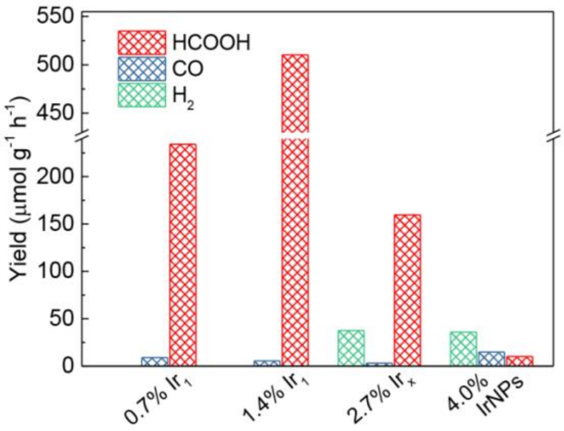

Supplementary Figure 24. Photocatalytic reduction yields for different products on Ir_1/A-aUiO, Ir_x/A-aUiO and IrNPs/A-aUiO catalysts.

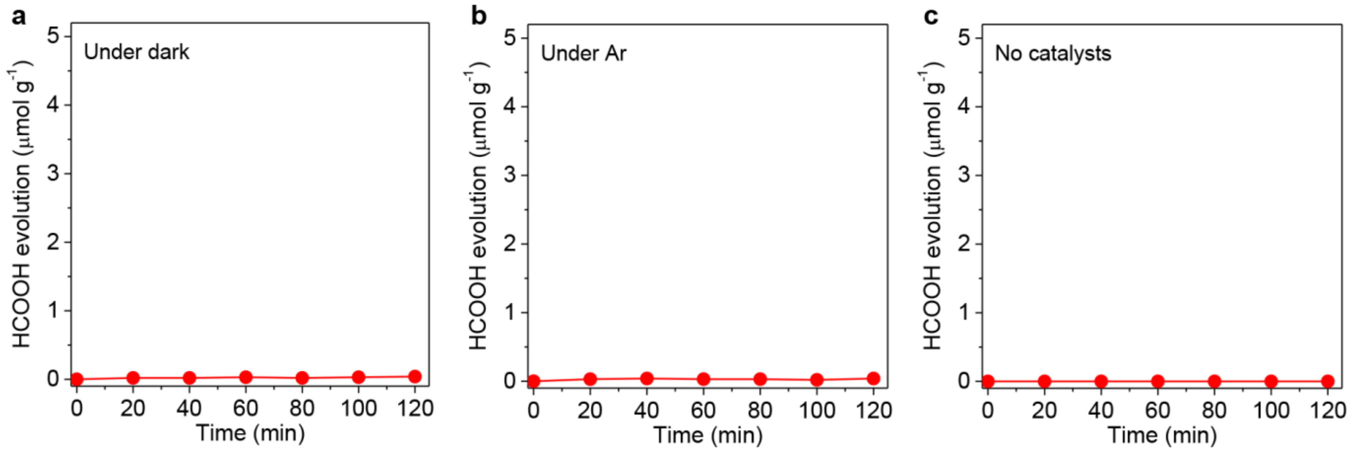

Supplementary Figure 25. Blank experiments for photocatalytic CO_2RR on Ir_1/A-aUiO (1.4 wt.%) powders. No HCOOH evolution could be detected in experiments without (**a**) light irradiation, (**b**) CO_2 feed or (**c**) catalysts (i.e. Ir_1/A-aUiO).

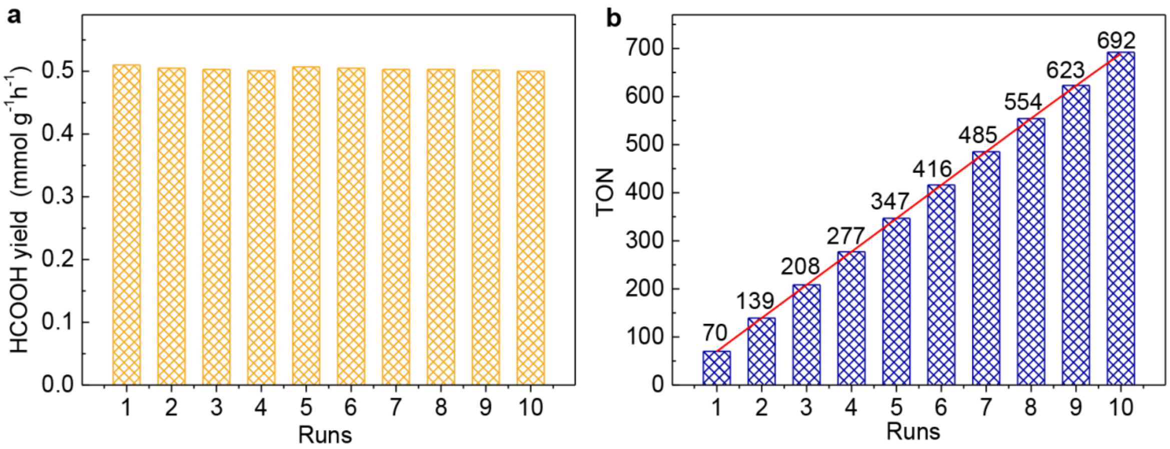

Supplementary Figure 26. Cycling tests for CO_2RR under visible light irradiation. (**a**) HCOOH evolution rates versus cycle numbers. (**b**) TON versus cycle numbers.

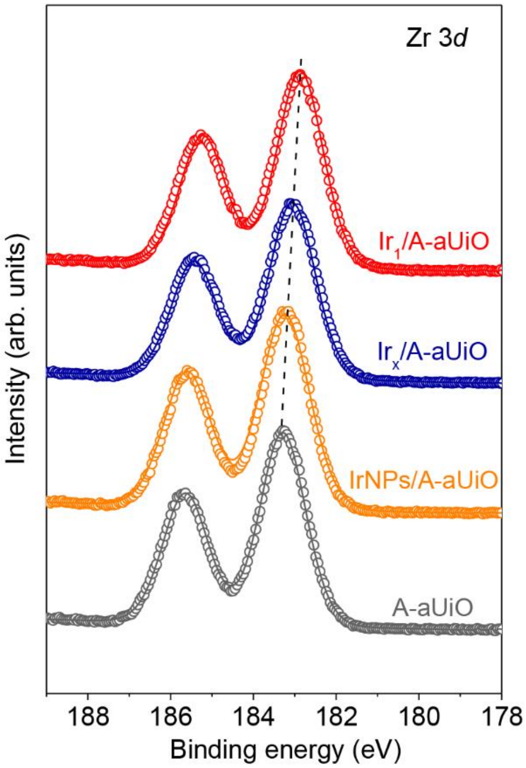

Supplementary Figure 27. XPS characterization revealing the interactions between the Ir species and the A-aUiO supports. The larger shift in Zr 3*d* peaks for Ir_1/A-aUiO suggests the stronger metal-support interactions between the Ir SAs and the Zr_6–O clusters in Ir_1/A-aUiO than those in Ir_x/A-aUiO and IrNPs/A-aUiO.

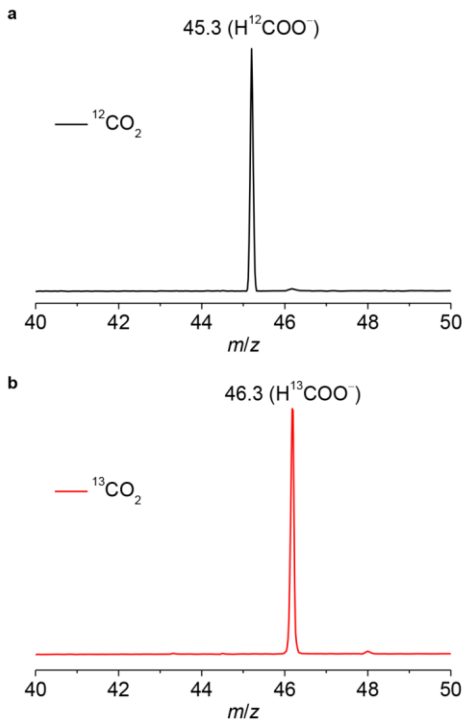

Supplementary Figure 28. ESI-MS profiles for the liquid products of photocatalytic CO_2RR on Ir_1/A-aUiO (1.4 wt.%) powders in the PiS mode with $^{12}CO_2$ and $^{13}CO_2$ as gas reactants. During the photocatalytic reaction, 15 mg of Ir_1/A-aUiO powders were dispersed in a CO_2-saturated H_2O/TEOA (4:1) solution. The dispersion was irradiated by visible light for 4 hours before ESI-MS tests. The peaks at m/z = 45.3 and 46.3 are assigned to $H^{12}COO^-$ and $H^{13}COO^-$, respectively, suggesting that the formates were generated from photocatalytic CO_2 reduction exclusively.

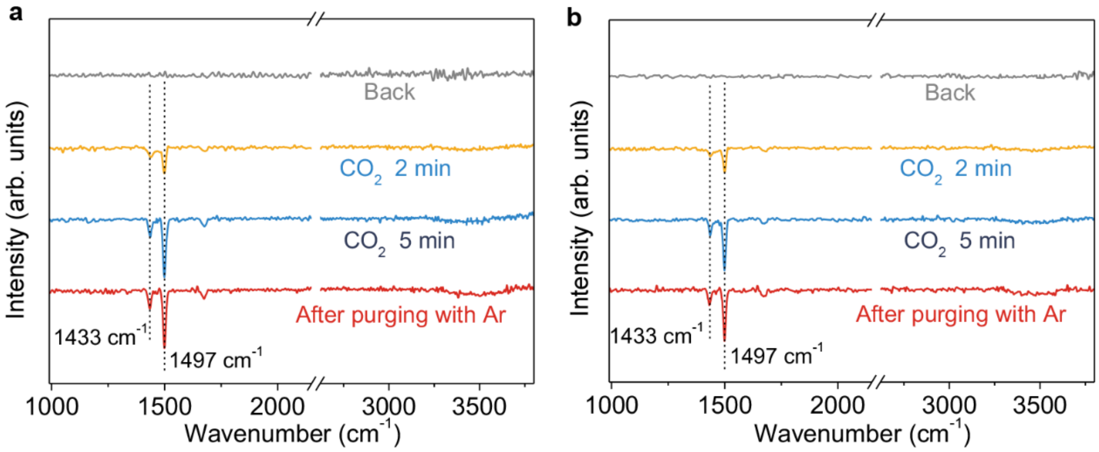

Supplementary Figure 29. *In situ* FT-IR spectra of Ir_1/A-aUiO and A-aUiO catalysts after the exposure to humid CO_2 with different purge time. (**a**) Ir_1/A-aUiO, (**b**) A-aUiO.

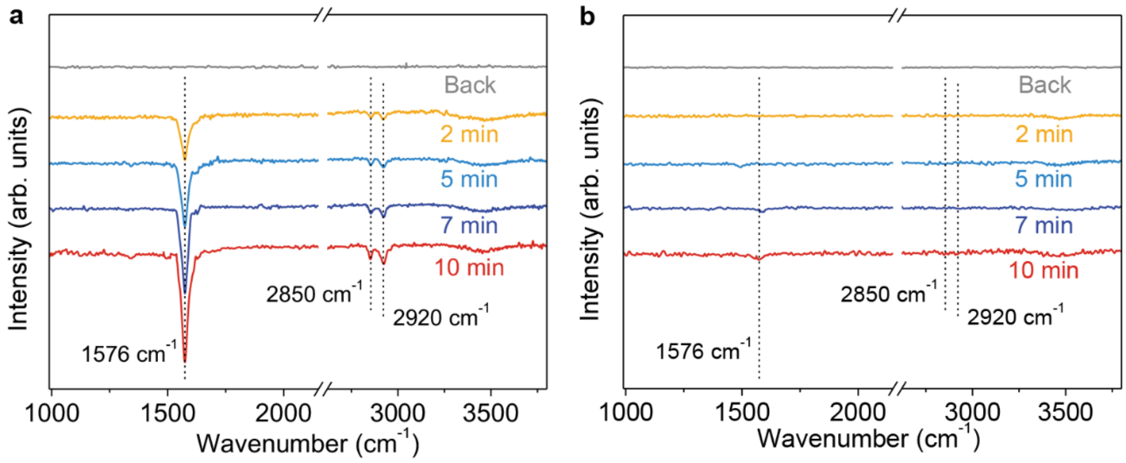

Supplementary Figure 30. *In situ* FT-IR spectra of Ir_1/A-aUiO and A-aUiO catalysts after irradiated by visible light with different time. (**a**) Ir_1/A-aUiO, (**b**) A-aUiO. Before tests, the catalysts were purged with humid CO_2 for 10 min.

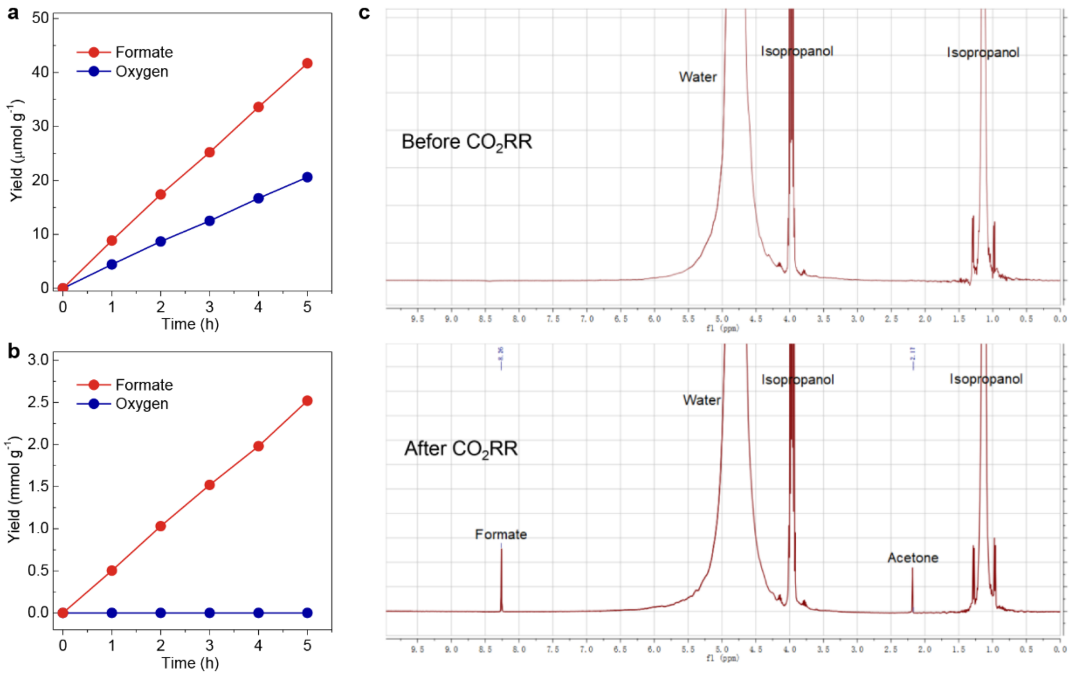

Supplementary Figure 31. (**a, b**) Time course of HCOOH and O_2 evolution on Ir_1/A-UiO powder catalysts without (a) and with (b) using isopropanol as sacrificial agent. (**c**) The ^{1}H NMR spectra for the liquid products in the photocatalytic CO_2RR by using isopropanol as sacrificial agent. The peaks at 8.26 and 2.17 ppm can be assigned to $HCOO^-$ (reduction product of CO_2) and acetone (oxidation product of IPA), respectively.

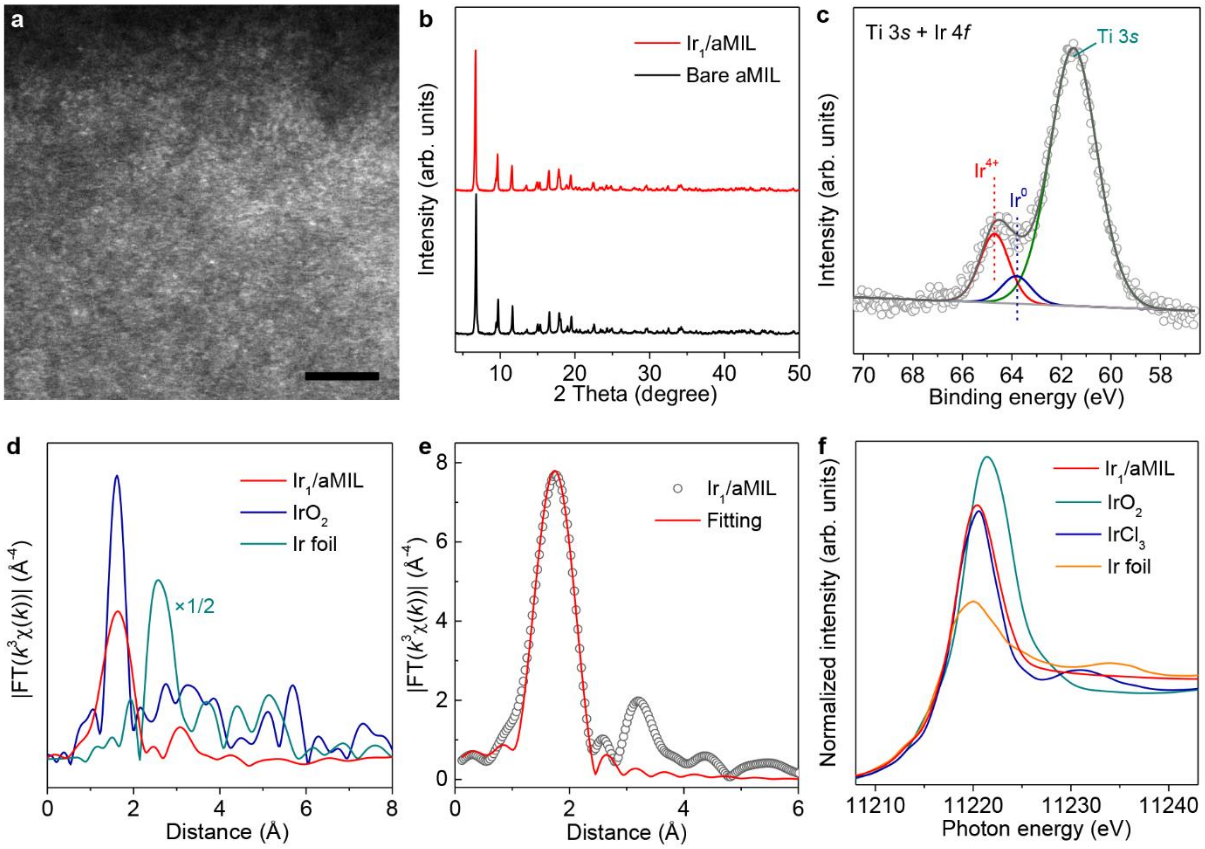

Supplementary Figure 32. Characterization of the as-prepared Ir_1/aMIL (1.3 wt.%) samples. (**a**) High-resolution aberration-corrected HAADF-STEM image of Ir_1/aMIL. Scale bar: 2 nm. (**b**) XRD patterns for bare aMIL (black) and Ir_1/aMIL (red). (**c**) Ir 4*f* core level XPS spectrum for Ir_1/aMIL, showing that most Ir species were in oxidized states. (**d**) Ir L-edge EXAFS spectra of Ir_1/aMIL (red), IrO_2 (blue) and bulk Ir foil (cyan). (**e**) Ir L-edge EXAFS fitting results for Ir_1/aMIL. The presence of Ir–O coordination but no Ir–Ir signals confirmed the atomic dispersion of Ir species in Ir_1/aMIL. (**f**) Ir L-edge XANES spectra for Ir_1/aMIL (red) with bulk Ir foil (orange), $IrCl_3$ (blue) and IrO_2 (cyan) as references. The Ir SAs in Ir_1/aMiL were positively (~ +3) charged.

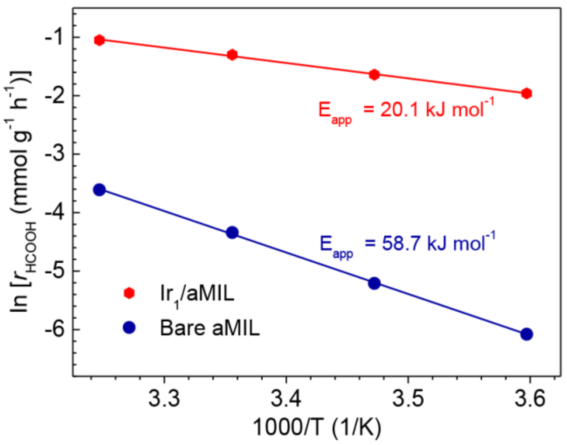

Supplementary Figure 33. Apparent activation energy (E_{app}) for CO_2RR on Ir_1/aMIL and bare aMIL photocatalysts in the PiS mode.

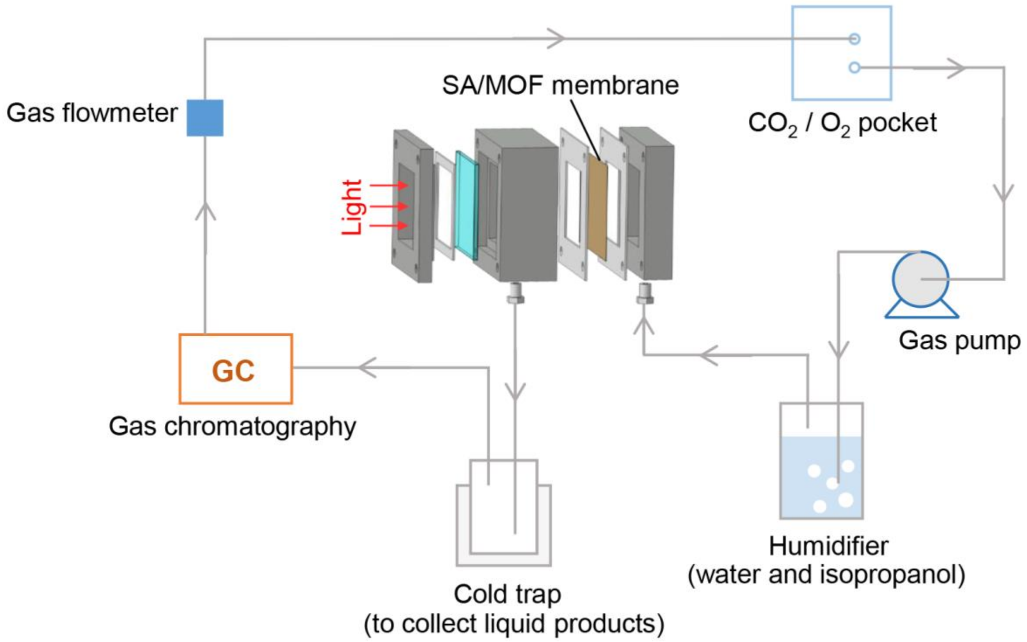

Supplementary Figure 34. Schematic illustration for the setup of photocatalytic CO_2RR and ORR in the gas-membrane-gas (GMG) mode. A gas-tight circulation system with on-line gas chromatography was used to quantify the gas-products of the reaction. The effective geometric area of the SA/MOF membrane is 12.5 cm^2.

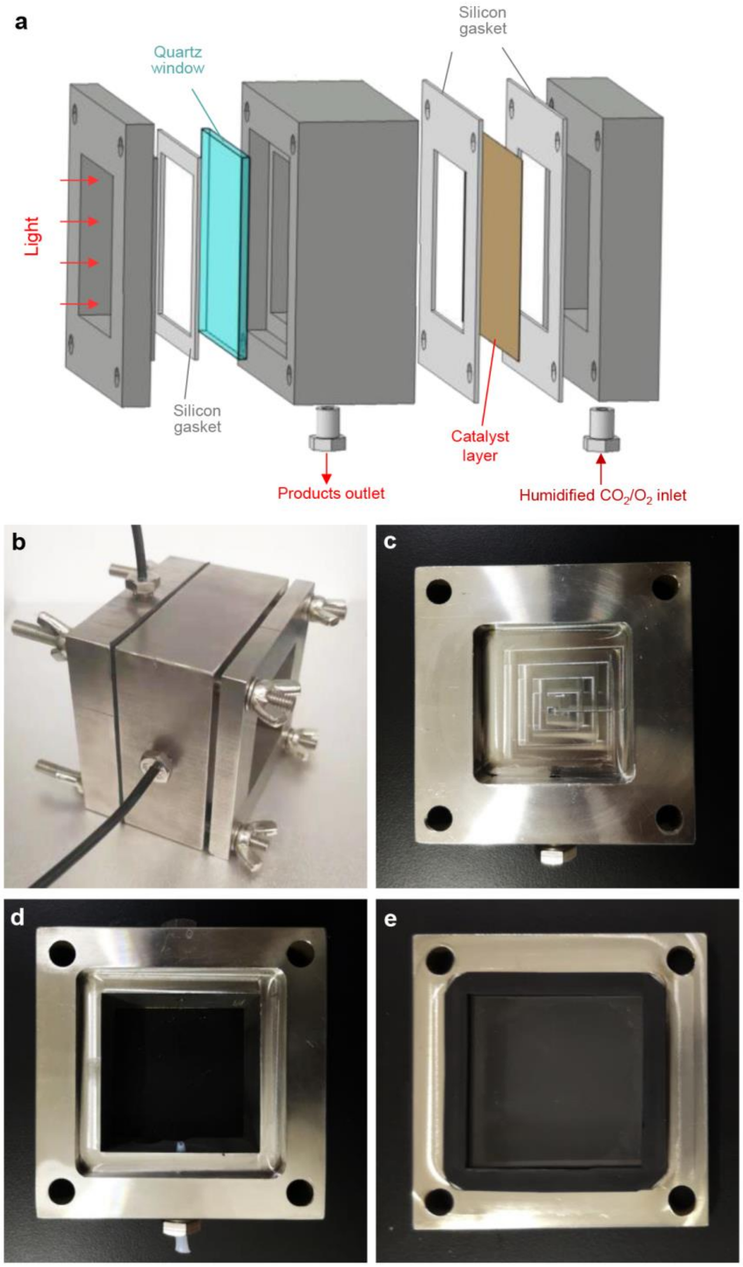

Supplementary Figure 35. Explosive diagram (a) and digital photographs (b–e) of the gas-flow cell used in the experiments.

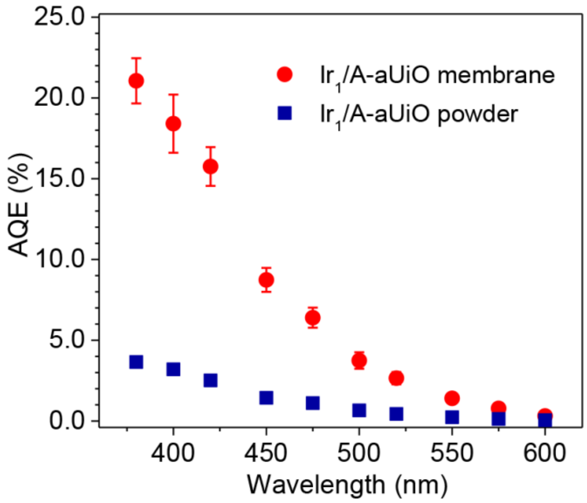

Supplementary Figure 36. Wavelength dependence of apparent quantum efficiency (AQE) during photocatalytic CO_2 reduction on Ir_1/A-aUiO powders and membranes. Error bars represent the standard deviation for three independent catalysis tests.

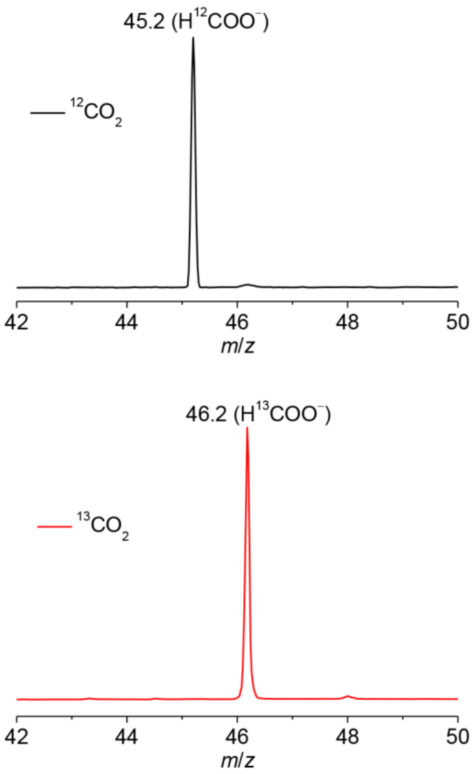

Supplementary Figure 37. ESI-MS profiles for the liquid products of photocatalytic CO_2RR on Ir_1/A-aUiO membranes in the GMG mode with $^{12}CO_2$ and $^{13}CO_2$ as gas reactants. During the test, high-pure CO_2 (99.9999%) was humidified by ultrapure water and cycled continuously in the whole system by a gas-recycle-pump so that the CO_2 and water molecules could continuously pass across the membrane. The membrane was irradiated by visible light for 4 hours before ESI-MS tests. The peaks at m/z = 45.2 and 46.2 are assigned to $H^{12}COO^-$ and $H^{13}COO^-$, respectively, suggesting that the formates were generated from photocatalytic CO_2 reduction exclusively.

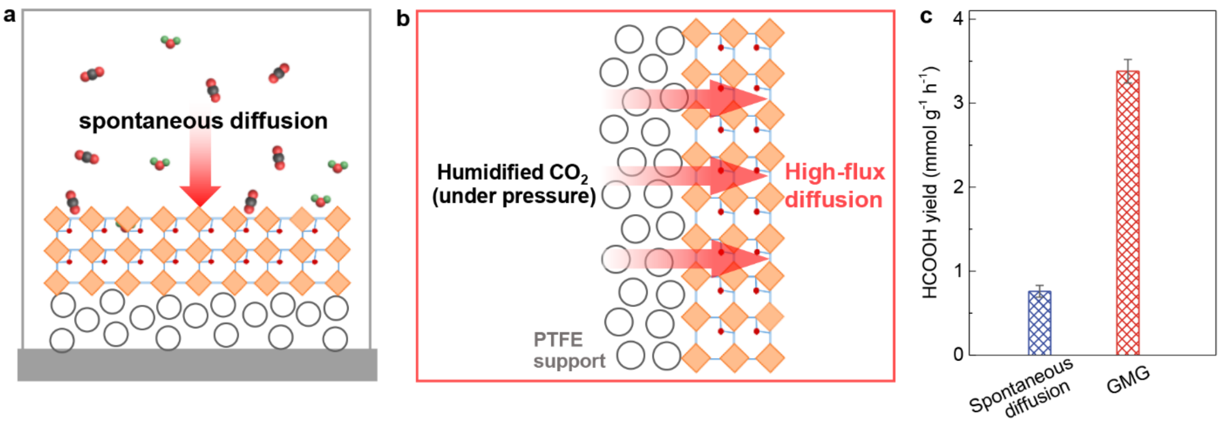

Supplementary Figure 38. Photocatalytic CO_2RR in the spontaneous diffusion mode and the GMG mode. (**a**) In the spontaneous diffusion mode, the diffusion of humidified CO_2 was driven by only spontaneous thermal motion of gaseous molecules (**b**) In the GMG mode, humidified CO_2 gas was fed through the SA/MOF membrane with high-throughput. (**c**) HCOOH yields on 1.4 wt.% Ir_1/A-aUiO membranes in the spontaneous diffusion mode and the GMG mode. Error bars represent the standard deviation for three independent catalysis tests.

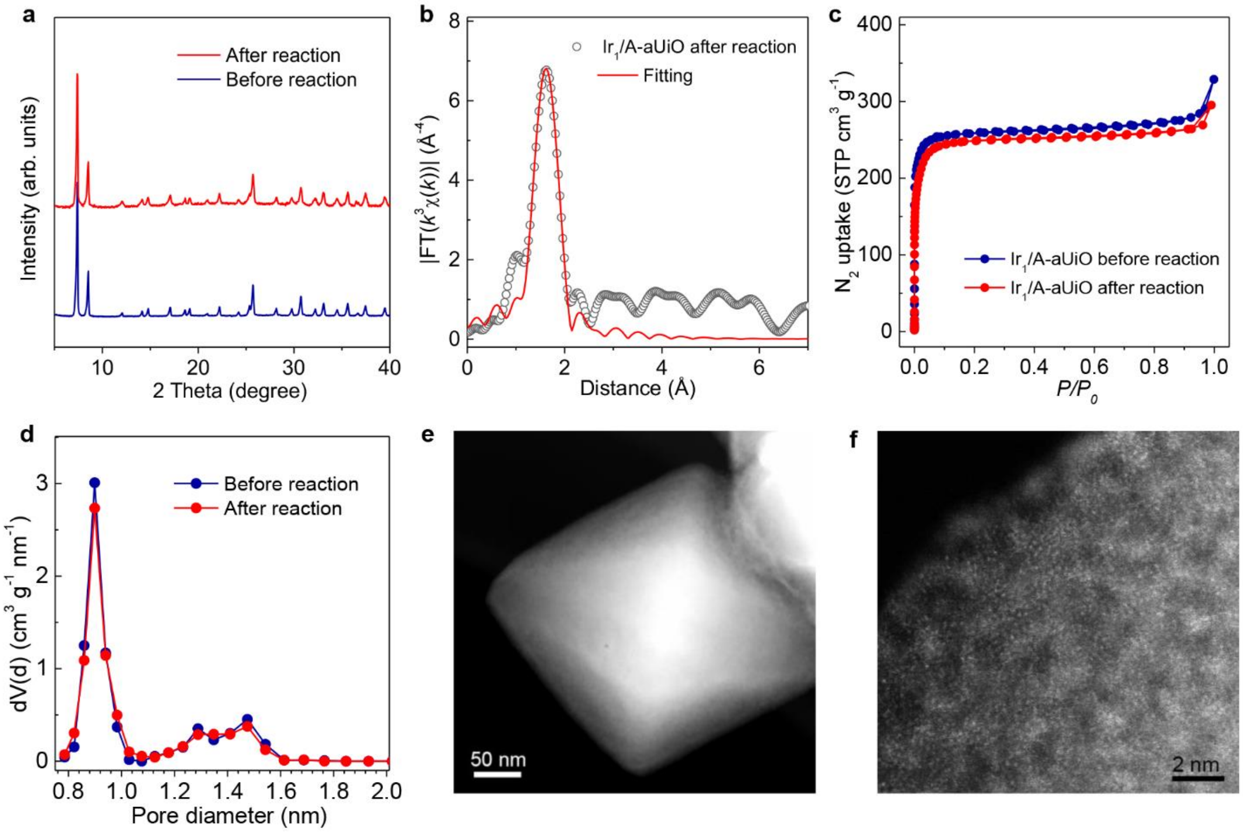

Supplementary Figure 39. (**a**) XRD patterns of 1.4 wt.% Ir_1/A-aUiO catalyst before and after long-term operation (100 h). (**b**) Ir L_3-edge EXAFS fitting results for 1.4 wt.% Ir_1/A-aUiO catalyst after long-term operation. The presence of Ir–O coordination but no Ir–Ir signals confirmed the atomic dispersion of Ir species in 1.4 wt.% Ir_1/A-aUiO. (**c**, **d**) N_2 adsorption-desorption isotherms (c) and pore size distributions (d) of Ir_1/A-aUiO catalyst after long-term operation. (**e**, **f**) STEM (**e**) and high-resolution AC-STEM (**f**) images of Ir_1/A-aUiO catalyst after long-term operation.

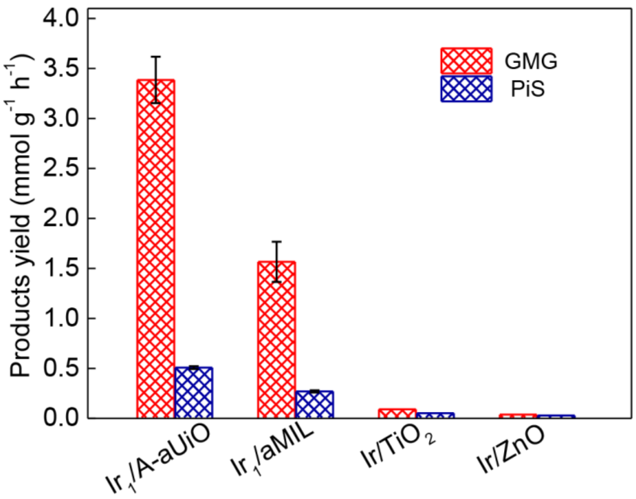

Supplementary Figure 40. Products evolution rates for photocatalytic CO_2RR on Ir_1/A-aUiO, Ir_1/aMIL, Ir/TiO_2 and Ir/ZnO in PiS (blue) and GMG (red) modes. Notably, the main reduction products were HCOOH on Ir_1/A-aUiO and Ir_1/aMIL catalysts, and CO on Ir/TiO_2 and Ir/ZnO, respectively. Error bars represent the standard deviation for three independent catalysis tests.

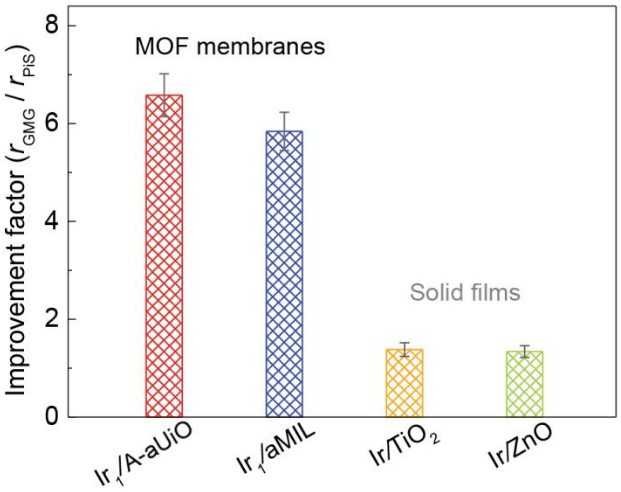

Supplementary Figure 41. Photocatalytic activity improvement factors of different types of membranes calculated from the products (HCOOH for Ir_1/A-aUiO and Ir_1/aMIL, CO for Ir/TiO_2 and Ir/ZnO) yields in PiS and GMG modes. Error bars represent the standard deviation for three independent catalysis tests.

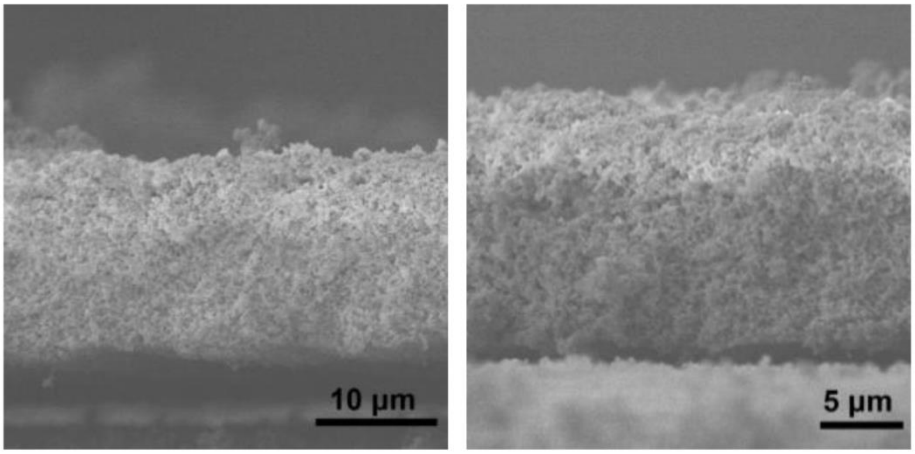

Supplementary Figure 42. Cross-sectional SEM images of the as-prepared PTFE-supported Ir_1/aMIL membrane.

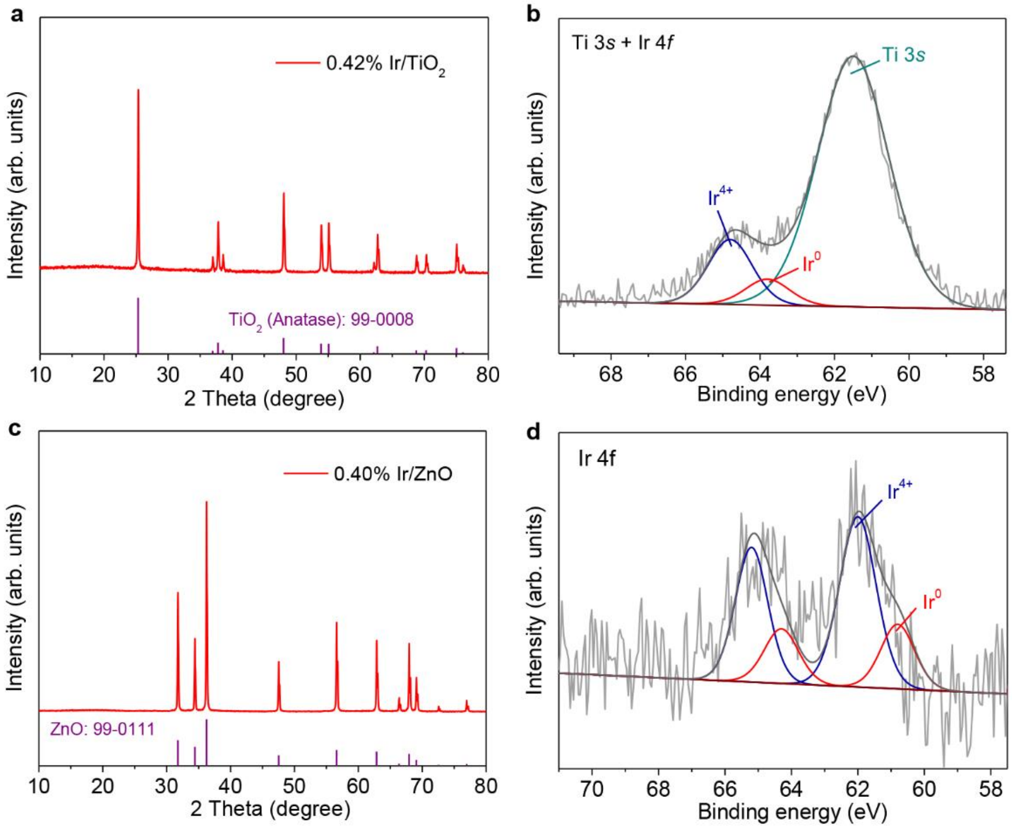

Supplementary Figure 43. XRD and XPS characterization of the as-prepared Ir/TiO_2 (0.42 wt.%) and Ir/ZnO (0.40 wt. %) samples. (**a**) XRD patterns and (**b**) Ir 4*f* core level XPS spectrum for Ir/TiO_2. (**c**) XRD patterns and (**d**) Ir 4*f* core level XPS spectrum for Ir/ZnO. Most Ir species in Ir/TiO_2 and Ir/ZnO samples were in the oxidation states.

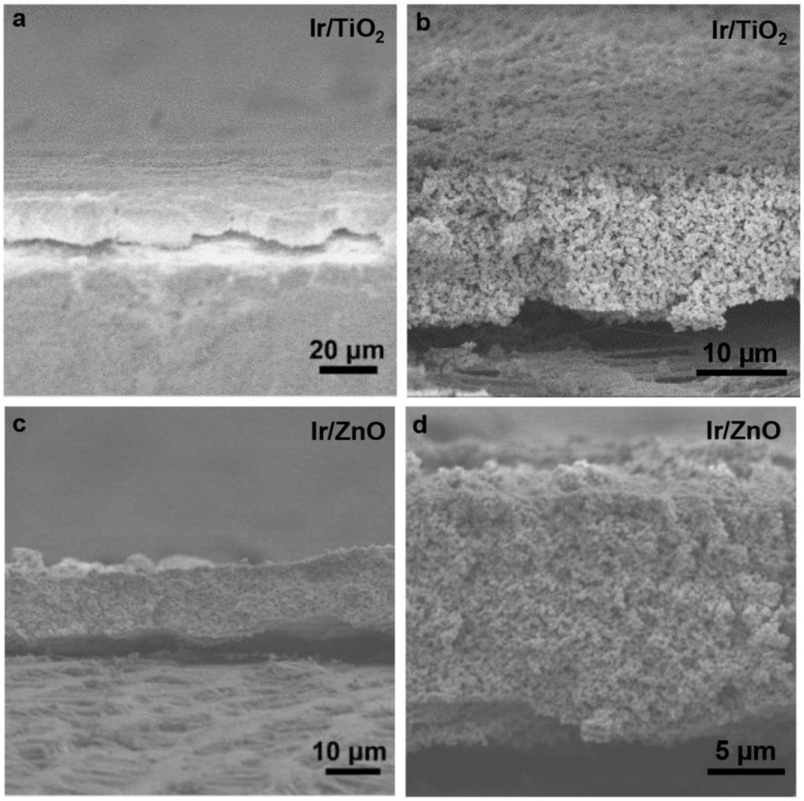

Supplementary Figure 44. Cross-sectional SEM images of the as-prepared PTFE-supported solid films. (**a**, **b**) Ir/TiO_2, (**c**, **d**) Ir/ZnO.

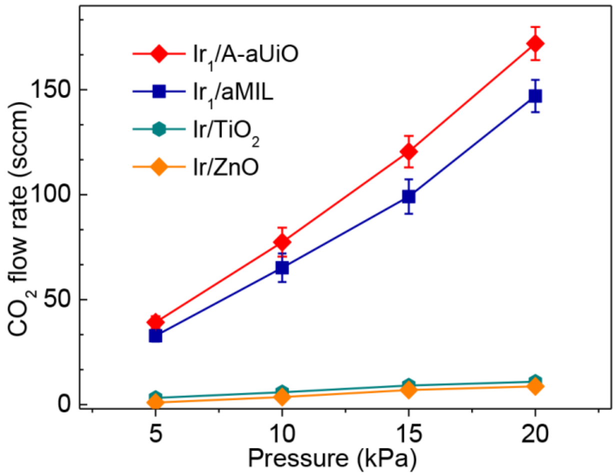

Supplementary Figure 45. Pressure-dependent CO_2 flow rates on SA/MOF (Ir_1/A-aUiO and Ir_1/aMIL) and solid (Ir/TiO_2 and Ir/ZnO) membranes. The gas fluxes through the SA/MOF membranes were much higher than that through the solid membranes at similar pressures, suggesting the ultrahigh porosity of the MOF building blocks.

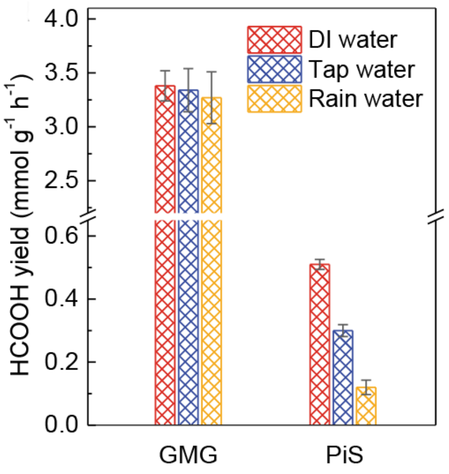

Supplementary Figure 46. HCOOH yields on Ir_1/A-aUiO particles (PiS) and membranes (GMG) by using ultrapure, tap and rain water as proton source, respectively. The photocatalytic reactions were performed under visible light (> 420 nm). Error bars represent the standard deviation for three independent catalysis tests.

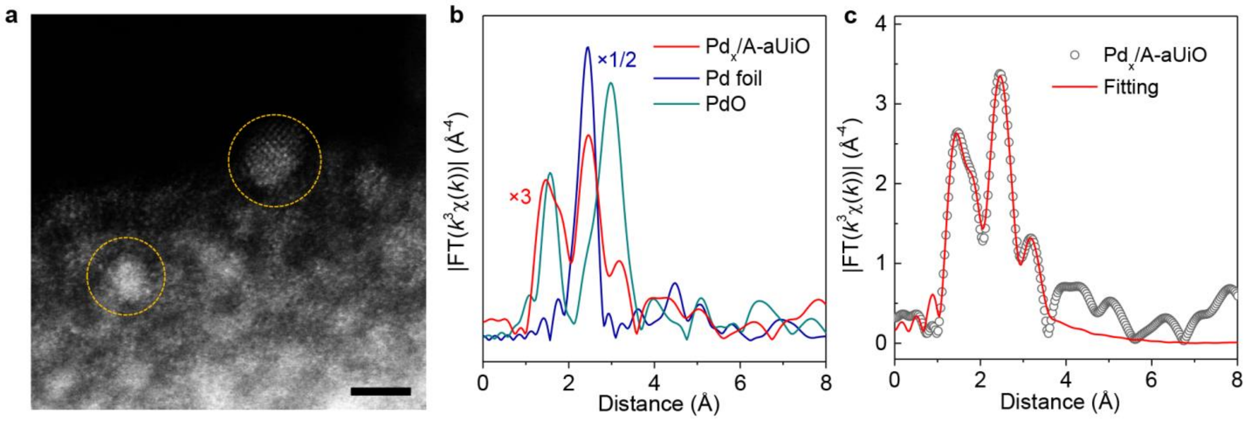

Supplementary Figure 47. AC-HAADF-STEM and EXAFS characterization of the as-prepared Pd_x/A-aUiO samples. (**a**) AC-HAADF-STEM image for Pd_x/A-aUiO. Scale bar: 2 nm. (**b**) Pd K-edge EXAFS spectra of Pd_x/A-aUiO (red), PdO (cyan) and bulk Pd foil (blue). (**c**) Pd K-edge EXAFS fitting results for Pd_x/A-aUiO. The two notable peaks in the region of 1 to 3 Å represent the Pd–O and Pd–Pd contribution, respectively.

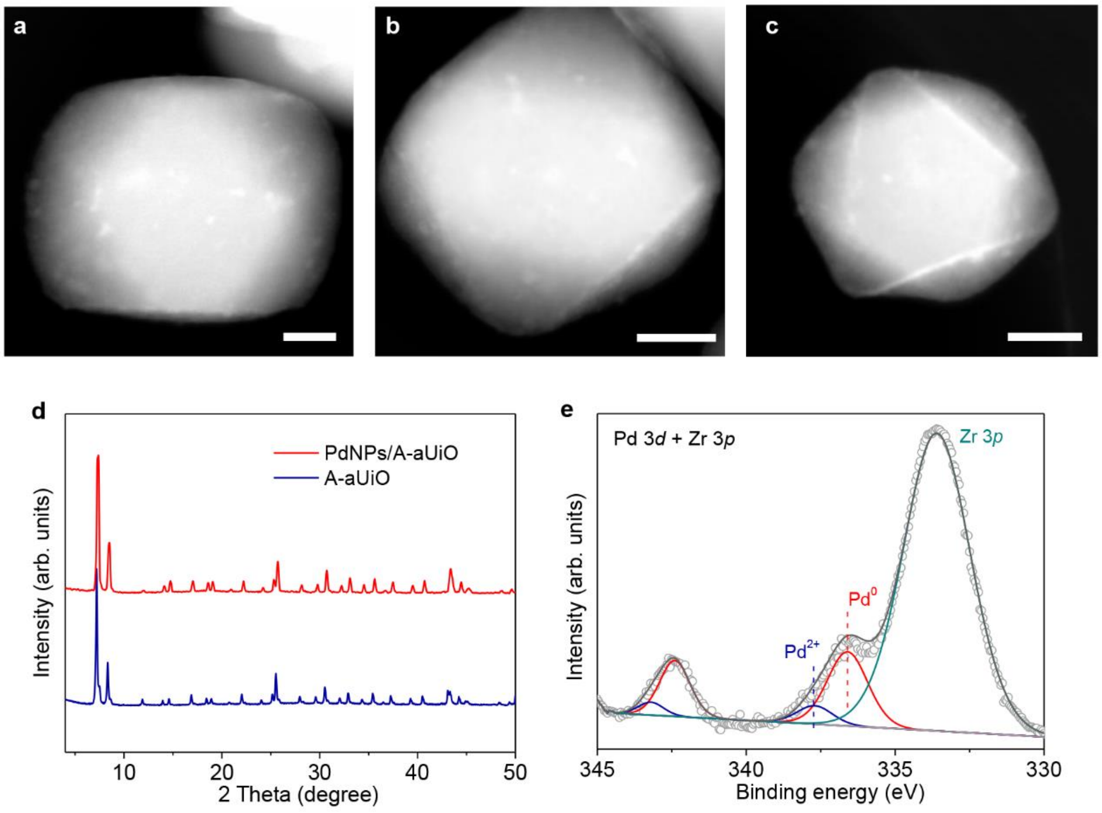

Supplementary Figure 48. Characterization of the as-prepared PdNPs/A-aUiO (1.3 wt.%) samples. (**a–c**) HAADF-STEM images of PdNPs/A-aUiO showing the presence of Pd nanoparticles. Scale bar: 100 nm. (**d**) XRD patterns of PdNPs/A-aUiO (red) and bare A-aUiO (blue). (**e**) Pd 3*d* core level XPS spectrum for PdNPs/A-aUiO, showing that the PdNPs were mostly in the metallic form.

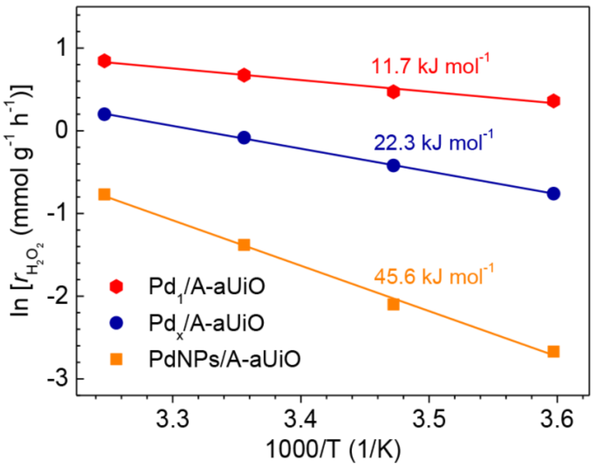

Supplementary Figure 49. Apparent activation energy (E_{app}) for ORR on Pd_1/A-aUiO, Pd_X/A-aUiO and PdNPs/A-aUiO photocatalysts in the PiS mode.

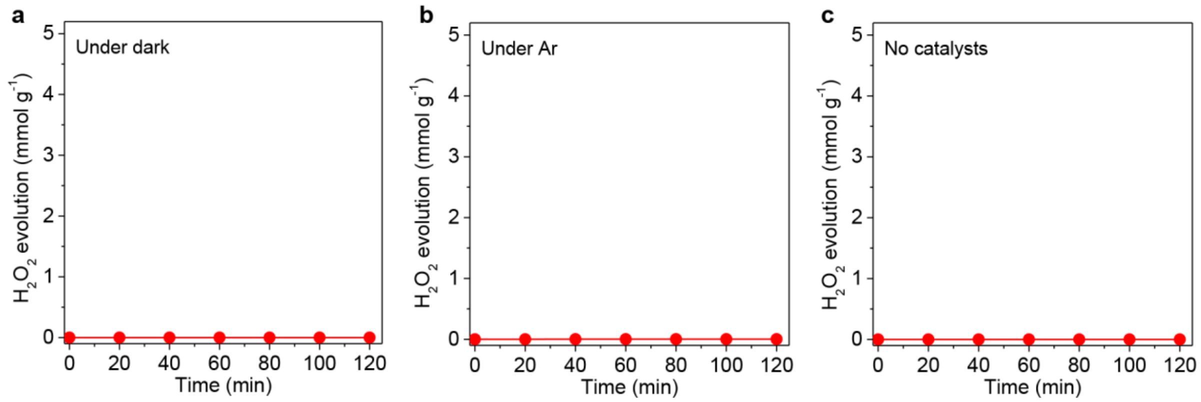

Supplementary Figure 50. Blank experiments for photocatalytic O_2 reduction on Pd_1/A-aUiO powders. No H_2O_2 generation could be detected in experiments without (**a**) light irradiation, (**b**) O_2 feed or (**c**) catalysts (i.e. Pd_1/A-aUiO).

Supplementary Table 1. EXAFS fitting results for as-obtained and recycled catalysts*

Catalysts	Shell	*C.N.*	*R*(Å)	$\sigma^2/\times10^{-3}$(Å^2)	ΔE_0 (eV)
1.4 wt.% Ir_1/A-aUiO	Ir-O	3.8±1.0	2.02±0.01	3.0±1.0	12.2±1.5
	Ir-Ir	–	–	–	–
0.7 wt.% Ir_1/A-aUiO	Ir-O	4.0±1.1	2.04±0.02	3.0±1.0	12.2±1.5
	Ir-Ir	–	–	–	–
Ir_1/A-aUiO (recycled)	Ir-O	4.1±1.0	2.04±0.01	7.7±2.1	8.6±1.8
	Ir-Ir	–	–	–	–
Ir_x/A-aUiO	Ir-O	2.1±0.6	1.99±0.05	4.3±1.4	9.1±1.8
	Ir-Ir	4.3±0.4	2.67±0.02	6.9±1.6	9.1±1.8
Ir_1/aMIL	Ir-O	3.5±0.7	2.02±0.02	3.0±1.0	12.2±1.5
	Ir-Ir	–	–	–	–
Pd_1/A-aUiO	Pd-O	3.7±0.4	2.02±0.02	2.8±1.1	4.6±1.6
	Pd-Pd	–	–	–	–
Pd_x/A-aUiO	Pd-O	2.1±0.3	2.06±0.01	5.0±1.6	0.9±1.5
	Pd-Pd	3.4±0.4	2.72±0.01	4.3±1.6	2.6±1.8

*CN: coordination number

σ^2: Debye-Waller factor (a measure of thermal and static disorder in absorber-scatterer distances)

ΔE_0 is the edge energy shift (the difference between the zero kinetic energy value of the sample and that of the theoretical model).

Supplementary Table 2. Comparison of the photocatalytic CO_2RR performance of the state-of-the-art catalysts with HCOOH as main products.

Catalyst	Light source	Reaction mode	Activity (mmol $g^{-1}h^{-1}$)	Ref.
**Ir_1/A-aUiO**	**Visible light**	**GMG (gas-solid)**	**3.38**	**This work**
**Ir_1/A-aUiO**	**Visible light**	**Liquid-solid**	**0.51**	
AD-MOF-2	Visible light	Liquid-solid	0.443	5
NH_2-MIL-101 (Fe)	Visible light	Liquid-solid	0.445	6
Cobalt complexes[a]	Visible light	Liquid-solid	0.105	7
Eu-Ru(phen)$_3$-MOF	Visible light	Liquid-solid	0.094	8
RuRu'/Ag/mpg-C_3N_4	Visible light	Liquid-solid	0.083	9
N-Ta_2O_5	Visible light	Liquid-solid	0.07	10
PCN-222	Visible light	Liquid-solid	0.06	11
NH_2-UiO-66 (Zr)	Visible light	Liquid-solid	0.026	12

[a] Using carbon nitride (C_3N_4) nanosheets as photosensitizer and support.

Supplementary Table 3. Comparison of photocatalytic H_2O_2 evolution performance of the state-of-the-art catalysts.

Catalyst	Light source	Reaction mode	Activity (mmol $g^{-1}h^{-1}$)	Ref.
**Pd_1/A-aUiO**	**Visible light**	**GMG (gas-solid)**	**10.4**	**This work**
**Pd_1/A-aUiO**	**Visible light**	**Liquid-solid**	**1.74**	
Diimide/C_3N_4/rGO	Visible light	Liquid-solid	1.33	13
g-C_3N_4/PDI	Visible light	Liquid-solid	0.7	14
Au/$BiVO_4$	Visible light	Liquid-solid	0.514	15
g-C_3N_4/BDI	Visible light	Liquid-solid	0.44	16
m-g-C_3N_4	Visible light	Liquid-solid	0.208	17
C_3N_4	Visible light	Liquid-solid	0.125	18
RF535[a]	Visible light	Liquid-solid	0.082	19

[a] Using pure water as proton source without any sacrificial agents.

References:

1. Abdel-Mageed, A. M. & Yaghi, O. M. Highly active and stable single-atom Cu catalysts supported by a metal-organic framework. *J. Am. Chem. Soc*. **141**, 5201–5210 (2019).
2. Long, J. L. & Huang, L. Amine-functionalized zirconium metal–organic framework as efficientvisible-light photocatalyst for aerobic organic transformations. *Chem. Commun.* **48**, 11656 (2012).
3. Li, Y., Wang, Z., Jiang, J. & Xiong, Y. J., Implementing metal-to-ligand charge transfer in organic semiconductor for improved visible-near-infrared photocatalysis. *Adv. Mater.*, **28**, 6959 (2016).
4. Fu, O., Chen, X., Liu, L. Q. & Ye, J. H., Remarkable visible-light photocatalytic activity enhancement over Au/p-type TiO_2 promoted by efficient interfacial charge transfer. *ACS Appl. Mater. Inter*, **11**, 24154 (2019).
5. Li, N. & Lan, Y. Q. Adenine components in biomimetic metal-organic frameworks for efficient CO_2 photoconversion. *Angew. Chem. Int. Ed.* **58**. 5226–5231 (2019).
6. Wang, D. K., Li, Z. H. Fe-based MOFs for photocatalytic CO_2 reduction: role of coordination unsaturated sites and dual excitation pathways. *ACS Catal*. **4**, 4254–4260 (2014).
7. Guo, Z. G., Chen, G., Cometto, C., Ma, B., Lau, K. C., Lau, T. C. & Robert, M. Selectivity control of CO versus $HCOO^-$ production in the visible-light-driven catalytic reduction of CO2 with two cooperative metal sites. *Nat. Catal.* **2**, 801–808 (2019).
8. Yan, Z. H. & Kong, X. J. Photo-generated dinuclear {Eu(II)}2 active sites for selective CO_2 reduction in a photosensitizing metal-organic framework. *Nat. Commun.* **9**, 3353(2018).
9. Kuriki, R., Ishitani, O. & Maeda, K. Robust Binding between carbon nitride nanosheets and a binuclear ruthenium(II) complex enabling durable, selective CO_2 reduction under visible light in aqueous solution. *Angew. Chem. Int. Ed.* **56**. 4867–4871 (2017).
10. Sato, S. & Morikawa, T. Visible-light-induced selective CO_2 reduction utilizing a ruthenium complex electrocatalyst linked to a p-type nitrogen-doped Ta_2O_5 semiconductor. *Angew. Chem. Int. Ed.* **49**. 5101–5105 (2010).
11. Xu, H. Q., Hu, J. H. & Jiang, H. L. Visible-light photoreduction of CO_2 in a metal-Organic framework: boosting electron-hole separation via electron trap states. *J. Am. Chem. Soc.* **137**, 13440–13443 (2015).
12. Sun, D. R. & Li, Z. H. Studies on Photocatalytic CO_2 Reduction over NH_2-UiO-66(Zr) and its derivatives: towards a better understanding of photocatalysis on metal-organic frameworks. *Chem. Eur. J.* **19**, 14279–14285 (2013).
13. Kofuji, Y. & Ichikawa, S. Carbon nitride-aromatic diimide-graphene nanohybrids: metal-free photocatalysts for solar-to-hydrogen peroxide energy conversion with 0.2% efficiency. *J. Am. Chem. Soc.* **138**, 10019–10025 (2016).
14. Shiraishi, Y. et al. Sunlight-driven hydrogen peroxide production from water and molecular oxygen by metal-free photocatalysts. *Angew. Chem. Int. Ed.* **53**. 13454–13459 (2014).
15. Hirakawa, H. & Ichikawa, S. Au nanoparticles supported on $BiVO_4$: effective inorganic photocatalysts for H2O2 production from water and O2 under visible light. *ACS Catal.* **6**, 4976–4982 (2016).
16. Kofuji, Y. & Ichikawa, S. Graphitic carbon nitride doped with biphenyl diimide: efficient photocatalyst for hydrogen peroxide production from water and molecular oxygen by sunlight. *ACS Catal.* **6**, 7021–7029 (2016).
17. Shiraishi. Y. & Ichikawa, S. Effects of surface defects on photocatalytic H_2O_2 production by mesoporous graphitic carbon nitride under visible light irradiation. *ACS Catal.* **5**, 3058–3066 (2015).

18. Shiraishi, Y. et al. Highly selective production of hydrogen peroxide on graphitic carbon nitride (g-C_3N_4) photocatalyst activated by visible light. *ACS Catal.* **4**, 774–780 (2014).
19. Shiraishi, Y. et al. Resorcinol-formaldehyde resins as metal-free semiconductor photocatalysts for solar-to-hydrogen peroxide energy conversion. *Nat. Mater.* **18**, 985–993 (2019).
