# Supplementary material for: Metal-organic framework membranes with single-atomic centers for photocatalytic CO2 and O2 reduction
Source: Nat Commun. 2021 May 11;12:2682. doi: 10.1038/s41467-021-22991-7 (PMC8113524; doi:10.1038/s41467-021-22991-7)
Supplement: Supplementary file 2 — Peer Review [file 41467_2021_22991_MOESM2_ESM.pdf]

REVIEWER COMMENTS

Reviewer #1 (Remarks to the Author):

Recently combination of single atom and defect sites on MOFs is very hot topics. It means that we cannot find new interesting idea in this paper. Pd and Ir are often used as single tom catalysts for CO2 reduction. Because simple combination of several hot keywords, this paper is not attractive for the readers of Nature Comm.

Reviewer #2 (Remarks to the Author):

This manuscript reports fabrication of porous MOF membranes decorated with single-atomic reaction centers for photocatalytic CO2 and O2 reduction. Although the utilization of MOF materials or the noble metal single-atomic reaction centers have been widely reported for CO2 reduction recent years, the application of MOF membrane for a direct conversion of humid CO2 gas into HCOOH at the gas-solid interfaces is quite interesting, and the reported selectivity and activity are impressive. However, there are many concerns need to be addressed before further consideration for publication:
Major concerns:
1. A true photosynthesis should only use water as sacrificial agent. But in this manuscript, it seems that isopropanol was used as sacrificial agent. The authors should make clear this point throughout the manuscript.
2. How the "apparent activation energy" of photocatalysts was obtained? It was missing in the MS.
3. It is known that aUiO is not stable at around 290 oC. Here, for synthesis of IrNPs/d-aUiO, heating at 300 oC under H2/Ar atmosphere for 2 hours was applied, and the structure of aUiO seems to be fully kept. This is quite unusual. Please reconfirm it and explain why.
4. For the stability study, characterizations after photocatalytic reaction, such as PXRD, HRTEM, AC-TEM, EXAFS for recycled samples are indispensable.
5. Why the color of aUiO changed from light yellow to brown after heating at vacuum? The linkers inside were oxidized or not? From MLCT theory, under light irradiation, the linkers were excited and electrons transfer from linker to Zr6 node for catalysis. But it’s also considered that the energy level of Zr is too high that the electrons cannot transfer from linker to it. What's the reaction mechanism here?
6. The bandgap information of photocatalysts and Redox potential of substrates should be provided to better understand the photocatalytic mechanism.
7. For photocatalytic CO2RR, IPA was used as sacrificial agent, but why TEOA was used for isotope experiment? Isotope experiment in GMG mode should also be supplemented.
8. Wavelength-dependent activity together with QE should be provided.
Other minor concerns:
9. Authors stressed that Ir SAs well dispersed in d-aUiO when Ir loading content is less than 1.4wt%. But increasing the Ir loading to 2.7 wt.%, Ir clusters appeared. For Ir SAs synthesis, annealing process under Ar is used. But for Ir cluster synthesis, H2/Ar and higher temperature was used. Thus, the conclusion “Lowering the Ir loading to 0.7 wt.% resulted in sparser dispersion of Ir SAs in Ir1/d-aUiO (Figures S6 and S7), while increasing the loading of Ir to 2.7 wt.% led to the formation of d-aUiO containing both Ir SAs and small clusters (Irx/d-aUiO, Figures S8 and S9)” is arbitrary. Experiments under the same annealing condition but different Ir loading amount should be applied to prove the above conclusion.
10. According to this manuscript, aUiO stands for amino functionalized UiO-66 with acetic acid linker on nodes, while d-aUiO stands for defect amino functionalized UiO-66 without acetic acid. As perfect aUiO has no acetic acid on Zr6 nodes, but 12 linkers around. Therefore, both aUiO and d-aUiO are defect MOFs, and the names of aUiO and d-aUiO used here are inappropriate.
11. The description “Ir SAs (1.4 wt.%) could be evenly dispersed in d-aUiO matrix to form Ir1/d-aUiO particles, as revealed by the scanning electron microscopy (SEM) image (Figure 1c), …” in MS is not right, as even Ir NPs is hard to be distinguished by SEM.

12. For Ir1/d-aUiO, Ar atmosphere was used, and for Irx/d-aUiO, H2/Ar atmosphere was used. But both Pd1/d-aUiO and Pdx/d-aUiO used Ar atmosphere?
13. All four peaks should be integrated in Figure S2d and Figure S2e. Only use peak at 8.10 seems not reliable. This peak may ascribe to amino, and seems not reliable. All four peaks should be referred to which H in 2-aminoterephthalic acid. By the way, peak at 1.89 ascribe to acetic acid, and there are three H contributed the area of 0.18. So, the calculation method for the molar ratio of acetate/2-aminoterephthalic acid was wrong in this MS.
14. Fit parameters of EXAFS are missing.
15. No scale bar for Figure S1. Figure S4C was missing. XPD in Figure S33 should be XRD.

Reviewer #3 (Remarks to the Author):

This study looks at the fabrication and testing of supported MOF membranes for CO2 and O2 photoreduction.
A particular aspect of the material is that they contain single metal atoms dispersed throughout the MOF structure to enhance the photocatalytic response.
There are lots of interesting analyses carried out in this work, which is very much commended.
The downside of this is that it can be difficult to extract the relevant analyses and confirm that they indeed support the claims of the study. Ultimately, there is a sense that no much new information is provided by the study presented here, especially with respect to why SA/MOF are possibly better.

I provide below my analysis/feedback.

The claims of the study as written in the abstract are:
- 1) "breathable metal-organic framework (MOF) membranes decorated with metal single-atoms (SAs) can synergistically promote the diffusion, adsorption, and activation of gas molecules (e.g. CO2, O2) to boost the photocatalytic conversion of them into liquid fuels"
- 2) "Furthermore, the breathable SA/MOF membranes can directly convert humid CO2 gas into HCOOH at the high-throughput gas-solid interfaces, presenting a near-unity selectivity and an unprecedented activity of 3.38 mmol gcat". This claim is related to a comparison with particle in solution systems.

I will start with the second claim.
- The fact that gas-solid set-up is improved compared to gas-liquid set-up is expected due to the limited dissolution of CO2 in water. This explains the increasing number of gas-solid CO2 photoreduction studies being reported in the literature. Hence, it is not clear what new knowledge is generated here. For instance, how does Figure 3a-d bring new insights for the community to use going forward? We note that the use of gas-solid systems (incl. membrane-based gas solid systems) for CO2 photoreduction is not cited in the introduction.
- The "unprecedented activity" and the comparison table (Table S1) do not mean much and cannot be used for comparison since this rate depends on the test conditions and reactor set-up. Following guidelines in the field, the authors should instead report apparent quantum yield or quantum efficiency. I suggest not reporting production rate in the abstract. On that note, one needs to clarify whether the "cat" in "gcat" is the whole material (incl membrane), the MOF or the supported metal.
Regarding the first claim:
- Studies on single atom supported MOF for photocatalysis (particularly solar fuels production) have already been reported. These references (or a selection of them) should be included and the link to prior research in this field must be shown, as well as how this work provides new insight compared to what has been done before.
- A comparison between SA/MOF and NP/MOF composites is made, which is indeed necessary to show the role of SAs. The EXAFs and XPS nicely confirm the contrasting patterns of NPs and SAs. However, it is not clear what the amount of NPs is compared to the amount of SAs (unless I missed this

information). If the amount is different, then how can a comparison be done? What would confirm that the improvement comes from the SA nature of the metal as opposed to an optimised metal content? Overall, the characterisation of all the samples (incl the ones containing NPs) should be conducted at the same level of depth and presented (e.g. elemental analysis, porosity, DRS UV vis, PL, etc…). Otherwise, one is presented with only a partial view of the parameters that can influence the photocatalysis.
- The "synergistic diffusion and adsorption" do not seem to be demonstrated in the study, unless I have missed it. Regarding diffusion, the case is made between PiS and GMG in Figure 3 but the role of the material is not shown. Also, the faster diffusion in the SAF/MOF case compared to the solid film case seems to be speculated not demonstrated. The adsorption aspect is alluded to in Figure S26 and S27, however these has not been any specific adsorption uptake carried out to quantify the amount adsorbed and support the claims related to "higher local concentration of CO2".
- The authors use the term "breathable". What does it actually mean?

In addition to the above comments, here are some points that are important to consider for photocatalytic studies, as well as additional aspects specific to this work:
- The authors claim high porosity of the materials, however the results of their N2 sorption analyses are not presented (unless I missed it).
- The authors indicate a selectivity near 100% but the nature of the other products identified is not presented. One needs to know the products distribution (incl. liquid and gaseous products).
- The optoelectronic properties of the materials are not investigated in much depth. Only some of the UV vis spectra are shown. The band diagrams of the materials are not presented.
- Having an understanding of the photoreaction mechanisms would bring novelty to the work. The optoelectronic analysis mentioned above along with the in-situ FTIR analyses that the authors initiated would be extremely useful in this regard.
- "Ir clusters and nanoparticles, the Ir SAs were more electron-deficient as revealed by X-ray absorption near-edge structure (XANES) and X-ray photoelectron spectroscopy (XPS) analysis (Figures 2e and S24). Such differences in electronic structures may thereby result in their different catalytic reactivity. " Why would that be the case? Wouldn't electron-rich sites be favourable for a reduction reaction? Again, we are missing an understanding of the distinct behaviour of SA/MOF vs non SA/MOF.
- 13CO2 tests and other control tests are missing for GMG (and also for the O2 reduction study).
- Post-photoreduction characterisation of the materials are missing.
- The products of the oxidation reaction are not indicated.
- Figure 1 and Figures S4, S6, S8: why are some dots highlighted and not others? What makes one identify them as Ir/Pd?

Response to Referees

Reviewer #1:

Recently combination of single atom and defect sites on MOFs is very hot topics. It means that we cannot find new interesting idea in this paper. Pd and Ir are often used as single tom catalysts for CO_2 reduction. Because simple combination of several hot keywords, this paper is not attractive for the readers of *Nature Commun*.

Author Reply:

Thanks for the Reviewer's comments. We feel that we should point out the breakthroughs in our manuscript to address the Reviewer's concerns, and to distinguish our work from the simple combination of single atom and MOFs.

First, the aim of this manuscript is not just simply combining single atoms and MOFs. What we would like to share with readers is that we can build a programmable photocatalytic platform to convert the permanent gas (e.g., CO_2, O_2, and even N_2) into value-added chemicals with high efficiency. With rational control of the chemical structures of MOFs, we can promote the photocatalytic conversion at the active centers and the mass transfer of gaseous reactants simultaneously. On one hand, we can rationally modify the photoelectric properties of the MOF matrix and the catalytic properties of the metal centers to optimize the chemical conversion of permanent gas (e.g. CO_2 and O_2). On the other hand, we can build Gas-Membrane-Gas (GMG) reaction interfaces consisting of numerous interconnected nanoreactors, which can significantly promote the mass transfer of such three-phase reactions that always encounter severe diffusion limitations in the conventional gas-solution-solid interfaces.

Second, to our knowledge, Ir single atom (IrSA) catalysts have been reported recently for thermo-catalytic CO_2 hydrogenation (e.g., *JACS*, 2017, 139, 17747; *Chem*, 2019, 3, 693) to generate formate ($CO_2 + H_2 \rightarrow HCOOH$). However, research on systematic modulation of Ir species in heterogeneous catalysts for efficient photocatalytic conversion of CO_2 to HCOOH remain elusive. In this work, we carefully modulate the chemical and electronic structures of Ir species and prove that IrSAs exhibit much higher selectivity and activity towards formate production than Ir clusters and nanoparticles due to their unique electronic structures. That is, through tuning the strong metal-support interaction between Ir species and MOF matrix, we can significantly promote the chemical conversion of CO_2 to HCOOH.

Third, as we mentioned above, another overwhelming challenge for heterogeneous photocatalytic reduction of such permanent gas is their low solubility and slow diffusion in aqueous solutions, which significantly retard the reaction efficiency in the conversional reaction design that disperses catalyst particles and bubbles gaseous reactants into aqueous solutions. In contrast, thanks to the porous structures of MOFs, we can thereby build a SA/MOF membrane consisting of numerous nanoreactors with vast reaction interfaces. Especially, with humidified gas stream as reactants, we can avoid the use of bulk aqueous solutions and turn the gas-solution-solid reaction interface to a quasi-gas-solid interface that can significantly promote the diffusion of gas-phase reactants. In addition, compared to the conventional method using gas solutions (with very low gas concentrations) as reactants, such reaction interface design can significantly increase the molecular ratio of gas/water near the catalytic centers so that to further promote the adsorption of gaseous reactants over the catalytic centres. As a result, the Ir_1/A-aUiO membrane exhibited an apparent quantum efficiency (AQE) of 15.76% at

420 nm in the GMG mode with the CO_2 flow rate fixed at 120 standard cubic centimeters per minute (sccm), which is more than 6 times that showed by Ir_1/A-aUiO particles (2.51%) in the conventional PiS mode.

In addition, we then show that this photocatalysis promotion strategy does not only work in CO_2 reduction, but also can be applied to the direct reduction of O_2 into H_2O_2, which is another essential but challenging photocatalytic reaction (*Nat. Mater,* 2019, 18, 985). This time, Pd single atoms (Pd SAs) are anchored onto our MOF matrix, and the Pd/MOF membranes also catalyze the O_2-to-H_2O_2 conversion with high efficiency. In the GMG mode, the Pd_1/A-aUiO membrane exhibited an H_2O_2 evolution rate of 10.4 mmol g_{cat}^{-1} h^{-1}, significantly outperforming the best reported values (1.33 mmol g_{cat}^{-1} h^{-1}, *JACS*, 2016, 138, 10019). Moreover, the ongoing work in our lab further prove that such SA/MOF membrane can also promote the photocatalytic fixation of N_2 to NH_3. Therefore, our experiments prove that such SA/MOF membranes can serve as a universal platform for the photocatalytic conversion of major permanent gas (i.e., CO_2, O_2, and N_2) into value-added liquid chemicals under visible light and ambient conditions efficiently.

We hope our justification and our revisions according to the Reviewers' suggestions (*see below*) can address the Reviewer's concerns, and our manuscript can be accepted for publication in *Nature Commun*.

Reviewer #2:

This manuscript reports fabrication of porous MOF membranes decorated with single-atomic reaction centers for photocatalytic CO_2 and O_2 reduction. Although the utilization of MOF materials or the noble metal single-atomic reaction centers have been widely reported for CO_2 reduction recent years, the application of MOF membrane for a direct conversion of humid CO_2 gas into HCOOH at the gas-solid interfaces is quite interesting, and the reported selectivity and activity are impressive. However, there are many concerns need to be addressed before further consideration for publication.

Author Reply:

Thanks for the Reviewer's positive comments and support for our work.

We have made careful revisions according to the Reviewers' suggestions to improve the quality of our manuscript. We hope that the revised manuscript can address the Reviewers' concerns and can be accepted for publication in *Nature Commun*.

R2Q1. A true photosynthesis should only use water as sacrificial agent. But in this manuscript, it seems that isopropanol was used as sacrificial agent. The authors should make clear this point throughout the manuscript.

Author Reply:

Thanks for the Reviewer's kind suggestion.

Isopropanol was used as sacrificial agent in this work. We have revised the manuscript accordingly (**Lines 15–17, Page 5**).

R2Q2. How the "apparent activation energy" of photocatalysts was obtained? It was missing in the MS.

Author Reply:

Thanks for the Reviewer's kind reminder.

The "apparent activation energy" was obtained from a series of photocatalytic reactions at different temperatures (e.g., 5, 15, 25 and 35 °C, **Figure 2f** in the manuscript) and then fitting the Arrhenius plots [ln(r_{HCOOH}) vs 1/T]. We have revised the manuscript accordingly (**Line 8, Page 6**).

R2Q3. It is known that aUiO is not stable at around 290 °C. Here, for synthesis of IrNPs/*d*-aUiO, heating at 300 °C under H_2/Ar atmosphere for 2 hours was applied, and the structure of aUiO seems to be fully kept. This is quite unusual. Please reconfirm it and explain why.

Author Reply:

Thanks for the Reviewer's questions.

According to previous reports (*JACS*, 2013, 135, 10525; *JACS*, 2020, 142, 3174), UiO-66 crystal can stay stable up to nearly 500 °C in air. And we have also double checked our data and carried out additional experiments to verify the stability of aUiO under relatively high temperatures. In short, our findings confirm that aUiO is stable under our reaction

conditions.

First, we carefully checked the XRD patterns and the FT-IR spectra of aUiO before and after thermal treatment at 300 °C under H_2/Ar atmosphere for 2 hours. As shown in **Fig. R1**, no obvious differences could be observed in XRD patterns or FT-IR spectra for aUiO after thermal treatment at 300 °C, indicating that the geometrical and chemical structures of aUiO were maintained after the thermal treatment.

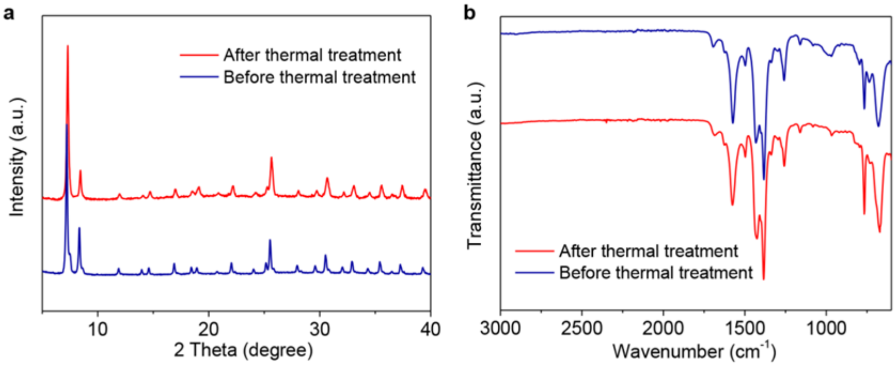

Fig. R1. XRD patterns (**a**) and FT-IR spectra (**b**) for aUiO before (blue) and after (red) thermal treatment at 300 °C under H_2/Ar atmosphere.

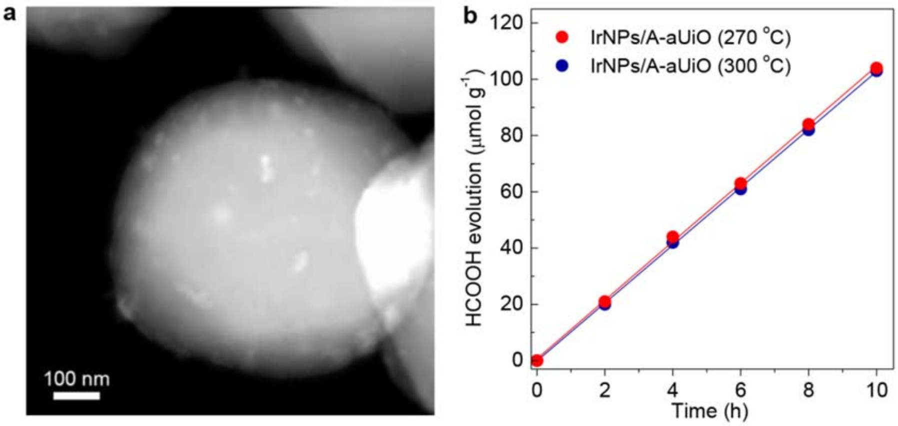

Fig. R2. (**a**) HAADF-STEM image of IrNPs/A-aUiO catalyst prepared at 270 °C. (**b**) HCOOH evolution rates for photocatalytic CO_2RR on IrNPs/A-aUiO prepared at 270 °C (red) and 300 °C (blue).

Second, we also tried to prepare the IrNPs/A-aUiO (As suggested by **Reviewer 2**, we use "A-aUiO (activated a-UiO)" instead of "*d*-aUiO" to avoid any possible ambiguity in our revision, *please see our reply to* ***R2Q10***) catalyst at a lower temperature (270 °C) and compared their photocatalytic CO_2RR performances with those prepared at 300 °C. If the aUiO structures were destroyed by thermal treatment at 300 °C, these two samples should show quite different catalysis behaviors. However, as shown in **Fig. R2**, IrNPs/A-aUiO particles prepared at 270 and 300 °C exhibited similar photocatalytic CO_2RR activities, further suggesting that the aUiO matrix were stable after thermal treatment at 300 °C.

We hope such additional evidence can address the Reviewer's concerns.

R2Q4. For the stability study, characterizations after photocatalytic reaction, such as PXRD, HRTEM, AC-TEM, EXAFS for recycled samples are indispensable.

Author Reply:

Thanks for the Reviewer's kind suggestion.

We have performed PXRD, EXAFS, and AC-HADDF-STEM characterizations for the photocatalysts after long-term (100 h) operation. As shown in **Fig. R3**, the chemical structures of the Ir_1/A-aUiO catalysts are very stable during the long-term reaction.

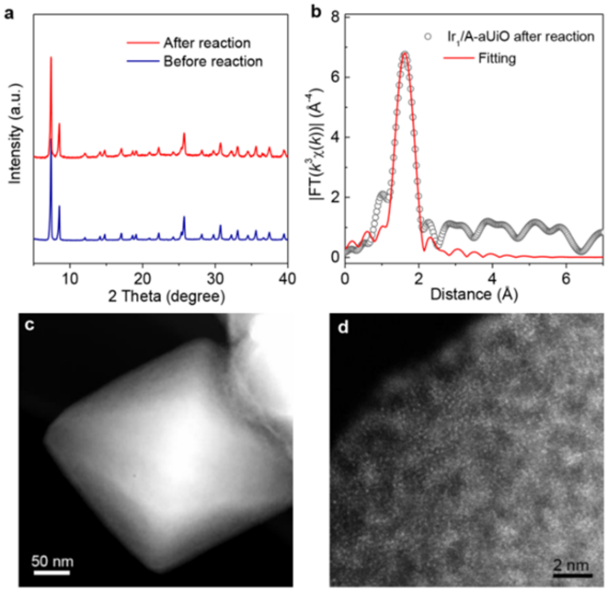

Fig. R3. (**a**) XRD patterns of 1.4 wt.% Ir_1/A-aUiO catalyst before and after long-term operation (100 h). (**b**) Ir L_3-edge EXAFS fitting results for 1.4 wt.% Ir_1/A-aUiO catalyst after long-term operation. The presence of Ir–O coordination but no Ir–Ir signals confirmed the atomic dispersion of Ir species in 1.4 wt.% Ir_1/A-aUiO. (**c**, **d**) STEM (**c**) and high-resolution AC-STEM (**d**) images of Ir_1/A-aUiO catalyst after long-term operation.

We have also revised the manuscript accordingly (**Lines 16–18, Page 8**, and **Supplementary Figure 38**). We hope these revisions can address the Reviewer's concerns.

R2Q5. Why the color of aUiO changed from light yellow to brown after heating at vacuum? The linkers inside were oxidized or not? From MLCT theory, under light irradiation, the linkers were excited and electrons transfer from linker to Zr_6 node for catalysis. But it's also considered that the energy level of Zr is too high that the electrons cannot transfer from linker to it. What's the reaction mechanism here?

Author Reply:

Thanks for the Reviewer's valuable questions.

For pristine UiO-66 (with no $-NH_2$ groups or any defects), the energy gap between the ligand and the Zr–O nodes were too large (> 3.5 eV) to absorb visible light (*J. Mater. Chem. A*, 2018, 6, 8507). Modifying the ligand (i.e., *p*-phthalic acid) with $-NH_2$ groups can effectively reduce the band gap to about 2.8 eV since the electron-donor effect of the $-NH_2$ groups can push up the bands of the ligand (*JACS*, 2020, 142, 19339). Moreover, according to previous theoretical (*Chem Mater*, 2017, 29, 3006) and experimental (*JACS*, 2013, 135, 10525) studies, high-temperature activation would partially remove

the acetates (used in synthesis) that are coordinated to the Zr–O nodes, resulting in the so-called "missing linkers" defects and the coordination defects on the Zr–O nodes. Such defects would shift the energy bands of A-aUiO so that they can adsorb visible light. In addition, the excited states of the organic linkers of aUiO can also assist in the LMCT procedure (*Chem. Eur. J.*, 2013, 19, 14279; *JACS*, 2015, 137, 13440). Upon visible irradiation, the electrons can be first stimulated to the excited states of the ligands and then transferred to the Zr–O clusters, which can further extend the absorption of aUiO to the visible region.

We have also carried out more characterizations to reveal the band structures of the as-obtained photocatalysts before/after thermal activation. As shown in **Fig. R4a**, the band gap for aUiO is about 2.82 and 2.47 eV before and after activation, respectively. The Mott–Schottky measurements conducted at four different frequencies suggest that the conduction band position was about −0.91 V and –0.60 V vs. NHE for aUiO before and after activation, respectively (**Fig. R4b,c**). The valence band (i.e., HOMO) potential for aUiO before and after activation is then calculated to be 1.91 V and 1.87 V vs. NHE, respectively. That is, the abundant coordination defects on the Zr–O clusters could effectively lower the position of the bottom of the conduction band (i.e., LUMO) while the HOMO on the ligands remain almost unchanged after the thermal activation. As a result, the band gap for aUiO could be significantly reduced by about 0.35 eV, and the activated aUiO can thus absorb light in the visible region.

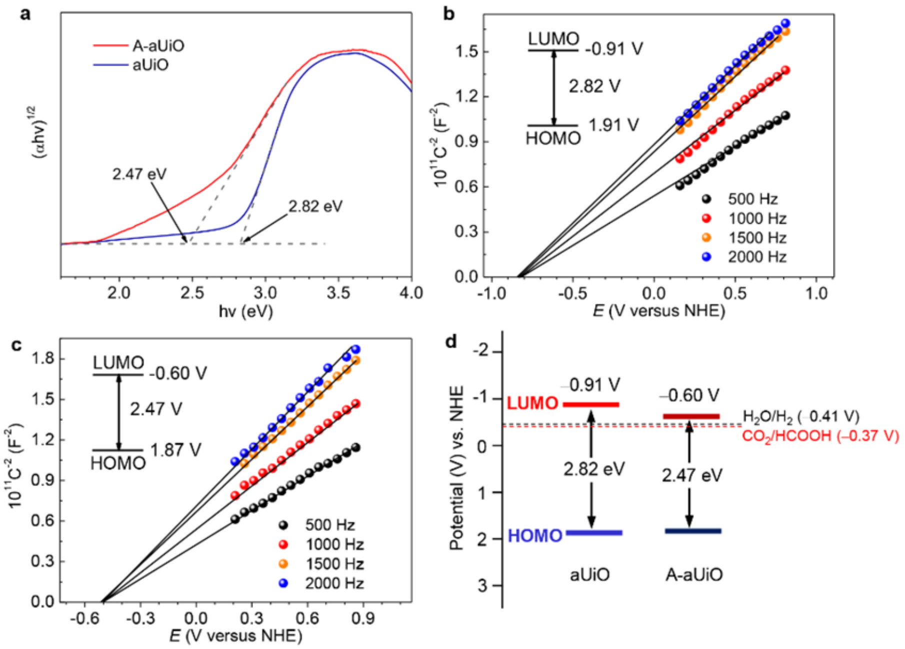

Fig. R4. Electronic band structures of aUiO samples before and after activation. (**a**) Optical band gaps determined by UV-vis diffuse reflectance, where α and ν represent the absorbance and wavenumber, respectively. (**b**, **c**) Mott-Schottky (M-S) curves for (**b**) aUiO and (**c**) A-aUiO. Insets in (b, c): Schematic illustration of the electronic band structure. The flat band potential would be about 0.10 V below the bottom of the conduction according to previous reports (*Chem. Commun.*, 2012, 48, 11656; *Chem. Commun.*, 2014, 50, 7063; *JACS*, 2014, 136, 14845). (**d**) Schematic diagrams for conduction bands and band gap energies of aUiO and A-aUiO, and the standard electrode potentials for CO_2/HCOOH and H_2O/H_2 (vs the NHE at pH = 7).

Moreover, as revealed by the electron spin resonance (ESR) spectra (recorded at 77 K in nitrogen) (**Supplementary Figure 18c** in the revised SI), under visible irradiation, strong ESR signals with a *g* value of 2.004 can be observed on both aUiO and A-aUiO samples. Such signals, indicating the forming of Zr^{III} species (*Chem. Eur. J.*, 2013, 19, 14279), therefore suggest that the electrons can be transferred from linkers to the Zr–O clusters under visible irradiations.

In addition, we compared the FT-IR spectroscopy for aUiO before and after activation to investigate whether the linkers were oxidized or not during the activation process. As shown in **Fig. R5**, no significant changes in the peaks at 1685 cm^{-1} [which can be ascribed to the $-NH_2$ scissoring vibration (*JACS*, 2017, 139, 9419)] could be found, suggesting that no (significant) oxidation of the linkers could be observed.

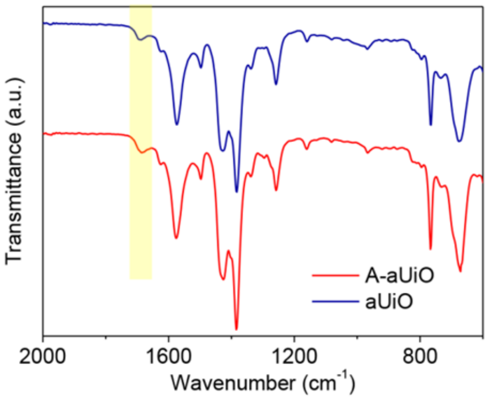

Fig. R5. FT-IR spectra of the as-prepared aUiO and A-aUiO.

We have also revised the manuscript accordingly and inserted **Fig. R4** in the revised SI (**Supplementary Figure 17**).

R2Q6. The bandgap information of photocatalysts and Redox potential of substrates should be provided to better understand the photocatalytic mechanism.

Author Reply:

Thanks for the Reviewer's valuable suggestion.

We have plotted the energy band structure of the photocatalysts (**Fig. R4**) according to the Reviewer's suggestion. Given the conduction band at about −0.6 V vs. NHE. The photo-generated electrons can readily reduce CO_2 into HCOOH (−0.37 V vs. NHE, *Nature*, 2016, 529, 68). We also revised the manuscript accordingly and plot the band structures of the catalysts together with the redox potential of substrates in the revised manuscript (**Lines 10–16, Page 7**, and **Supplementary Figure 17**).

R2Q7. For photocatalytic CO_2RR, IPA was used as sacrificial agent, but why TEOA was used for isotope experiment? Isotope experiment in GMG mode should also be supplemented.

Author Reply:

Thanks for the Reviewer's kind suggestion.

First, we used TEOA as the sacrifice agent in isotope experiment because the peak for certain fragments of IPA (–CH_2CH_2OH, m/z = 45.0) is overlapped with the $H^{12}COO^-$ peak (m/z = 45.2) in ESI-MS profiles.

Second, according to the Reviewer's suggestion, we carried out the isotope experiment in GMG system. As shown in **Fig. R6**, $H^{13}COO^-$ was the only product when $^{13}CO_2$ was used as feed gas, confirming that the formates were generated from photocatalytic reduction of CO_2 exclusively. We also revised the manuscript accordingly (**Lines 6–7, Page 8**) and inserted **Fig. R6** in the revised SI (**Supplementary Figure 37**).

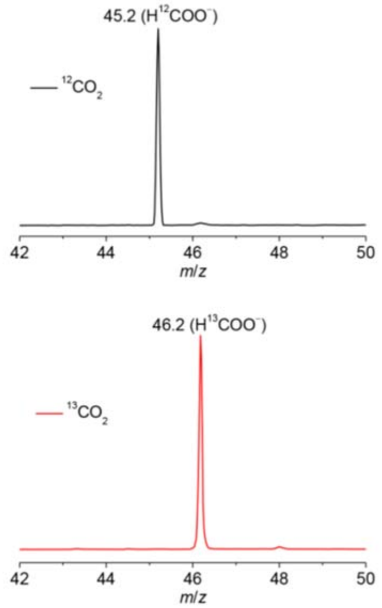

Fig. R6. ESI-MS profiles for the liquid products of photocatalytic CO_2RR on Ir_1/A-aUiO membranes in the GMG mode with $^{12}CO_2$ and $^{13}CO_2$ as gas reactants. During the test, high-pure CO_2 (99.9999%) was humidified by ultrapure water and cycled continuously in the whole system by a gas-recycle-pump so that the CO_2 and water molecules could continuously pass across the membrane. The membrane was irradiated by visible light for 4 hours before ESI-MS tests. The peaks at m/z = 45.2 and 46.2 are assigned to $H^{12}COO^-$ and $H^{13}COO^-$, respectively, suggesting that the formates were generated from photocatalytic CO_2 reduction exclusively.

R2Q8. Wavelength-dependent activity together with QE should be provided.

Author Reply:

Thanks for the Reviewer's valuable suggestion.

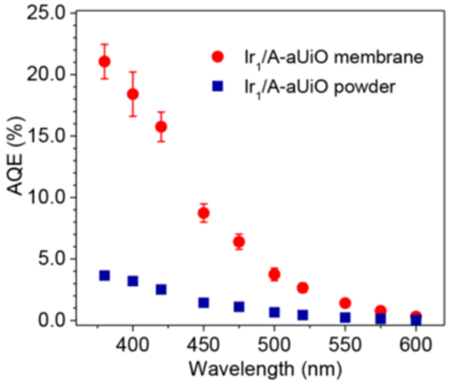

Fig. R7. Wavelength dependence of apparent quantum efficiency (AQE) during photocatalytic CO_2 reduction on Ir_1/A-aUiO powders and membranes.

As shown in **Fig. R7**, we have measured and calculated the wavelength-dependent apparent quantum efficiency (AQE) for photocatalytic CO_2 reduction on Ir_1/A-aUiO powders and membranes. At the wavelength of 420 nm, the Ir_1/A-aUiO membrane exhibited an AQE of 15.76%, which is more than 6 times that showed by Ir_1/A-aUiO particles (2.51%) in the conventional PiS mode.

We have also revised the manuscript accordingly (**Lines 2–6, Page 8**) and inserted **Fig. R7** in the revised SI (**Supplementary Figure 36**).

R2Q9. Authors stressed that Ir SAs well dispersed in *d*-aUiO when Ir loading content is less than 1.4wt%. But increasing the Ir loading to 2.7 wt.%, Ir clusters appeared. For Ir SAs synthesis, annealing process under Ar is used. But for Ir cluster synthesis, H_2/Ar and higher temperature was used. Thus, the conclusion "Lowering the Ir loading to 0.7 wt.% resulted in sparser dispersion of Ir SAs in Ir_1/*d*-aUiO (Figures S6 and S7), while increasing the loading of Ir to 2.7 wt.% led to the formation of *d*-aUiO containing both Ir SAs and small clusters (Ir_x/*d*-aUiO, Figures S8 and S9)" is arbitrary. Experiments under the same annealing condition but different Ir loading amount should be applied to prove the above conclusion.

Author Reply:

Thanks for the Reviewer's kind reminder.

We have revised the sentence of "*Lowering the Ir loading to 0.7 wt.% resulted in sparser dispersion of Ir SAs in Ir_1/d-aUiO, while increasing the loading of Ir to 2.7 wt.% led to the formation of d-aUiO containing both Ir SAs and small clusters (Ir_x/d-aUiO)*" as "*Lowering the Ir loading to 0.7 wt.% resulted in sparser dispersion of Ir SAs in Ir_1/A-aUiO, while increasing the loading of Ir to 2.7 wt.% and annealing the hybrids under reducing atmosphere (H_2/Ar, 1/4 v/v) led to the formation of A-aUiO containing both Ir SAs and small clusters (Ir_x/A-aUiO).*" (**Lines 3–6, Page 5**).

R2Q10. According to this manuscript, aUiO stands for amino functionalized UiO-66 with acetic acid linker on nodes, while *d*-aUiO stands for defect amino functionalized UiO-66 without acetic acid. As perfect aUiO has no acetic acid on Zr6 nodes, but 12 linkers around. Therefore, both aUiO and *d*-aUiO are defect MOFs, and the names of aUiO and *d*-aUiO used here are inappropriate.

Author Reply:

Thanks for the Reviewer's valuable suggestions.

We have modified the name of "*d*-aUiO" as "activated aUiO (A-aUiO)" in the revised manuscript. Hence, the term "aUiO" stands for amino-functionalized UiO-66 with acetates on nodes, while the term "A-aUiO" represents activated amino-functionalized UiO-66 with missing linkers and more defects on the Zr–O nodes.

R2Q11. The description "Ir SAs (1.4 wt.%) could be evenly dispersed in *d*-aUiO matrix to form Ir_1/*d*-aUiO particles, as revealed by the scanning electron microscopy (SEM) image (Figure 1c), …" in MS is not right, as even Ir NPs is hard to be distinguished by SEM.

Author Reply:

Thanks for the Reviewer's kind reminder.

In our original manuscript, we did not mean that SEM itself could reveal the dispersion of Ir SAs but that all the characterizations we used (including SEM, AC-HAADF-STEM, and EDS mapping) could together reveal that Ir SAs could be evenly dispersed in the MOF matrix… To avoid any possible misunderstanding, we have revised this sentence as "*For instance, Ir SAs (1.4 wt.%) could be highly dispersed in A-aUiO matrix to form* Ir_1*/A-aUiO particles, as revealed together by the scanning electron microscopy (SEM) image, high-angle annular dark-field scanning TEM (HAADF-STEM) image and corresponding energy-dispersive X-ray spectroscopy (EDS) maps, as well as aberration-corrected HAADF-STEM (AC-HAADF-STEM) images (Figure 1c–e and Supplementary Figure 4)*" in the revised manuscript (**Lines 17–20, Page 4**).

R2Q12. For Ir_1/*d*-aUiO, Ar atmosphere was used, and for Ir_x/*d*-aUiO, H_2/Ar atmosphere was used. But both Pd_1/*d*-aUiO and Pd_x/*d*-aUiO used Ar atmosphere?

Author Reply:

Thanks for the reviewer's question.

In synthesis, as comparted with Ir, Pd species are more easily to form larger nanoparticles when reduced with H_2. As shown in **Fig. R8**, if we reduced Pd precursors in H_2/Ar atmosphere (like that for Ir_x/A-aUiO), we can get larger Pd nanoparticles. Therefore, to prevent the forming of Pd nanoparticles, we had to use the Ar atmosphere to prepare Pd_x/A-aUiO samples.

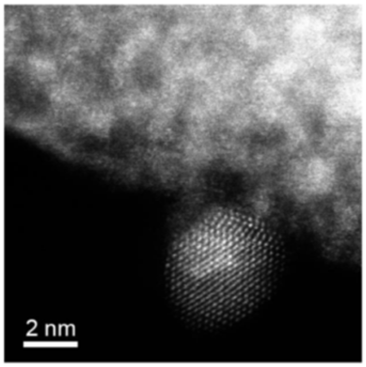

Fig. R8. High-resolution HAADF-STEM image for Pd/A-aUiO samples prepared under H_2/Ar (1/4 v/v) atmosphere, suggesting that the strong reductive atmosphere would promote the formation of larger Pd nanoparticles.

R2Q13. All four peaks should be integrated in Figure S2d and Figure S2e. Only use peak at 8.10 seems not reliable. This peak may ascribe to amino, and seems not reliable. All four peaks should be referred to which H in 2-aminoterephthalic acid. By the way, peak at 1.89 ascribe to acetic acid, and there are three H contributed the area of 0.18. So, the calculation method for the molar ratio of acetate/2-aminoterephthalic acid was wrong in this MS.

Author Reply:

Thanks for the Reviewer's suggestion.

We sincerely apologize for such mistake although it did not change the conclusion of this manuscript. The peak at 8,10 ppm should be assigned to the amino group. Peaks at 8.01, 7.64 and 7.34 ppm should be assigned to 3 protons on benzene ring of 2-aminoterephthalic acid (as shown in **Fig. R9** and **Supplementary Figure 2** in the revised SI). The correct ratio of acetate/2-aminoterephthalic acid should be 0.03 and 0.033 before and after activation, respectively, indicating that most acetate groups could be removed after activation, causing more defects on the Zr–O nodes. We have also revised the SI accordingly.

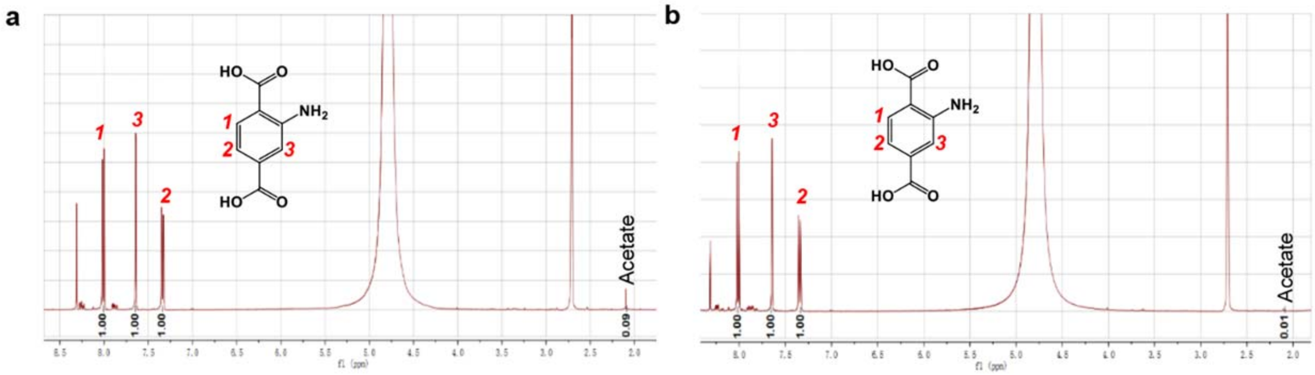

Fig. R9. ^{1}H NMR profiles of digested aUiO (a) and A-aUiO (b) particles, **showing the composition of organic residuals of them.**

R2Q14. Fit parameters of EXAFS are missing.

Author Reply:

Thanks for the Reviewer's suggestion.

The fig parameters of EXAFS are shown in **Table R1**.

Table R1. EXAFS fitting results for as-obtained and recycled catalysts*

Catalysts	Shell	*C.N.*	*R*(Å)	$\sigma^2/\times10^{-3}$(Å^2)	ΔE_0 (eV)
1.4 wt.% Ir_1/A-aUiO	Ir-O	3.8±1.0	2.02±0.01	3.0±1.0	12.2±1.5
	Ir-Ir	–	–	–	–
0.7 wt.% Ir_1/A-aUiO	Ir-O	4.0±1.1	2.04±0.02	3.0±1.0	12.2±1.5
	Ir-Ir	–	–	–	–
Ir_1/A-aUiO (recycled)	Ir-O	4.1±1.0	2.04±0.01	7.7±2.1	8.6±1.8
	Ir-Ir	–	–	–	–
Ir_x/A-aUiO	Ir-O	2.1±0.6	1.99±0.05	4.3±1.4	9.1±1.8
	Ir-Ir	4.3±0.4	2.67±0.02	6.9±1.6	9.1±1.8
Ir_1/aMIL	Ir-O	3.5±0.7	2.02±0.02	3.0±1.0	12.2±1.5
	Ir-Ir	–	–	–	–
Pd_1/A-aUiO	Pd-O	3.7±0.4	2.02±0.02	2.8±1.1	4.6±1.6
	Pd-Pd	–	–	–	–
Pd_x/A-aUiO	Pd-O	2.1±0.3	2.06±0.01	5.0±1.6	0.9±1.5
	Pd-Pd	3.4±0.4	2.72±0.01	4.3±1.6	2.6±1.8

*CN: coordination number
σ^2: Debye-Waller factor (a measure of thermal and static disorder in absorber-scatterer distances)
ΔE_0 is the edge energy shift (the difference between the zero kinetic energy value of the sample and that of the theoretical model).

We have also supplemented these fit parameters in the revised SI (**Supplementary Table 1**).

R2Q15. No scale bar for Figure S1. Figure S4C was missing. XPD in Figure S33 should be XRD.

Author Reply:

Thanks for the Reviewer's kind reminder.

We should apologize for such typos and errors. We have corrected such errors (**Supplementary Figures 1, 4c and 40**) in the revised SI.

Reviewer #3:

This study looks at the fabrication and testing of supported MOF membranes for CO_2 and O_2 photoreduction. A particular aspect of the material is that they contain single metal atoms dispersed throughout the MOF structure to enhance the photocatalytic response. There are lots of interesting analyses carried out in this work, which is very much commended. The downside of this is that it can be difficult to extract the relevant analyses and confirm that they indeed support the claims of the study. Ultimately, there is a sense that no much new information is provided by the study presented here, especially with respect to why SA/MOF are possibly better.

Author Reply:

Thanks for the Reviewer's positive comments for our work.

To further improve the quality of our manuscript as well as address your concerns, we have made careful revisions according to each comment of the Reviewers. We hope that the revised manuscript can address the concerns from the Reviewers and can meet the high standard required for publication in *Nature Commun*.

R3Q1. The fact that gas-solid set-up is improved compared to gas-liquid set-up is expected due to the limited dissolution of CO_2 in water. This explains the increasing number of gas-solid CO_2 photoreduction studies being reported in the literature. Hence, it is not clear what new knowledge is generated here. For instance, how does Figure 3a-d bring new insights for the community to use going forward? We note that the use of gas-solid systems (incl. membrane-based gas solid systems) for CO_2 photoreduction is not cited in the introduction.

Author Reply:

Thanks for the Reviewer's comments.

The gas-permeable membranes consisting of naturally porous MOF particles can allow the gaseous substance (e.g., CO_2, O_2, H_2O vapor) to be diffused through the pores and channels of the MOF crystals with high flux. Theoretically, all most all the catalytic sites located on the Zr–O nodes (e.g., SAs, clusters, etc.) could be accessible to the gas molecules diffused through the MOF pores. Therefore, our catalyst membrane can be considered as an assembly of numerous regularly arranged nanoreactors, each of which has its own photocatalytic unit consisting of ligand (sensitizer), Zr–O node (supporter), and metal SA (active center), as shown in **Figure. 3b** in our manuscript.

In contrast, previously reported solid films made of close-packed nanocrystals (e.g., WO_3 nanosheets, *Joule*, 2018, 2, 1004; bismuth-based perovskite nanocrystals, *JACS*, 2019, 141, 20434), as well as the TiO_2/ZnO films presented in our manuscript as control samples, are not that gas permeable and only a small portion of surface sites that are not covered by the close-packed particles are accessible to the gaseous reactants (as illustrated in **Figure. 3c** in our manuscript).

Therefore, although both reaction interfaces can be categorized into the gas-solid interfaces that can avoid the sluggish diffusion of low-concentration gas molecules in aqueous solutions, our gas-porous membrane-gas reaction interface exhibit even more advantages in reaction efficiency. As compared to the Ir_1/A-aUiO particles dispersed in aqueous solutions, the Ir_1/ A-aUiO membranes could significantly promote the photocatalytic CO_2-to-HCOOH conversion,

increasing the reaction rate from 0.51 mmol g_{cat}^{-1} h^{-1} to 3.38 mmol g_{cat}^{-1} h^{-1} (**Figure. 3d**) and the apparent quantum efficiency (AQE, at 420 nm) from 2.51% to 15.76% (**Supplementary Figure 36** in the revised SI). In contrast, the solid films of Ir/TiO_2 and Ir/ZnO, with poor gas permeability (**Supplementary Figure 44** in the revised SI) and less density of accessible catalytic sites, showed much minor improvements in reaction rates (**Supplementary Figure 43** in the revised SI).

Another advantage for such MOF membranes is that we can readily construct cascaded reactors consisting of different types of metal/MOF particles through a layer-by-layer way for specific shape-selective and/or tandem catalytic reactions, which is difficult to achieve in conventional planar solid films. Related work is still ongoing in our lab now.

In sum, as compared to previous gas-solid interfaces made by stacking solid nanoparticles together closely and randomly, our metal/MOF membranes with porous structures and regular and accessible photocatalytic ligand-node-metal (SAs) units represent a novel kind of gas-solid reaction interface with exceptionally large specific surface area (e.g., 812.5 m^2 g^{-1} for Ir_1/A-aUiO). Unlike the planar solid films with limited gas permeability and transient contact between the gas molecules and the surface active sites, such catalysts membrane would serve as chromatography-column-like reactor which allows the gaseous substances to be diffused though the inside nanoreactors with relatively long retention time and high throughput (**Figure 3b and Supplementary Figure 44**). As a result, such gas-permeable membranes can not only catalyze a series of photocatalytic gas fixation reaction with high activity and selectivity, but also hold great possibility for future selective and/to tandem catalysis research.

In addition, as suggested by the Reviewer, we have revised the introduction part of the manuscript to make it more concise and straightforward (**Lines 25–31, Page 2**).

We hope our additional experiments and discussions can address the Reviewer's concern and our revised manuscript can meet the high standard required for publication in *Nature Commun*.

R3Q2. The "unprecedented activity" and the comparison table (Table S1) do not mean much and cannot be used for comparison since this rate depends on the test conditions and reactor set-up. Following guidelines in the field, the authors should instead report apparent quantum yield or quantum efficiency. I suggest not reporting production rate in the abstract. On that note, one needs to clarify whether the "cat" in "g_{cat}" is the whole material (incl membrane), the MOF or the supported metal.

Author Reply:

Thanks for the Reviewer's valuable suggestions.

As suggested by the reviewer, we have revised the abstract of the manuscript and deleted the related statements about HCOOH production rate. We also calculated the wavelength-dependent apparent quantum efficiency (AQE) (*see* ***Fig. R7*** *in our reply to* ***R2Q8***).

In addition, the term "cat" in "g_{cat}" represents the "metal/MOF" catalysts while the membrane is not included. We have also revised our manuscript accordingly (**Lines 23, 24, 26 in Page 1; Lines 15–16 in Page 3; Line 1 in Page 8**).

R3Q3. Studies on single atom supported MOF for photocatalysis (particularly solar fuels production) have already been reported. These references (or a selection of them) should be included and the link to prior research in this field must be shown, as well as how this work provides new insight compared to what has been done before.

Author Reply:

Thanks for the Reviewer's kind suggestions.

We have revised the introduction part of the manuscript according to the Reviewer's suggestions (*see also our reply to* ***R3Q1***). We summarized representative work on solar fuels production with single-atoms/MOF catalysts and compared previous achievements with our work to demonstrate the new strategy and insight we would like to share with the community (**Lines 10–16, Page 2**).

R3Q4. A comparison between SA/MOF and NP/MOF composites is made, which is indeed necessary to show the role of SAs. The EXAFs and XPS nicely confirm the contrasting patterns of NPs and SAs. However, it is not clear what the amount of NPs is compared to the amount of SAs (unless I missed this information). If the amount is different, then how can a comparison be done? What would confirm that the improvement comes from the SA nature of the metal as opposed to an optimized metal content? Overall, the characterisation of all the samples (incl the ones containing NPs) should be conducted at the same level of depth and presented (e.g. elemental analysis, porosity, DRS UV vis, PL, etc…). Otherwise, one is presented with only a partial view of the parameters that can influence the photocatalysis.

Author Reply:

Thanks for the reviewer's kind suggestions.

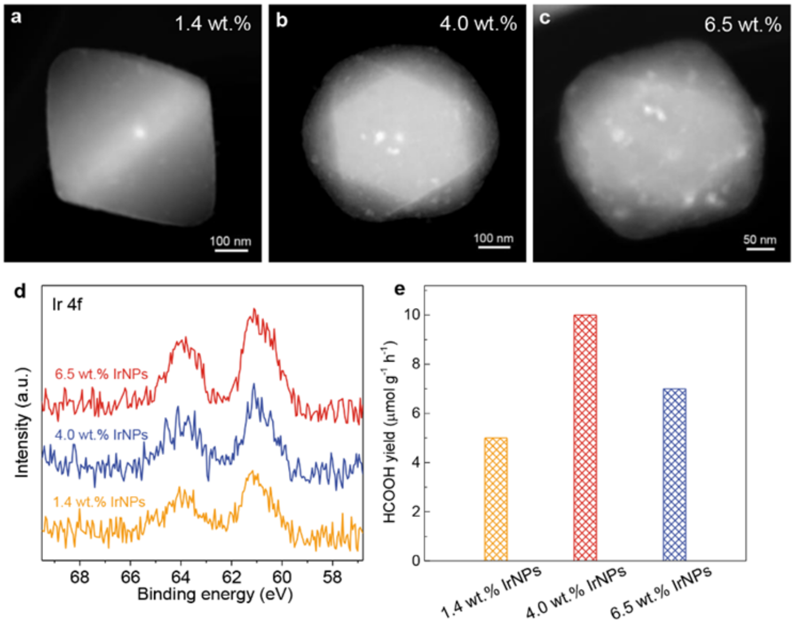

Fig. R10. Characterizations and photocatalytic CO_2RR performances on IrNPs/A-aUiO with different Ir loadings. (**a–c**) HAADF-STEM images of IrNPs/A-aUiO with the Ir mass loading of (**a**) 1.4 %, (**b**) 4.0 %, and (**c**) 6.5 %. (**d**) Ir 4*f* core level XPS spectra for IrNPs/A-aUiO with different metal loading, showing that the Ir NPs were all mainly in metallic

state. (**e**) HCOOH yields on IrNPs/A-aUiO powders (PiG mode) with different Ir loadings under similar reaction conditions.

As stated in our original manuscript and SI, the mass loading for Ir is 4.0 % in IrNPs/A-aUiO, while 1.4 % and 0.7 % in two kinds of IrSAs/A-aUiO samples. In our original manuscript, we used 4.0 wt.% IrNPs/A-aUiO as control sample in the manuscript due to its better catalytic performances than other IrNPs/A-aUiO samples. In fact, we had already prepared IrNPs/A-aUiO saples with other Ir mass loadings. As shown in **Fig. R10**, the density of Ir NPs inside the A-aUiO particles increased with the mass loading of Ir. However, the 4.0 wt.% IrNPs/A-aUiO samples exhibited significantly higher HCOOH yield (10 μmol g^{-1} h^{-1}) than that of 1.4 wt.% IrNPs/A-aUiO (5 μmol g^{-1} h^{-1}) and 6.5 wt.% IrNPs/A-aUiO (7 μmol g^{-1} h^{-1}) samples. That is, the HCOOH yield on 1.4 wt.% IrSAs/A-aUiO (the main sample in our manuscript) and 1.4 wt.% IrNPs/A-aUiO was about 510 μmol g^{-1} h^{-1} and 5 μmol g^{-1} h^{-1}, respectively, suggesting that the activity of the former is 2 orders of magnitude higher than the latter.

In our original manuscript, we showed a head-to-head comparison for the Zr 3d XPS for different Ir/A-aUiO samples, showing that the metal-support interaction was stronger in the IrSAs/A-aUiO samples than that in Ir_x/A-aUiO and IrNPs/A-aUiO samples (**Supplementary Figure 27** in the revised SI). More comparisons, including photoluminescence (PL) spectra, electrochemical impedance spectroscopy (EIS) spectra, and photocurrent-time curves (**Supplementary Figure 20** in the revised SI), further revealed that decoration of Ir species (*esp.* Ir SAs) could effectively boost the separation of photoexcited electron-hole pairs in A-aUiO.

Therefore, the systematic comparison between the IrSAs/A-aUiO and IrNPs/A-aUiO samples suggest that the nature of the Ir SAs (e.g., the strong metal-support interactions, the unique electronic structures, etc.) would play a major role in the improvement of the catalytic performances of Ir/A-aUiO catalysts.

We hope that our additional data can address the Reviewer's concerns and we've also revised our manuscript accordingly (**Lines 6–9 in Page 3 and Lines 26–30 in Page 5; Supplementary Figures 20 and 23**)

R3Q5. The "synergistic diffusion and adsorption" do not seem to be demonstrated in the study, unless I have missed it. Regarding diffusion, the case is made between PiS and GMG in Figure 3 but the role of the material is not shown. Also, the faster diffusion in the SAF/MOF case compared to the solid film case seems to be speculated not demonstrated. The adsorption aspect is alluded to in Figure S26 and S27, however these has not been any specific adsorption uptake carried out to quantify the amount adsorbed and support the claims related to "higher local concentration of CO_2".

Author Reply:

Thanks for the Reviewer's kind suggestions.

First, the differences for gas diffusion through MOF membranes (e.g., Ir_1/A-aUiO and Ir_1/aMIL) and solid films (e.g., Ir/TiO_2 and Ir/ZnO) can be found in **Supplementary Figure 37** in the original SI **(Supplementary Figure 44** in the revised SI**)**. At 5 kPa, the flow rate of CO_2 through the Ir_1/A-aUiO, Ir_1/aMIL membranes and Ir/TiO_2, Ir/ZnO films was 39.2, 38.2 sccm, and 3.2, 1.0 sccm, respectively. At a higher pressure of 20 kPa, the flow rate was 172, 147, 10.9, and 8.7

sccm, respectively. That is, owning to the naturally porous structure of the MOF crystals, the diffusion of gas through the porous membranes would be much faster than that through the solid films.

Second, the increase in concentration of CO_2 in the GMG mode (CO_2 gas with saturated water vapor) as compared with the PiS mode (statured aqueous solution of CO_2) can be calculated with standard data. As we had discussed in the manuscript (**Lines 27–28, Page 7**), at 25 °C, the molecular ratio of CO_2/H_2O in the vicinity of each catalytic centers can be increased by 4 orders of magnitude from 1/1600 (saturated CO_2 solutions, PiS) to ~30/1 (CO_2 gas with saturated H_2O steam, GMG) (*Nat. Catal*, 2018, 1, 592). The increase of local CO_2 concentration and the reverse of CO_2/H_2O ratio in the GMG mode would thus dramatically enhance the CO_2RR activity.

Third, regarding the adsorption of CO_2 by the MOF membrane and the solid film, we have measured the volumetric CO_2 adsorption according to the Reviewer's suggestion. As shown in **Fig. R11**, the Ir_1/A-aUiO/PTFE (Ir_1/A-aUiO/ PTFE = 0.6 wt.%) membrane exhibited significantly higher CO_2 uptake than the Ir/TiO_2/PTFE (Ir/TiO_2/ PTFE = 1.0 wt.%) film, demonstrating the much stronger CO_2 adsorption capability of the MOF membranes.

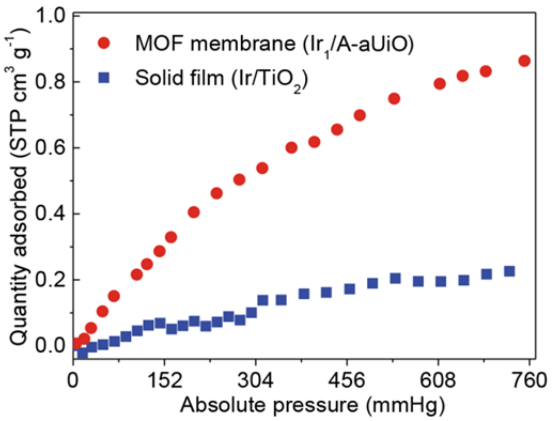

Fig. R11. CO_2 adsorption isotherms of MOF (Ir_1/A-aUiO) membrane and solid (Ir/TiO_2) membrane. The quantity adsorbed (y axis) was normalized to the mass of Ir_1/A-aUiO/PTFE and Ir/TiO_2/PTFE membranes, respectively.

We hope our experiment results and discussions could address the Reviewer's concerns.

R3Q6. The authors use the term "breathable". What does it actually mean?

Author Reply:

Thanks for the reviewer's question.

We used the term "breathable" to highlight that the membranes, resembling the green leaves, were porous and gas-permeable so that gas reactants (e.g., CO_2, O_2, etc.) can be diffused through the membranes with high flux.

To avoid possible ambiguity, we revised the manuscript accordingly by replacing "breathable" with "gas-permeable" and/or other terms (**Line 9, Page 5**).

We hope our explanation and revision could address the Reviewer's concerns.

R3Q7. The authors claim high porosity of the materials, however the results of their N_2 sorption analyses are not presented (unless I missed it).

Author Reply:

Thanks for the Reviewer's kind reminder.

We had measured the N_2 sorption isotherms for aUiO, A-aUiO, Ir_1/A-aUiO (1.4 wt.%), Ir_x/A-aUiO (2.7 wt.%) and IrNPs/A-aUiO (4.0 wt.%) catalysts (**Fig. R12**). Now we have revised the manuscript and showed these isotherms in the updated SI (**Supplementary Figure 2**).

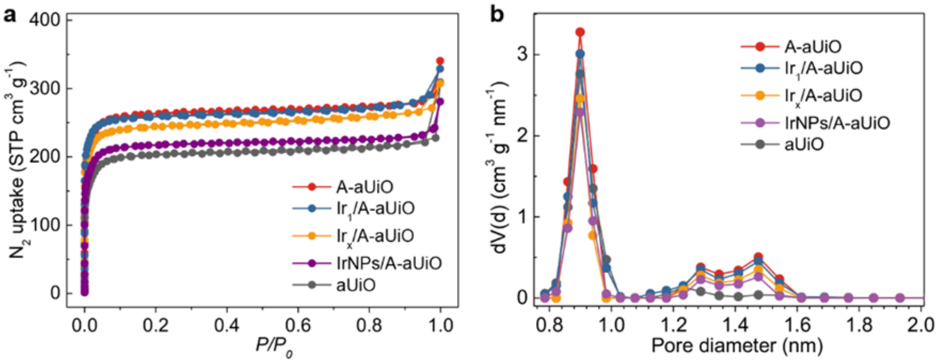

Fig. R12. N_2 adsorption-desorption isotherms (a) and pore size distributions (b) for different as-obtained catalysts: aUiO, A-aUiO, Ir_1/A-aUiO (1.4 wt.%), Ir_x/A-aUiO (2.7 wt.%) and IrNPs/A-aUiO (4.0 wt.%) catalysts.

R3Q8. The authors indicate a selectivity near 100% but the nature of the other products identified is not presented. One needs to know the products distribution (incl. liquid and gaseous products).

Author Reply:

Thanks for the Reviewer's kind suggestion.

Fig. R13 shows typical product distributions for photocatalytic CO_2RR on 0.7 wt.% Ir_1/A-aUiO, 1.4 wt.% Ir_1/A-aUiO, 2.7 wt.% Ir_x/A-aUiO and 4.0 wt.% IrNPs/A-aUiO in the conventional PiS mode under visible light irradiation (> 420 nm). For Ir_1/A-aUiO, the main products were HCOOH and CO with the yield of 234.2 and 9.0 μmol $g^{-1}h^{-1}$ on (96.3% selectivity for HCOOH), respectively. For 1.4 wt% Ir_1/A-UiO, the main products were HCOOH and CO, with the yield of 510.2 and 5.4 μmol $g^{-1}h^{-1}$ on (99.0% selectivity for HCOOH), respectively. On Ir_x/A-aUiO catalysts, the main products were HCOOH, H_2, and CO, with the yield of 159.6, 37.4, and 3.0 μmol $g^{-1}h^{-1}$ (79.8% selectivity for HCOOH). For 4.0% IrNPs/A-aUiO, the main products were H_2, CO, and HCOOH, with the yield of 35.9, 14.8 and 10.0 μmol $g^{-1}h^{-1}$ (16.5% selectivity for HCOOH), respectively.

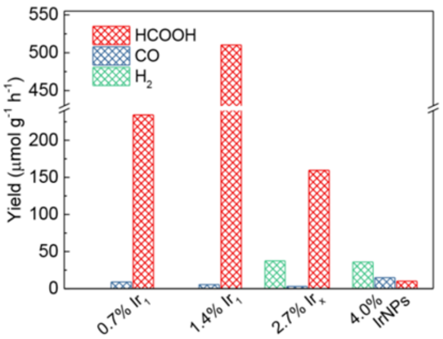

Fig. R13. Photocatalytic reduction yields for different products on Ir_1/A-aUiO, Ir_x/A-aUiO and IrNPs/A-aUiO catalysts.

We hope our additional data and discussions could address the Reviewer's concerns. We have also revised our manuscript accordingly (**Lines 26–30, Page 5; Supplementary Figure 24**).

R3Q9. The optoelectronic properties of the materials are not investigated in much depth. Only some of the UV vis spectra are shown. The band diagrams of the materials are not presented.

Author Reply:

Thanks for the Reviewer's valuable suggestion.

As suggested by both **Reviewers 2 & 3** (*see also our reply to **R2Q5***), we have measured the UV-vis diffusive reflectance spectra and Mott-Schottky (M-S) curves for aUiO and A-aUiO. As shown in **Fig. R4a**, the band gap for aUiO is about 2.82 and 2.47 eV before and after activation, respectively. The Mott–Schottky measurements conducted at four different frequencies suggest that the conduction band position was about −0.91 V and –0.60 vs. NHE for aUiO before and after activation, respectively (**Fig. 4b, c**). The valence band (i.e., HOMO) potential for aUiO before and after activation is then calculated to be 1.91 V and 1.87 V vs. NHE, respectively.

We hope our additional data and discussions could address the Reviewer's concerns. We have also revised our manuscript accordingly (**Lines 10–16, Page 7; Supplementary Figure 17**).

R3Q10. Having an understanding of the photoreaction mechanisms would bring novelty to the work. The optoelectronic analysis mentioned above along with the in-situ FTIR analyses that the authors initiated would be extremely useful in this regard.

Author Reply:

Thanks for the Reviewer's valuable suggestion.

As replied in ***R2Q5*** and ***R3Q9***, we have measured the band structures of the A-aUiO (**Fig. R4** and **Supplementary Figure 17** in the revised SI). The photo-generated electrons in the conduction band of A-aUiO (−0.6 V vs. NHE) are negative enough to reduce CO_2 into HCOOH (−0.37 V vs. NHE) (*Nature*, 2016, 529, 68).

In addition, as shown in **Supplementary Figure 29** in the revised SI, FTIR peaks (at 1497 and 1433 cm^{-1}) of the surface carbonic acid species (*JACS*, 2019, 141, 2911; *Chem. Soc. Rev.*, 2017, 46, 1785), the intermediate species for

formic acid evolution (*JACS*, 2019, 141, 2911), were immediately observed when humidified CO_2 was fed into the reactors containing Ir_1/A-aUiO or A-aUiO catalysts in dark, indicating the adsorption of CO_2 by the hydroxyls on the Zr–O nodes of A-aUiO (*J. Phys. Chem. C.*, 2015, 119, 6980). After irradiated catalysts by visible light, new infrared peaks (at 2920, 2850 and 1576 cm^{-1}) were observed on the Ir_1/A-aUiO catalyst (**Supplementary Figure 30** in the revised SI), suggesting the formation of HCOO* species upon irradiation (*J. Catal.*, 1992, 136, 493; *Catal. Lett.*, 2010, 137, 239). To the contrary, no significant HCOO* peaks could be found in the FTIR spectra for the bare A-aUiO catalyst, indicating the vital role of Ir_1 species. We hope our additional data and discussions could address the Reviewer's concerns.

R3Q11. "Ir clusters and nanoparticles, the Ir SAs were more electron-deficient as revealed by X-ray absorption near-edge structure (XANES) and X-ray photoelectron spectroscopy (XPS) analysis (Figures 2e and S24). Such differences in electronic structures may thereby result in their different catalytic reactivity." Why would that be the case? Wouldn't electron-rich sites be favourable for a reduction reaction? Again, we are missing an understanding of the distinct behaviour of SA/MOF vs non SA/MOF.

Author Reply:

Thanks for the reviewer's valuable inquiry and suggestion.

We used the term of "electron-deficient" to describe that these Ir SAs were of relatively higher valances (i.e., positively charged) as compared with metallic nanoparticles (valence = 0) at the rest mode (without irradiation). According to previous reports (*JACS*, 2018, 140, 16042; *JACS*, 2019, 141, 7615; *Science*, 2019, 364, 1091), such positively charged metal single atoms exhibit much stronger bonding with CO_2 molecules than metallic nanoparticles. The strengthened CO_2 adsorption could thereby promote CO_2 activation on metal single atom sites. As a result, in photocatalysis, upon the arrival of electrons from the sensitizers, such single-atomic active centers can thereby boost photocatalysis CO_2RR effectively (**Figure 2f** in the original manuscript).

We hope our additional discussions could address the Reviewer's concerns. We have also revised our manuscript accordingly (**Lines 26–30 in Page 5 and Lines 3–8 in Page 6**).

R3Q12. $^{13}CO_2$ tests and other control tests are missing for GMG (and also for the O_2 reduction study).

Author Reply:

Thanks for the Reviewer's kind suggestion.

We have carried out the $^{13}CO_2$ tests in the GMG mode *(see also our reply to **R2Q7**)*. As shown in **Fig. R6**, $H^{13}COO^-$ was the only product when $^{13}CO_2$ was used as feed gas, confirming that the formates were generated from photocatalytic CO_2RR exclusively. We also revised the manuscript accordingly (**Supplementary Figure 37** in revised SI).

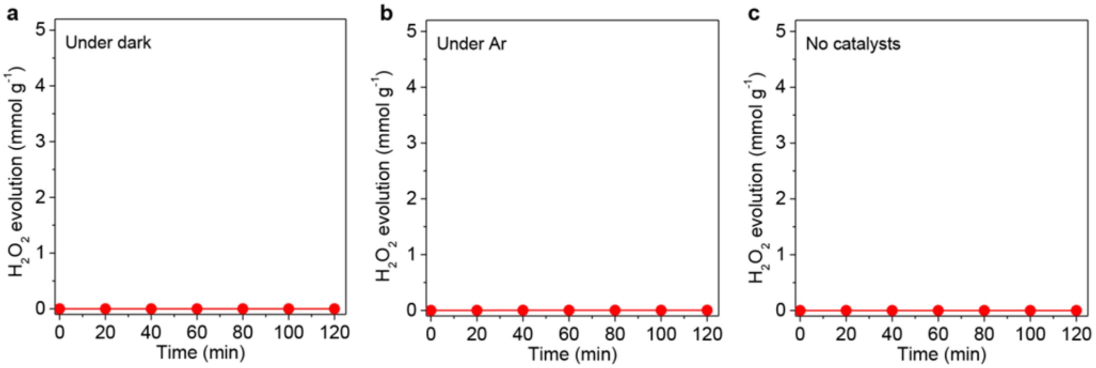

Fig. R14. Blank experiments for photocatalytic O_2 reduction on Pd_1/A-aUiO powders. No H_2O_2 generation could be detected in experiments without (**a**) light irradiation, (**b**) O_2 feed or (**c**) catalysts (i.e., Pd_1/A-aUiO).

In addition, we've ordered $^{18}O_2$ from several vendors, but, unfortunately, no one could deliver high-purity $^{18}O_2$ from abroad to China in 4 months from now due to the breakout of COVID-19. However, we would like to submit our revised manuscript in a timely manner. Therefore, we managed to perform a series of additional control experiments to make sure that the detected hydrogen peroxide is produced by reducing O_2. As shown in **Fig R14**, no H_2O_2 production could be detected in experiments without light irradiation, O_2 feed, or catalysts (i.e., Pd_1/A-aUiO), confirming that the detected H_2O_2 was produced by photo-catalytically reducing O_2 exclusively.

We hope our additional data and discussions could address the Reviewer's concerns. We have also revised our manuscript accordingly (**Lines 16–18, Page 10; Supplementary Figure 49**).

R3Q13. Post-photoreduction characterisation of the materials are missing.

Author Reply:

Thanks for the reviewer's kind suggestion.

We have performed post-reaction characterizations for the photocatalysts (i.e., Ir_1/A-aUiO) after long-term (100 h) operations (*see also our reply to **R2Q4***). As shown in **Fig. R3**, the chemical structures of the Ir_1/A-aUiO catalysts are very stable during the long-term reaction.

We hope our additional data and discussions could address the Reviewer's concerns. We have also revised our manuscript accordingly (**Lines 16–18, Page 8; Supplementary Figure 38**).

R3Q14. The products of the oxidation reaction are not indicated.

Author Reply:

Thanks for the Reviewer's kind suggestion.

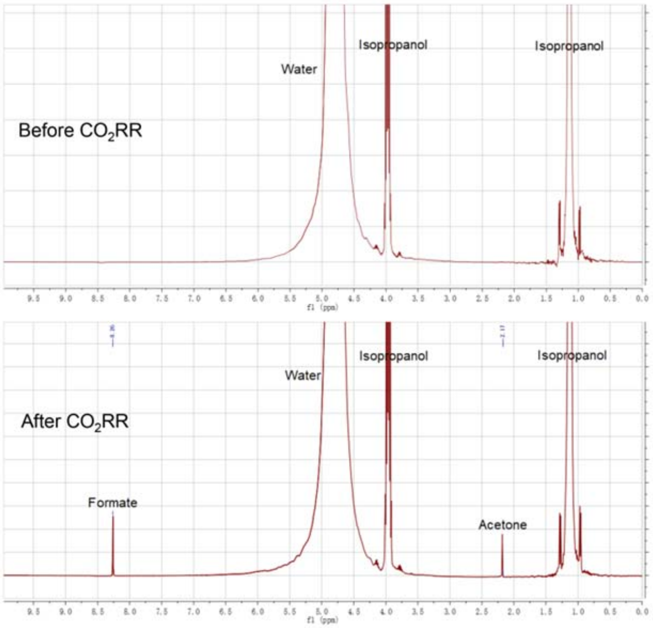

Fig. R15. The ^1H NMR spectra for the liquid products in the photocatalytic CO_2RR by using isopropanol as sacrificial agent. The peaks at 8.26 and 2.17 ppm can be assigned to $HCOO^-$ (reduction product of CO_2) and acetone (oxidation product of IPA), respectively.

We monitored the oxidation products of IPA with nuclear magnetic resonance (NMR) test. As shown in **Fig. R15**, after photocatalytic CO_2RR, two (and only two) new peaks emerged in the NMR spectra for the resultant solution. The one at 8.26 ppm is assigned to formate, which is the products of the reduction of CO_2; the other at 2.17 ppm can be assigned to acetone, revealing the acetone is the product of the oxidation reaction.

We hope our additional data and discussions could address the Reviewer's concerns. We have also revised our manuscript accordingly (**Lines 14–16, Page 7; Supplementary Figure 31**).

R3Q15. Figure 1 and Figures S4, S6, S8: why are some dots highlighted and not others? What makes one identify them as Ir/Pd?

Author Reply 15:

Thanks for the Reviewer's questions.

The identification of the dispersion of the Ir/Pd was realized by the collaboration of AC-HAADF-STEM, XAFS, and XPS, etc. Form the AC-HAADF-STEM images we can distinguish different metal atoms from their different Z-contrast that would be approximately proportional to Z^2 (e.g., Z_{Ir} = 192, Z_{Pd} = 106, and Z_{Zr} = 91). In our original manuscript, we would like to emphasize the existence of highly dispersed single noble metal atoms and hardly can any noble metal nanoparticles (which would be very bright and large spots in the HAADF-STEM images) could be found.

As suggested by the Reviewer, we have removed all the circles in the revised manuscript to avoid possible ambiguations.

REVIEWER COMMENTS

Reviewer #2 (Remarks to the Author):

All my concerns have been addressed properly.

Reviewer #3 (Remarks to the Author):

I thank the authors for the efforts made and new insights brought by the revised manuscript. Several points have been addressed. Others are remaining (see details below). The clarity of the manuscript has not really improved, as the structure was maintained and further analyses were added.

R3Q1: I thank the authors for the points made. I maintain though that there are already studies on porous materials (incl. MOFs) used for photoreduction of CO2. One reason for using such material is precisely the better access/diffusion of CO2 to the catalytic site.
Based on this, the maybe more novel aspect of the work presented by the authors is the functionalisation with SA but not the use of porous photocatalyst. The authors should review their claims in the introduction and results and discussion sections accordingly.
doi.org/10.1002/adma.201400428
doi.org/10.1016/j.apcatb.2015.10.037
doi.org/10.1039/C6TA04620G
10.1002/cssc.202002824
doi.org/10.1039/C9GC03131F
R3Q2: Thank you. Please provide the details of how AQE was calculated as its definition has been used differently by different groups.
R3Q3: Thank you.
R3Q4: Thank you.
R3Q5: Thank you for the clarification. The flow rate measured and related trends for the different material may or may not entirely correlate to the diffusion to the catalytic site. A priori, one would expect that they will but the two metrics are different so the term diffusion should probably used more carefully.
If I understand correctly, the "higher local concentration of CO2" is not demonstrated by the authors but supported by a separate study. This should be made clearer.
R3Q6: Thank you.
R3Q7: Thank you.
R3Q8: Thank you.
R3Q9: Thank you. The use of Mott-Schottky for identification of band edges is disputed for such materials (10.1039/C9TA09569A) though it continues to be used a lot for MOF-based materials. XPS/UPS techniques tend to be more appropriate.
When representing the electronic diagram, one should also indicate the redox potential for the water and isopropanol redox couples so the thermodynamic drive for the oxidation reaction (for CO2) or reduction (for O2) can be visualised.
R3Q10: Thank you.
R3Q11: This is interesting. Thank you.
R3Q12: Thank you.
R3Q13: Thank you. I suggest adding the porosity measurement after testing as is advised to demonstrate stability of MOF-based structures (besides XRD).
R3Q12: Thank you. The answer from the authors seem only partial. The authors tested CO2 reduction using water vapour or isopropanol vapour. When using water, what is the oxidation product observed?
R3Q15: Thank you.

Response to Reviewers

Reviewer #3:

I thank the authors for the efforts made and new insights brought by the revised manuscript. Several points have been addressed. Others are remaining (see details below). The clarity of the manuscript has not really improved, as the structure was maintained and further analyses were added.

Author Reply:

Thanks for the Reviewer's positive comments and support for our work.

We have made careful revisions according to the Reviewers' suggestions to improve the quality of our manuscript. We hope that the revised manuscript can address the Reviewers' concerns and can be accepted for publication in *Nat. Commun*.

R3Q1. I thank the authors for the points made. I maintain though that there are already studies on porous materials (incl. MOFs) used for photoreduction of CO_2. One reason for using such material is precisely the better access/diffusion of CO_2 to the catalytic site. Based on this, the maybe more novel aspect of the work presented by the authors is the functionalisation with SA but not the use of porous photocatalyst. The authors should review their claims in the introduction and results and discussion sections accordingly.

doi.org/10.1002/adma.201400428

doi.org/10.1016/j.apcatb.2015.10.037

doi.org/10.1039/C6TA04620G

10.1002/cssc.202002824

doi.org/10.1039/C9GC03131F

Author Reply:

Thanks for the Reviewer's kind suggestions.

We have carefully reviewed and cited the related references (*Adv. Mater.*, 2014, 26, 4783; *Appl. Catal. B*, 2016, 183, 47; *J. Mater. Chem. A*, 2016, 4, 15126; *ChemSusChem*, 2021, 10.1002/cssc.202002824; and *Green Chem.*, 2019, 21, 6606) provided by the Reviewer and further refined the innovations of our work according to the Reviewer's valuable suggestions.

Three main modifications have been updated in the introduction part of the manuscript (**Lines 25–29, Page 2 and Lines 1–6, Page 3**): (i) we indicated that the MOFs materials had been investigated in photocatalytic CO_2 reduction, showing their advantages in large surface areas, tunable functionalities, and high porosity; (ii) we emphasized the advantages of anchoring CO_2RR-active single atoms (SA) on the regularly arranged metal oxide nodes of MOFs, which creates numerous interconnected nanoreactors with catalytic centres whose reactivity can be rationally modified by the strong metal-substrate interactions; (iii) we highlighted the advantages of our gas-membrane-gas (GMG) interface design based on the porous MOFs/PTFE membranes where the gaseous reactants are forced to pass through the MOFs pores (i.e., the "interconnected nanoreactors") with high-throughput. In comparison, in the reported conventional MOFs-based gas-solid system in which

solid MOFs powders were stacked in humid CO_2 gas, the simultaneous diffusion of the gaseous reagents into the MOFs pores would be much slower, limiting the reaction efficiency severely (*please see also **Fig. R1** in our reply to **R3Q5** below*).

We hope our modifications can address the Reviewer's concern and our revised manuscript can meet the high standard required for publication in *Nat. Commun*.

R3Q2. Thank you. Please provide the details of how AQE was calculated as its definition has been used differently by different groups.

Author Reply:

Thanks for the Reviewer's kind suggestion.

The calculation of the AQE followed the standard procedure of photocatalytic reactions. In detail, the AQE was obtained by performing CO_2RR under monochromatic light using appropriate bandpass filters at different wavelengths (e.g., 380, 400, 420, 450, 475, 500, 520, 550, 575, and 600 nm). The power intensity of the monochromatic light was measured using an optical power meter (CEL-NP2000, Beijing China Education Au-light Company Limited, China). The calculation of AQE was based on:

$$\mathrm{AQE} = \frac{N_{\mathrm{reacted}}}{N_{\mathrm{incident}}} \times 100\%.$$

In the above equation, N_{reacted} and N_{incident} are the number of the reacted and incident photons, respectively, which can be obtained from:

$$N_{\mathrm{reacted}} = 2 \times N_{\mathrm{A}} \times n_{\mathrm{HCOOH}}, \text{ and}$$

$$N_{\mathrm{incident}} = \frac{Pt}{h\upsilon} = \frac{IS t\lambda}{hc},$$

where N_A is the Avogadro constant (6.022×10^{23} mol^{-1}), n_{HCOOH} is the amount of the produced HCOOH molecules (in mol), P is the light power (W), t is the illumination time (s), h is the Planck constant (6.626×10^{-34} J s), v is light frequency (Hz), I is the light intensity (W cm^{-2}), S is the irradiated area (cm^2), λ is wavelength of monochromatic light (m), and c is the speed of light in free space (3.0×10^8 m s^{-1}).

We have also revised the SI accordingly and inserted the test methods in the experimental section.

R3Q5. Thank you for the clarification. The flow rate measured and related trends for the different material may or may not entirely correlate to the diffusion to the catalytic site. A priori, one would expect that they will but the two metrics are different so the term diffusion should probably used more carefully.

If I understand correctly, the "higher local concentration of CO_2" is not demonstrated by the authors but supported by a separate study. This should be made clearer.

Author Reply:

Thanks for the Reviewer's kind reminder.

We agree with the Reviewer that the flow rate measured and related trends cannot be used as the direct evidence to reveal the CO_2 diffusion efficiency and the term diffusion should be used more carefully. In CO_2RR process, the local concentrations of CO_2 in the vicinity of each SAs within the MOFs pores would be very difficult to obtain. Fortunately, according to previous reports (e.g., *Science*, 2020, 367, 661; *Nat. Catal.*, 2018, 1, 592), the strongly related trends of reaction activity with CO_2 flow rates (**Fig. 3d** in the revised MS) and CO_2 concentration (in CO_2/Ar mixtures, **Fig. 3e** in the revised MS) could effectively reveal that the CO_2RR reactivity is (at least partially) limited by the CO_2 diffusion efficiency.

Moreover, to better demonstrate the advantages of the GMG interface design, we performed further CO_2RR experiments with the as-prepared SA/MOF membranes in the conventional gas-solid system (e.g., *Nature*, 2020, 586, 549; *Adv. Mater.*, 2014, 26, 4783) where the diffusion of gaseous molecules into the MOFs pores are driven by their spontaneous thermal motion (**Fig. R1a**). As shown in **Fig. R1c**, under visible light irradiation, the HCOOH yield on 1.4 wt.% Ir_1/A-UiO membrane in the spontaneous diffusion mode (**Fig. R1a**) was only 0.76 mmol $g^{-1}h^{-1}$, which is much lower than that obtained in the GMG system that allows the high-flux diffusion of gaseous reactants through the porous catalyst membranes (**Fig. R1b**, HCOOH yield reached 3.38 mmol $g^{-1}h^{-1}$ at the CO_2 flow rate of 120 sccm).

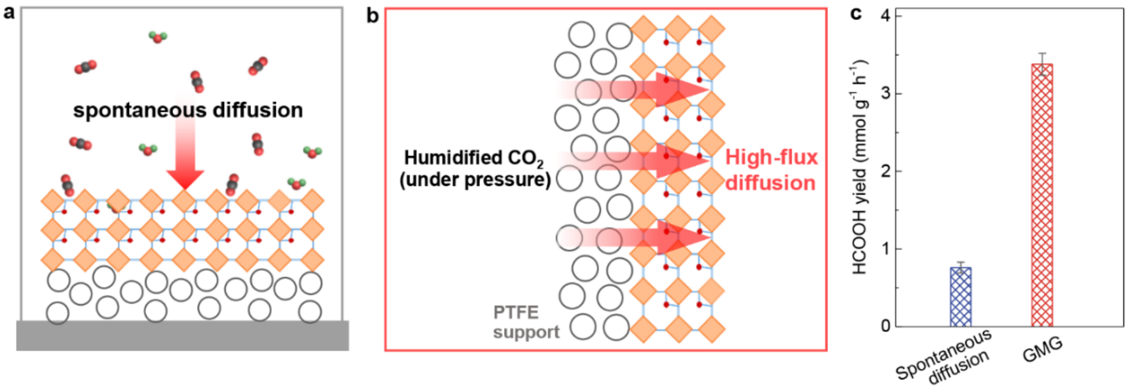

Fig. R1. Photocatalytic CO_2RR in the spontaneous diffusion mode and the GMG mode. (**a**) In the spontaneous diffusion mode, the SA/MOF membrane was placed horizontally in the centre of a 400 ml reaction cell, and air was evacuated before the cell was filled with ultra-pure CO_2. This vacuum and refill procedure was repeated three times, and then the reactor was left for 30 min to establish an adsorption–desorption balance. After that, 3 ml ultrapure H_2O was added to the reactor to provide humidity but without direct contact with the membrane. A 300 W Xe lamp with a 420 nm long-pass filter was employed at the top of the reactor as a light source. (**b**) In the gas-membrane-gas (GMG) mode, humidified CO_2 gas was fed through the SA/MOF membrane, where water vapor was carried by the CO_2 stream. (**c**) HCOOH yields on identical 1.4 wt.% Ir_1/A-aUiO membranes in the conventional spontaneous diffusion mode and GMG mode.

That is, compared with the particle-in-solution (PiS) mode (**Fig. 3a** in the revised MS) (in which CO_2 molecules had to be bubbled into the solution, diffused to the surface of the MOFs particles, and then diffused into the pores within the MOFs) and the spontaneous diffusion mode (**Fig. R1a**) (where the diffusion of CO_2 molecules was driven by thermal motion), the transfer of CO_2 molecules into the pores of MOFs in the GMG mode would much faster, effectively promoting the HCOOH production rate thereby.

Regarding the "higher local CO_2 concentration", there have been several reports on increasing the "local concentration of CO_2" at the catalyst surface in electrochemical CO_2RR by fabricating porous catalyst membranes and enabling the CO_2 feed through the membranes (e.g., *Nat. Catal.*, 2018, 1, 592). Moreover, the local concentration of CO_2 in the porous catalyst layers could be effectively enhanced by increasing the feed flow of CO_2 (e.g., *Joule*, 2020, 4, 1104). Therefore, such previous

reports and our experimental results prove that the MOFs-based GMG interfaces enabling the high-flux CO_2 feed through the porous catalyst membranes can effectively increase the local concentration of CO_2 in the vicinity of the SAs. We have revised the related paragraphs (**Lines 13–16, Page 9**) and cited related literatures. In addition, to avoid possible ambiguity, we have also replaced the term of "local concentration of CO_2" with "CO_2 availability" in the revised manuscript.

We have also revised the SI accordingly (**Supplementary Figure 38**). We hope that our revision and additional data can address the Reviewer's concerns.

R3Q9. Thank you. The use of Mott-Schottky for identification of band edges is disputed for such materials (10.1039/C9TA09569A) though it continues to be used a lot for MOF-based materials. XPS/UPS techniques tend to be more appropriate. When representing the electronic diagram, one should also indicate the redox potential for the water and isopropanol redox couples so the thermodynamic drive for the oxidation reaction (for CO_2) or reduction (for O_2) can be visualised.

Author Reply:

Thanks for the Reviewer's valuable suggestions.

First, we agree with the Reviewer that we should be incredibly careful when using the Mott-Schottky method to identify the band edges of such materials. According to the suggestions of the Reviewer, we have carried out additional UPS characterizations to reveal the band structures of aUiO and A-aUiO (**Fig. R2**) (*Adv. Mater.*, 2016, 28, 6959).

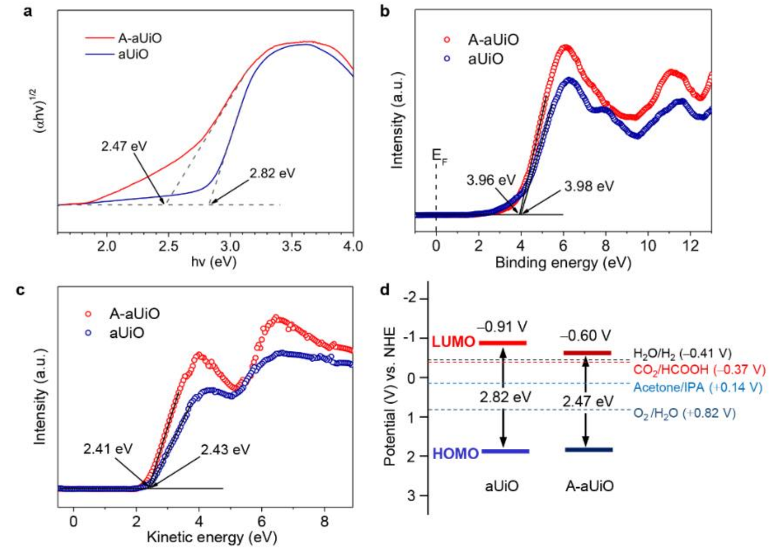

Fig. R2. **Electronic band structures of aUiO samples before and after activation.** (**a**) Optical band gaps determined by UV-vis diffuse reflectance, where α and ν represent the absorbance and wavenumber, respectively. (**b**) Valence-band spectra for A-aUiO and aUiO measured by ultraviolet photoelectron spectroscopy (UPS). Au serves as a reference for E_F set to 0 eV. (**c**) Secondary electron cutoff (E_{cutoff}) in the UPS spectra, from which the work function (Φ) can be calculated. (**d**) Schematic diagrams for conduction bands and band gap energies of aUiO and A-aUiO, and the standard electrode potentials for CO_2/HCOOH, H_2O/H_2, acetone/IPA and O_2/H_2O (vs the NHE at pH = 7).

As shown in **Fig. R2a**, the band gap for aUiO and A-UiO is about 2.82 and 2.47 eV, respectively. From the UPS valence-band spectra (**Fig. R2b,c**), the HOMO energy levels of aUiO and A-aUiO can be calculated to be −6.41 eV and −6.37 eV relative to the vacuum level, respectively (*ACS Nano*, 2014, 8, 8582; *Adv. Mater*, 2016, 28, 6959). That is, the

HOMO energy levels for aUiO and A-aUiO is 1.91 and 1.87 eV versus the normal hydrogen electrode (NHE), respectively. Given the obtained bandgaps of 2.82 and 2.47 eV (**Fig. R2a**), respectively, the conduction band (i.e., LUMO) potential for aUiO and A-aUiO is then calculated to be −0.91 V and −0.60 V vs. NHE, respectively (**Fig. R2d)**. It is noteworthy that the energy-band structures identified by UPS are in well accordance with that obtained from the Mott-Schottky curves (Fig. R4 in our last Response to the Reviewers).

Second, according to the Reviewer's suggestion, to better visualize the thermodynamic drive for the oxidation and reduction reaction, we have indicated the standard redox potential for water (O_2/H_2O = +0.82 V vs. NHE) and isopropanol (acetone/IPA = +0.14 V vs. NHE) (*ACS Appl. Mater. Inter.*, 2019, 11, 24154) in the energy-band diagrams for aUiO and A-aUiO (**Fig. R2d)**.

We have also revised the manuscript accordingly (**Supplementary Figures 17**). We hope these revisions can address the Reviewer's concerns.

R3Q13. Thank you. I suggest adding the porosity measurement after testing as is advised to demonstrate stability of MOF-based structures (besides XRD).

Author Reply:

Thanks for the Reviewer's kind suggestion.

We have performed N_2 adsorption-desorption characterizations (**Fig. R3c,d**) for the photocatalysts after long-term (100 h) operation. As shown in **Fig. R3**, XRD, EXAFS, N_2-sorption, and STEM images together prove that the structures of the Ir_1/A-aUiO catalysts are stable during long-term reactions.

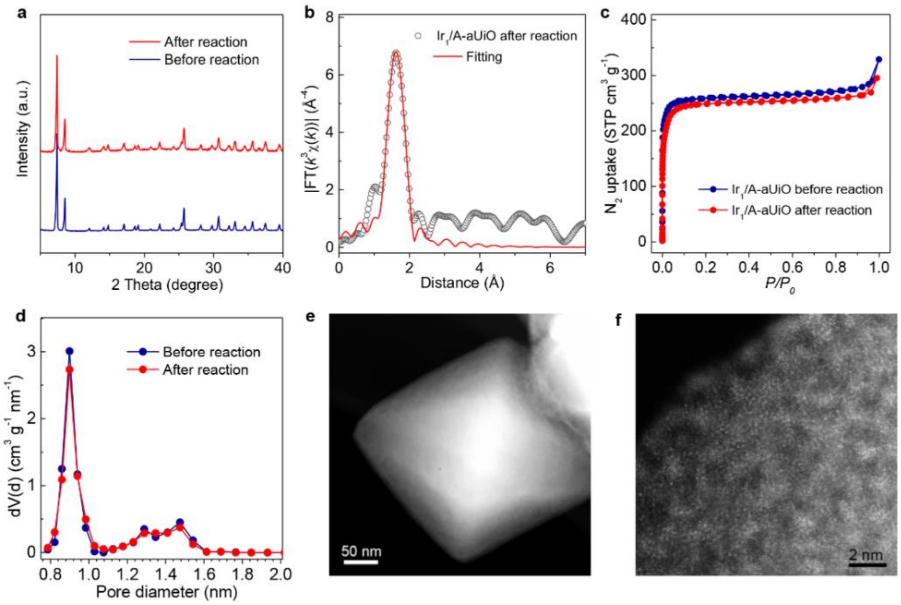

Fig. R3. (**a**) XRD patterns of 1.4 wt.% Ir_1/A-aUiO catalyst before and after long-term operation (100 h). (**b**) Ir L_3-edge EXAFS fitting results for 1.4 wt.% Ir_1/A-aUiO catalyst after long-term operation. The presence of Ir–O coordination but no Ir–Ir signals confirmed the atomic dispersion of Ir species in 1.4 wt.% Ir_1/A-aUiO. (**c**, **d**) N_2 adsorption-desorption isotherms (c) and pore size distributions (d) of Ir_1/A-aUiO catalyst after long-term operation. (**e**, **f**) STEM (**e**) and high-resolution AC-STEM (**f**) images of Ir_1/A-aUiO catalyst after long-term operation.

We have also revised the manuscript accordingly (**Lines 16–19, Page 9 and Supplementary Figure 39**). We hope these revisions can address the Reviewer's concerns.

R3Q14. Thank you. The answer from the authors seem only partial. The authors tested CO_2 reduction using water vapour or isopropanol vapour. When using water, what is the oxidation product observed?

Author Reply:

Thanks for the Reviewer's valuable questions.

The oxidation product is oxygen when using water. And, as shown in **Fig. R4a**, the evolution of O_2 gas (detected by an online gas chromatography, Shimadzu GC-2014C) accompanied the reduction reactions (CO_2-to-HCOOH) in a stoichiometric ratio (the molar ratio between formate and oxygen were about 2.01, 2.08, 2.06, 2.01, and 2.02 at 1, 2, 3, 4, and 5 hours, respectively). In comparison, when introducing isopropanol as the sacrificial reagent, the yield rate for HCOOH could be raised by more than 50 times (from 8.85 to 508.12 μmol g^{-1} h^{-1}) while no O_2 production could be observed (**Fig. R4b**). Instead, acetone was produced, as revealed by the NMR spectra of the reaction solutions before and after CO_2RR tests (**Fig. R4b, c).**

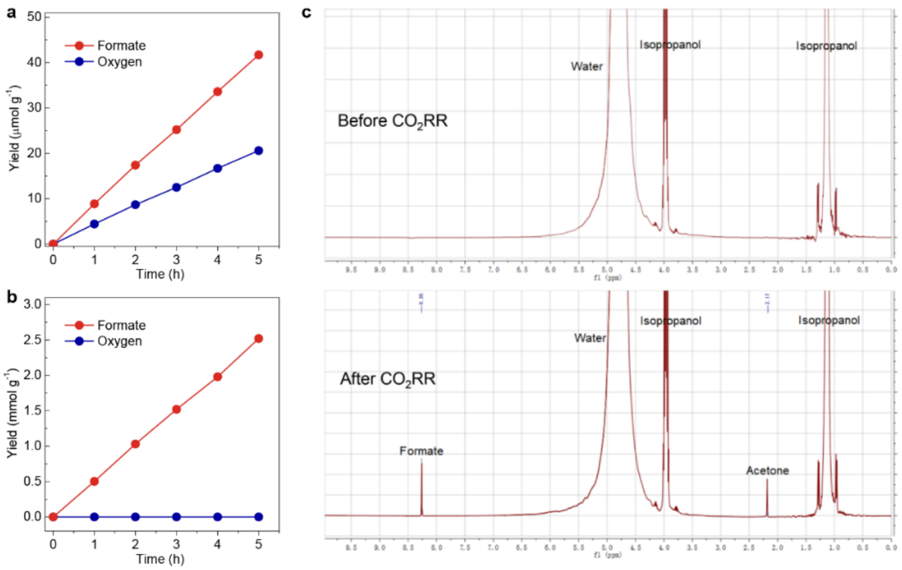

Fig. R4. (**a, b**) Time course of HCOOH and O_2 evolution on Ir_1/A-UiO powder catalysts without (a) and with (b) using isopropanol as sacrificial agent. (**c**) The ^{1}H NMR spectra for the liquid products in the photocatalytic CO_2RR by using isopropanol as sacrificial agent. The peaks at 8.26 and 2.17 ppm can be assigned to $HCOO^-$ (reduction product of CO_2) and acetone (oxidation product of IPA), respectively.

We have also revised the manuscript accordingly (**Supplementary Figure 31**). We hope these revisions can address the Reviewer's concerns.

REVIEWERS' COMMENTS

Reviewer #3 (Remarks to the Author):

The authors have carefully considered the remaining points. Thank you. This is a fine study overall. I have no further comments.
